# Long-term comparative effectiveness and safety of dabigatran, rivaroxaban, apixaban and edoxaban in patients with atrial fibrillation: A nationwide cohort study

**DOI:** 10.3389/fphar.2023.1125576

**Published:** 2023-02-02

**Authors:** Maxim Grymonprez, Tine L. De Backer, Xander Bertels, Stephane Steurbaut, Lies Lahousse

**Affiliations:** ^1^ Department of Bioanalysis, Pharmaceutical Care Unit, Faculty of Pharmaceutical Sciences, Ghent University, Ghent, Belgium; ^2^ Department of Cardiology, Ghent University Hospital, Ghent, Belgium; ^3^ Centre for Pharmaceutical Research, Research group of Clinical Pharmacology and Clinical Pharmacy, Vrije Universiteit Brussel, Jette, Belgium; ^4^ Department of Hospital Pharmacy, Jette, Belgium; ^5^ Department of Epidemiology, Erasmus Medical Center, Rotterdam, Netherlands

**Keywords:** atrial fibrillation, NOAC, VKA, anticoagulant, thromboembolism, bleeding, mortality, myocardial infarction

## Abstract

**Background:** Although non-vitamin K antagonist oral anticoagulants (NOACs) are recommended over vitamin K antagonists (VKAs) in atrial fibrillation (AF) management, direct long-term head-to-head comparisons are lacking. Therefore, their risk-benefit profiles were investigated compared to VKAs and between NOACs.

**Methods:** AF patients initiating anticoagulation between 2013–2019 were identified in Belgian nationwide data. Inverse probability of treatment weighted Cox regression was used to investigate effectiveness and safety outcomes and were additionally stratified by NOAC dose.

**Results:** Among 254,478 AF patients (328,796 person-years of follow-up), NOACs were associated with significantly lower risks of stroke or systemic embolism (stroke/SE) (hazard ratio (HR) 0.68, 95% confidence interval (CI) (0.64–0.72)), all-cause mortality (HR 0.76, 95%CI (0.74–0.79)), major or clinically relevant non-major bleeding (MB/CRNMB) (HR 0.94, 95%CI (0.91–0.98)) and intracranial hemorrhage (HR 0.73, 95%CI (0.66–0.79)), but non-significantly different risks of myocardial infarction, gastrointestinal and urogenital bleeding compared to VKAs. Despite similar stroke/SE risks, dabigatran and apixaban were associated with significantly lower MB/CRNMB risks compared to rivaroxaban (HR 0.86, 95%CI (0.83–0.90); HR 0.86, 95%CI (0.83–0.89), respectively) and edoxaban (HR 0.91, 95%CI (0.83–0.99); HR 0.86, 95%CI (0.81–0.91), respectively), and apixaban with significantly lower major bleeding risks compared to dabigatran (HR 0.86, 95%CI (0.80–0.92)) and edoxaban (HR 0.79, 95%CI (0.72–0.86)). However, higher mortality risks were observed in some risk groups including with apixaban in patients with diabetes or concomitantly using digoxin compared to dabigatran and edoxaban, respectively.

**Conclusion:** NOACs had better long-term risk-benefit profiles than VKAs. While effectiveness was comparable, apixaban was overall associated with a more favorable safety profile followed by dabigatran.

## Introduction

Oral anticoagulants (OACs) are essential in the management of atrial fibrillation (AF) to reduce the associated thromboembolic risk (Steffel et al., 2021). Guidelines recommend the use of non-vitamin K antagonist oral anticoagulants (NOACs) over vitamin K antagonists (VKAs) among patients with AF, given their fast onset of action, fixed dosing regimen without the need for coagulation monitoring, less known interactions, and lower intracranial bleeding risk (Steffel et al., 2021). Following their approval, worldwide anticoagulant use has almost doubled in the last decade, with NOACs being more initiated than VKAs in newly-diagnosed AF patients since 2014 (Grymonprez et al., 2021).

Large randomized controlled trials (RCTs) have demonstrated that NOACs are associated with a non-inferior to superior efficacy and safety compared to VKAs (Connolly et al., 2009; Granger et al., 2011; Patel et al., 2011; Giugliano et al., 2013; Ruff et al., 2014; Carnicelli et al., 2022). However, given their strict eligibility criteria, extrapolation of results to real-life clinical practice may be limited, especially to vulnerable patient subgroups who were underrepresented in trials. Moreover, head-to-head RCTs directly comparing NOACs are currently lacking and indirect head-to-head comparisons in network meta-analyses based on data from RCTs are limited by heterogeneous trial methodologies and study populations. Consequently, sufficient randomized data are lacking to guide the decision-making process of clinicians for choosing an anticoagulant in clinical practice. Therefore, real-world observational studies may be of additional value in addressing this gap in evidence. Previous real-life studies have suggested that the effectiveness of individual NOACs is comparable, but that their safety may differ (Hernandez et al., 2017; Lai et al., 2017; Graham et al., 2019; Durand et al., 2020; Rutherford et al., 2020; Van Ganse et al., 2020; Han et al., 2021; Perreault et al., 2021; Ray et al., 2021). However, these studies were often performed shortly after NOAC approval based on registry data including selective patient populations with a limited follow-up and lack of edoxaban users. Likewise, results were often not stratified by NOAC dose, which may be of importance to guide therapeutic decisions among more vulnerable patients with advanced age, low body weight and/or renal dysfunction, for whom NOAC dose reduction is recommended (Steffel et al., 2021). Furthermore, previous observational studies have also not investigated differences in the risk of upper *versus* lower gastrointestinal bleeding and urogenital bleeding between NOACs, which was identified as a research gap in a systematic review (Cohen et al., 2018).

Therefore, we aimed to investigate the effectiveness and safety of dabigatran, rivaroxaban, apixaban and edoxaban in direct head-to-head comparisons with VKAs and between individual NOACs in unselected patients with AF on a full-population scale from 2013 to 2019. Differences between standard and reduced dose NOACs and in location-specific bleeding risks were explored.

## Materials and methods

### Source population

Data were provided by two nationwide databases, the InterMutualistic Agency (IMA) database and Minimal Hospital Dataset (MHD), as described before (Grymonprez et al., 2022). The IMA centralizes all claims data from Belgian health insurance funds on reimbursed ambulatory and hospital care, including demographic characteristics (age, sex, date of death), medical procedures (diagnostic or therapeutic procedures and other reimbursed care) and drug prescription claims (Grymonprez et al., 2022; IMA/AIM, 2021). As health insurance is legally mandatory in Belgium, the source population represents all legal residents with reimbursed medication or care. The MHD aggregates hospital discharge diagnoses of every hospital admission in Belgium, coded in International Classification of Diseases (ICD) codes (ICD-9 up to 2014, ICD-10 from 2015 onwards) (MZG, 2021; Grymonprez et al., 2022). This study was approved by the IMA and MHD database administrators as well as by the ‘Sectoral Committee of Social Security and Health, Section Health’, a subcommittee of the Belgian Commission for the Protection of Privacy (approval code IVC/KSZG/20/344) (eHealth-platform, 2021). A more detailed description of the source population is provided in the supplemental materials. This study followed the Strengthening the Reporting of Observational Studies in Epidemiology (STROBE) reporting guideline (Supplemental eTable S1) (von Elm et al., 2007).

### Study population

From 1 January 2013 to 1 January 2019, subjects ≥45 years old and with ≥1 year coverage by a Belgian health insurance fund were included from the IMA database on the first date of filling an OAC prescription in ambulatory or hospital care (=index date) (Supplemental eFigure S1). Users of NOACs, namely dabigatran (approved in Belgium since August 2012), rivaroxaban (approved since September 2012), apixaban (approved since September 2013) and edoxaban (approved since October 2016), and VKAs (warfarin, acenocoumarol, phenprocoumon) were included (Grymonprez et al., 2022). Only OAC-naïve patients were considered, excluding OAC-experienced subjects with an OAC prescription filled ≤1 year before the index date. In order to specifically include non-valvular AF patients and avoid competing treatment indications, subjects were excluded in case of total hip or knee replacement, or diagnosis of deep vein thrombosis or pulmonary embolism ≤6 months before the index date, based on specific ICD and/or medical procedure codes (Supplemental eTable S2). Moreover, only AF patients eligible for NOACs and VKAs were examined, excluding subjects with valvular AF (mechanical prosthetic heart valve or moderate/severe mitral stenosis) and/or end-stage renal disease (chronic kidney disease (CKD) stage V and/or dialysis). Lastly, subjects with ≥2 prescription claims of different types or doses of OACs on the index date, or subjects treated with NOAC doses not approved for stroke prevention in AF (e.g., rivaroxaban 10 mg) were excluded.

### Outcomes

Outcomes were identified using ICD-coded hospital discharge diagnoses (e.g., cerebral infarction) and specific medical procedure codes in hospital care (e.g., intracranial mechanical thrombectomy) from the day after OAC initiation (summarized in Supplemental eTable S3) (Grymonprez et al., 2022). The incident date of outcomes was defined as the date of hospital admission for ICD codes and date of registration for medical procedure codes, whichever occurred first. Each outcome was evaluated separately. Primary effectiveness and safety outcomes included a composite of stroke or systemic embolism (stroke/SE), and major or clinically relevant non-major bleeding (MB/CRNMB), respectively. Major bleeding was defined as a hospitalized bleeding event in a critical area or organ (e.g., intracranial), fatal bleeding or bleeding event with a medical procedure code for blood transfusion ≤10 days after admission (Halvorsen et al., 2017; Rutherford et al., 2020). This definition is adapted from the definition of the International Society on Thrombosis and Haemostasis (ISTH) (Kaatz et al., 2015), considering that no data on hemoglobin levels or number of blood transfusion units were available (Kaatz et al., 2015; Halvorsen et al., 2017; Rutherford et al., 2020). CRNMB was defined as a bleeding event requiring hospitalization that did not classify for major bleeding. Secondary effectiveness outcomes included any stroke (ischemic, hemorrhagic or unspecified), ischemic stroke, non-cerebral systemic embolism (SE), all-cause mortality and acute myocardial infarction. Secondary safety outcomes included major bleeding; CRNMB; intracranial hemorrhage; any, upper or lower gastrointestinal bleeding; urogenital bleeding; and bleeding from other sites (e.g., retroperitoneal).

### Follow-up

Patients were followed from OAC initiation until the first occurrence of the investigated outcome, discontinuation (>60-day gap of drug supply) or switch of treatment, death, emigration or end of the study period (1 January 2019), whichever came first (on-treatment analysis) (see Supplemental Material for more detailed information on follow-up).

### Covariates

Baseline characteristics were assessed on the index date and included age, sex, comorbidities, medication history and clinical risk scores. Comorbidities were identified with specific ICD-coded diagnoses, medical procedure codes and/or drug prescription claims ≤1 year before the index date (summarized in Supplemental eTable S2). Medication history was identified with drug prescription claims, considering recent use ≤6 months before the index date. Moreover, the CHA_2_DS_2_-VASc score, HAS-BLED score and age-adjusted Charlson Comorbidity Index were calculated (Quan et al., 2011; Steffel et al., 2021). Due to missing laboratory values, a modified HAS-BLED score was used without the “labile INR” criterion. Frailty was identified using the Claims-based Frailty Indicator (Segal et al., 2017). More details on covariate definitions are provided in the supplemental materials and Supplemental eTable S2.

### Statistical analyses

Mean and standard deviation, and counts and percentages were presented for continuous and categorical variables, respectively. Missing ICD data of covariates (due to the transition from ICD-9 to ICD-10 in 2015) were accounted for through multiple imputation by chained equations. Crude event rates per outcome were calculated as the total number of events per 100 person-years at risk. Outcomes were compared between NOACs and VKAs, and between individual NOACs using stabilized inverse probability of treatment weighting (IPTW) to minimize confounding by indication. In comparisons with apixaban and edoxaban, the study population was restricted to subjects having initiated treatment from September 2013 and from October 2016 onwards respectively, to improve comparability and avoid violations of the positivity assumption (as it was impossible for subjects treated before these dates to get apixaban or edoxaban prescribed, given their respective approval dates) (Austin and Stuart, 2015; Villines et al., 2019). Propensity scores (PS) were calculated with logistic regression models including the 37 confounding covariates described in Table 1, which have been selected based on their potential association with the outcomes and treatment (baseline demographics, comorbidities, medication history and clinical risk scores), stratified by calendar year (to account for changes in prescribing practices over time) (Halvorsen et al., 2021). Separate PS models were derived for each comparison. Based on the PS, stabilized weights were calculated to estimate the population average treatment effect and were truncated at the 0.5th and 99.5th percentile to reduce the impact of extreme weights. Covariate balance before and after weighting was checked using standardized mean differences (SMDs) with a ≥0.1 threshold to indicate imbalance, and graphically presented in Love plots. Weighted Cox proportional hazard regression models were used to calculate adjusted hazard ratios (aHRs) with 95% confidence intervals (CIs) using robust sandwich variance estimators to account for weighting-induced dependencies (Ray et al., 2021). Unbalanced variables were incorporated in the Cox regression model. The proportional hazard assumption was tested using scaled Schoenfeld residuals. In case of potential non-proportionality, a stratified Cox regression model was performed. Results were visually presented in forest plots. A two-sided *p*-value of <0.05 was considered statistically significant. All analyses were performed in R (R version 3.6.0).

**TABLE 1:** Baseline characteristics of OAC-naïve AF patients.

Patient characteristics	VKA (n = 61,406)	NOAC					SMD^*^	
		Overall (n = 193,072)	Dabigatran (n = 28,144)	Rivaroxaban (n = 74,421)	Apixaban (n = 66,925)	Edoxaban (n = 23,582)	Before IPTW	After IPTW
Age (years)	70.9±12.1	76.3±10.1	76.0±9.8	75.6±10.4	77.3±9.8	75.9±10.3	0.48	0.016
<65 years	21,158 (34.5%)	24,741 (12.8%)	3,632 (12.9%)	10,841 (14.6%)	6,940 (10.4%)	3,328 (14.1%)	NA	NA
65–74 years	15,128 (24.6%)	55,706 (28.9%)	8,337 (29.6%)	21,695 (29.2%)	18,462 (27.6%)	7,212 (30.6%)		
75–84 years	16,798 (27.4%)	72,517 (37.6%)	10,891 (38.7%)	27,754 (37.3%)	25,614 (38.3%)	8,258 (35.0%)		
≥85 years	8,322 (13.6%)	40,108 (20.8%)	5,284 (18.8%)	14,131 (19.0%)	15,909 (23.8%)	4,784 (20.3%)		
Female	28,766 (46.8%)	92,008 (47.7%)	13,130 (46.7%)	35,188 (47.3%)	32,892 (49.1%)	10,798 (45.8%)	0.016	0.006
Reduced dose	0 (0.0%)	71,842 (37.2%)	15,482 (55.0%)	29,718 (39.9%)	19,664 (29.4%)	6,978 (29.6%)	NA	NA
Follow-up (years)	0.8±1.3	1.4±1.5	1.6±1.7	1.6±1.6	1.4±1.3	0.7±0.6	NA	NA
**Comorbidities**								
Hypertension	36,945 (60.2%)	127,931 (66.3%)	18,382 (65.3%)	47,785 (64.2%)	46,477 (69.4%)	15,287 (64.8%)	0.127	0.01
Coronary artery disease	14,142 (23.0%)	33,701 (17.5%)	4,314 (15.3%)	12,263 (16.5%)	12,928 (19.3%)	4,196 (17.8%)	0.137	0.013
Congestive heart failure	9,779 (15.9%)	30,114 (15.6%)	3,581 (12.7%)	10,841 (14.6%)	12,097 (18.1%)	3,595 (15.2%)	0.007	0.023
Valvular heart disease	11,889 (19.4%)	24,273 (12.6%)	3,109 (11.0%)	8,145 (10.9%)	9,673 (14.5%)	3,346 (14.2%)	0.191	0.002
Peripheral artery disease	6,866 (11.2%)	14,070 (7.3%)	1866 (6.6%)	4,865 (6.5%)	5,757 (8.6%)	1,582 (6.7%)	0.126	0.004
Dyslipidemia	34,288 (55.8%)	109,627 (56.8%)	16,333 (58.0%)	40,510 (54.4%)	39,373 (58.8%)	13,411 (56.9%)	0.019	0.003
Chronic kidney disease	8,607 (14.0%)	20,888 (10.8%)	1861 (6.6%)	6,869 (9.2%)	9,284 (13.9%)	2,873 (12.2%)	0.088	0.008
Chronic liver disease	2,563 (4.2%)	5,894 (3.1%)	738 (2.6%)	2,235 (3.0%)	2,175 (3.3%)	745 (3.2%)	0.053	0.001
Chronic lung disease	8,428 (13.7%)	23,618 (12.2%)	3,039 (10.8%)	8,928 (12.0%)	8,854 (13.2%)	2,796 (11.9%)	0.037	0.001
Obstructive sleep apnea	2,280 (3.7%)	6,493 (3.4%)	879 (3.1%)	2,481 (3.3%)	2,279 (3.4%)	854 (3.6%)	0.014	0.012
Cancer	5,939 (9.7%)	19,248 (10.0%)	2,480 (8.8%)	7,369 (9.9%)	6,930 (10.4%)	2,470 (10.5%)	0.016	0.011
Upper GI tract disorder^**^	5,163 (8.4%)	14,015 (7.3%)	1749 (6.2%)	5,397 (7.3%)	5,405 (8.1%)	1,464 (6.2%)	0.036	0.008
Lower GI tract disorder^**^	4,175 (6.8%)	13,482 (7.0%)	1791 (6.4%)	5,106 (6.9%)	4,942 (7.4%)	1,643 (7.0%)	0.016	0.004
Diabetes mellitus	22,453 (36.6%)	60,250 (31.2%)	7,890 (28.0%)	21,922 (29.5%)	23,051 (34.4%)	7,387 (31.3%)	0.112	0.028
Anemia	6,485 (10.6%)	14,627 (7.6%)	1,626 (5.8%)	5,281 (7.1%)	5,975 (8.9%)	1745 (7.4%)	0.094	0.004
Dementia	2,809 (4.6%)	10,753 (5.6%)	1,297 (4.6%)	4,046 (5.4%)	4,312 (6.4%)	1,098 (4.7%)	0.051	0.018
History of falling	4,003 (6.5%)	16,170 (8.4%)	1817 (6.5%)	5,290 (7.1%)	6,930 (10.4%)	2,134 (9.0%)	0.08	0.035
Frailty	13,746 (22.4%)	58,738 (30.4%)	7,735 (27.5%)	20,840 (28.0%)	23,623 (35.3%)	6,540 (27.7%)	0.187	0.024
Prior stroke/SE	8,759 (14.3%)	26,635 (13.8%)	4,875 (17.3%)	7,815 (10.5%)	11,697 (17.5%)	2,249 (9.5%)	0.004	0.013
Prior MB/CRNMB	4,008 (6.5%)	10,270 (5.3%)	1,379 (4.9%)	3,569 (4.8%)	4,153 (6.2%)	1,170 (5.0%)	0.04	0.005
**Medication history**								
Number of concomitant drugs	6.8±4.5	6.6±4.1	6.3±3.8	6.5±4.1	7.0±4.3	6.4±4.0	0.049	0.004
Beta blockers	31,820 (51.8%)	119,997 (62.2%)	17,201 (61.1%)	44,678 (60.0%)	43,133 (64.4%)	14,985 (63.5%)	0.21	0.008
Verapamil, diltiazem	2,162 (3.5%)	7,741 (4.0%)	1,147 (4.1%)	3,192 (4.3%)	2,627 (3.9%)	775 (3.3%)	0.026	0.017
Digoxin	3,694 (6.0%)	18,837 (9.8%)	2,564 (9.1%)	6,934 (9.3%)	7,111 (10.6%)	2,228 (9.4%)	0.139	0.011
Class I AAD	3,490 (5.7%)	19,811 (10.3%)	3,068 (10.9%)	8,196 (11.0%)	6,024 (9.0%)	2,523 (10.7%)	0.17	0.007
Class III AAD	11,578 (18.9%)	49,873 (25.8%)	7,242 (25.7%)	20,148 (27.1%)	17,217 (25.7%)	5,266 (22.3%)	0.168	0.027
Acetylsalicylic acid	21,648 (35.3%)	78,333 (40.6%)	11,430 (40.6%)	29,564 (39.7%)	28,060 (41.9%)	9,279 (39.3%)	0.11	0.007
P2Y12 inhibitor	3,391 (5.5%)	11,290 (5.8%)	1,495 (5.3%)	3,869 (5.2%)	4,233 (6.3%)	1,693 (7.2%)	0.014	0.011
Proton pump inhibitor	25,707 (41.9%)	76,541 (39.6%)	10,448 (37.1%)	28,919 (38.9%)	27,865 (41.6%)	9,309 (39.5%)	0.045	0.016
NSAID	16,501 (26.9%)	46,481 (24.1%)	6,856 (24.4%)	18,565 (24.9%)	15,513 (23.2%)	5,547 (23.5%)	0.064	0.005
Oral corticosteroids	13,892 (22.6%)	38,247 (19.8%)	5,109 (18.2%)	15,002 (20.2%)	13,507 (20.2%)	4,629 (19.6%)	0.069	0.001
SSRI/SNRI	8,233 (13.4%)	23,094 (12.0%)	3,283 (11.7%)	9,148 (12.3%)	8,336 (12.5%)	2,327 (9.9%)	0.043	0.014
**Clinical risk score**								
CHA_2_DS_2_-VASc score	3.2±2.0	3.6±1.8	3.5±1.7	3.4±1.7	3.8±1.8	3.4±1.7	0.183	0.015
HAS-BLED score	2.3±1.4	2.5±1.2	2.5±1.2	2.4±1.2	2.7±1.2	2.5±1.2	0.184	0.011
Charlson Comorbidity Index	4.0±2.5	4.4±2.2	4.3±2.1	4.3±2.2	4.7±2.2	4.3±2.2	0.183	0.006

Data shown as mean ± standard deviation, or counts and percentages. VKA users included 29,650 acenocoumarol, 16,859 warfarin and 14,897 phenprocoumon users.

^a^ Absolute SMDs, illustrated for comparison of NOACs, *versus* VKAs, before and after inverse probability of treatment weighting.

^b^ Upper and lower gastrointestinal tract disorders were defined as gastroesophageal reflux disease or peptic ulcer disease; and diverticulosis, angiodysplasia, colorectal polyposis or hemorrhoids, respectively. AAD: antiarrhythmic drug; AF: atrial fibrillation; CRNMB: clinically relevant non-major bleeding; GI: gastrointestinal; MB: major bleeding; NA: not applicable; NOAC: non-vitamin K antagonist oral anticoagulant; NSAID: non-steroidal anti-inflammatory drug; OAC: oral anticoagulant; SE: systemic embolism; SMD: standardized mean difference; SNRI: serotonin and norepinephrine reuptake inhibitor; SSRI: selective serotonin reuptake inhibitor; VKA: vitamin K antagonist.

### Subgroup analyses

As a subgroup analysis, outcomes were investigated with standard (dabigatran 2 × 150 mg, rivaroxaban 1 × 20 mg, apixaban 2 × 5 mg, edoxaban 1 × 60 mg) and reduced dose NOACs (dabigatran 2 × 110 mg, rivaroxaban 1 × 15 mg, apixaban 2 × 2.5 mg, edoxaban 1 × 30 mg) separately.

### Sensitivity analyses

Several sensitivity analyses were performed to check the robustness of results. First, analyses were repeated with 1:1 PS matching (PSM) using nearest neighbor matching without replacement and a caliper of 0.05. Second, to examine whether estimates were affected by differential censoring between treatment groups (e.g., due to differences in discontinuation or switching rates) (Yao et al., 2016), analyses were repeated using an intention-to-treat approach, defining the end of follow-up as the first occurrence of an outcome, death, emigration or end of study period, whichever occurred first. Third, only subjects with an ICD-coded diagnosis of AF before or up to 90 days after the index date (to account for diagnostic lag) were investigated to increase the likelihood of treatment indication, although this approach resulted in the exclusion of AF patients treated exclusively in primary or ambulatory care (Hellfritzsch et al., 2020). Fourth, the study population was restricted to subjects having initiated treatment between 1 October 2016 and 1 January 2019, when all NOACs were commercially available in Belgium, to avoid time-period bias and account for the shorter follow-up of edoxaban compared to other NOACs (Yao et al., 2016; Graham et al., 2019). Fifth, although data were lacking on the cause of death, the risk of AF-related mortality was investigated as an exploratory analysis, by only considering deaths occurring within 60 days after an event of thromboembolism, bleeding or myocardial infarction. Lastly, as an exploratory *post hoc* analysis, significant interactions between treatment and covariates for the risk of mortality between individual NOACs were explored using doubly robust estimation (DRE) models.

## Results

### Baseline characteristics

**FIGURE 1:**
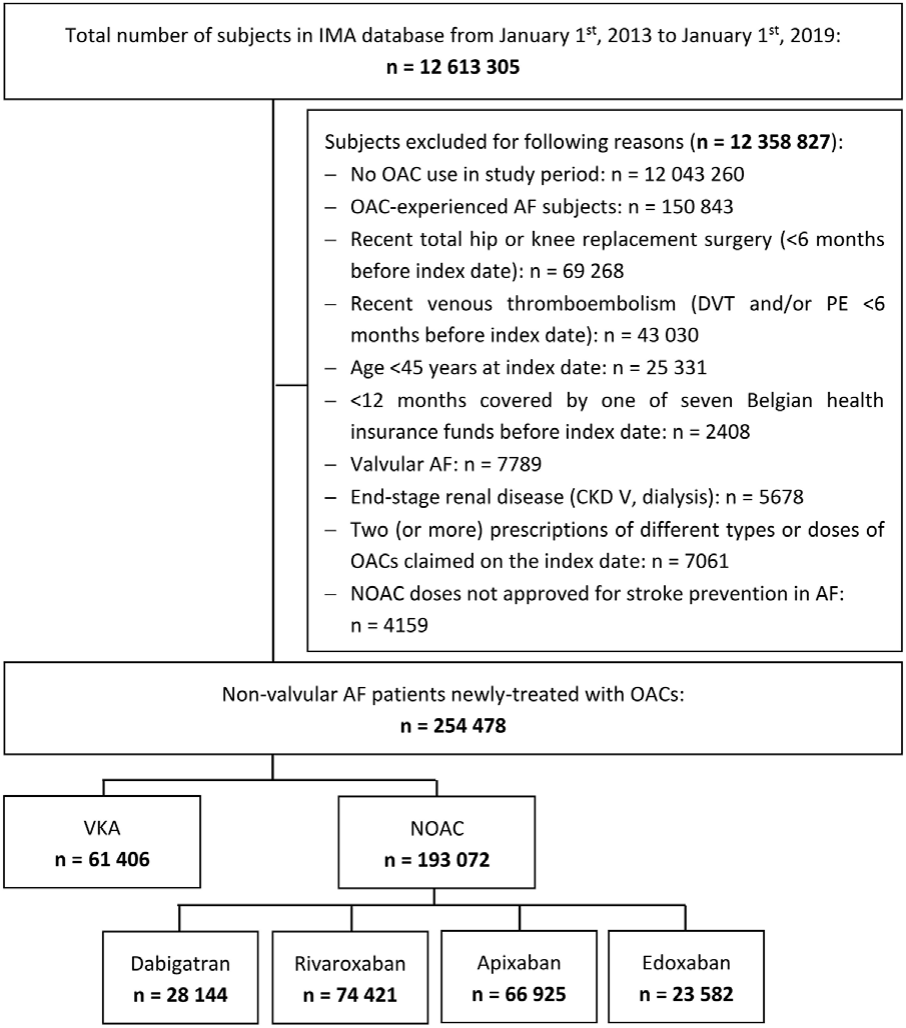
Flowchart of study population. AF: atrial fibrillation; CKD: chronic kidney disease; DVT: deep vein thrombosis; IMA: InterMutualistic Agency; NOAC: non-vitamin K antagonist oral anticoagulant; OAC: oral anticoagulant; PE: pulmonary embolism; VKA: vitamin K antagonist.

A total of 254,478 newly-treated AF patients were included during a mean follow-up of 1.3 ± 1.5 years (total of 328,796 person-years of on-treatment follow-up; 37,499 (14.7%) and 8,534 (3.4%) subjects with ≥3 and ≥5 years of on-treatment follow-up, respectively) (Figure 1). Baseline characteristics of the 193,072 NOAC- and 61,406 VKA-treated AF patients are summarized in Table 1. Before weighting, NOAC and VKA users were on average 76.3 ± 10.1 and 70.9 ± 12.1 years old and had a mean CHA_2_DS_2_-VASc score of 3.6 ± 1.8 and 3.2 ± 2.0, respectively. Apixaban users were older (77.3 ± 9.8 years) and had higher CHA_2_DS_2_-VASc (3.8 ± 1.8) and HAS-BLED scores (2.7 ± 1.2) than users of other NOACs or VKAs. After weighting, covariate balance was achieved (Table 1, Supplemental eFigure S2).

### NOAC *versus* VKA

**FIGURE 2:**
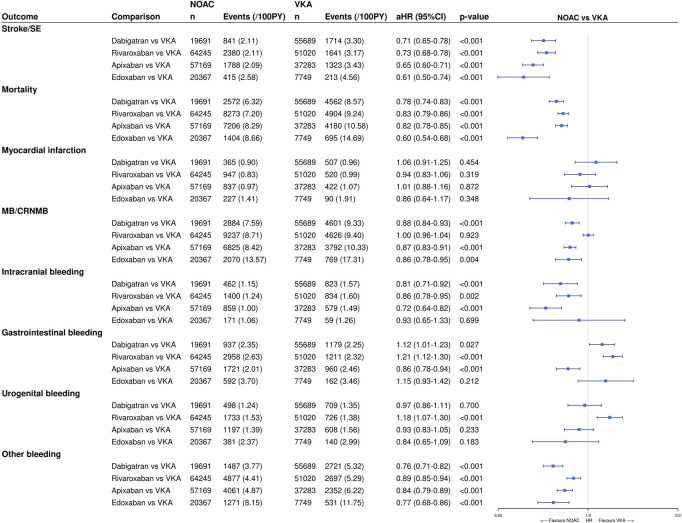
Effectiveness and safety of individual NOACs *versus* VKAs after IPTW. The weighted number of subjects at risk in the pseudopopulation, weighted number of events, weighted event rates per 100 PY and adjusted HRs with 95%CIs after IPTW are illustrated. CI: confidence interval; CRNMB: clinically relevant non-major bleeding; (a)HR: (adjusted) hazard ratio; IPTW: inverse probability of treatment weighting; MB: major bleeding; NOAC: non-vitamin K antagonist oral anticoagulant; PY: person-years; Ref: reference category; SE: systemic embolism; VKA: vitamin K antagonist; vs. *versus*.

The number of events and unadjusted event rates are summarized in Table 2. In terms of effectiveness, NOACs were associated with significantly lower risks of stroke/SE (aHR 0.68, 95%CI (0.64–0.72)), any stroke (aHR 0.73, 95%CI (0.68–0.78)), ischemic stroke (aHR 0.66, 95%CI (0.61–0.72)), SE (aHR 0.53, 95%CI (0.47–0.60)) and all-cause mortality (aHR 0.76, 95%CI (0.74–0.79)) compared to VKAs after multivariable adjustment, while the risk of myocardial infarction was not significantly different (aHR 0.94, 95%CI (0.84–1.06)) (eTable 4). Likewise, dabigatran, rivaroxaban, apixaban and edoxaban were associated with significantly lower risks of stroke/SE, any stroke, ischemic stroke, SE and all-cause mortality, and a non-significantly different risk of myocardial infarction compared to VKAs (Figure 2).

**TABLE 2:** The number of events and crude event rates per 100 person-years of outcomes.

	VKA	NOAC	Dabigatran	Rivaroxaban	Apixaban	Edoxaban
Outcome	Events (per 100 PY)	Events (per 100 PY)	Events (per 100 PY)	Events (per 100 PY)	Events (per 100 PY)	Events (per 100 PY)
**Effectiveness**						
Stroke or systemic embolism	1,660 (3.27)	5,720 (2.10)	909 (2.03)	2,467 (2.06)	1952 (2.16)	392 (2.34)
Stroke	1,201 (2.35)	4,905 (1.80)	778 (1.74)	2,114 (1.76)	1700 (1.88)	313 (1.86)
Ischemic stroke	843 (1.64)	3,052 (1.11)	542 (1.20)	1,310 (1.08)	1,015 (1.11)	185 (1.10)
Systemic embolism	498 (0.97)	952 (0.34)	161 (0.35)	406 (0.33)	294 (0.32)	91 (0.54)
All-cause mortality	4,264 (8.19)	20,589 (7.44)	2,687 (5.89)	8,688 (7.12)	7,850 (8.52)	1,364 (8.06)
Myocardial infarction	497 (0.96)	2,392 (0.87)	347 (0.76)	948 (0.78)	878 (0.96)	219 (1.30)
**Safety**						
MB/CRNMB	4,604 (9.57)	21,397 (8.30)	2,956 (6.90)	9,475 (8.40)	6,981 (8.10)	1985 (12.40)
Major bleeding	2,686 (5.39)	12,030 (4.51)	1800 (4.09)	5,609 (4.80)	3,616 (4.04)	1,005 (6.08)
CRNMB	2,464 (4.93)	11,705 (4.40)	1,479 (3.34)	4,943 (4.22)	4,102 (4.63)	1,181 (7.23)
Intracranial hemorrhage	791 (1.55)	3,011 (1.10)	476 (1.06)	1,443 (1.20)	915 (1.00)	177 (1.05)
Gastrointestinal bleeding	1,170 (2.28)	6,483 (2.38)	1,002 (2.23)	3,094 (2.59)	1806 (1.99)	581 (3.48)
Upper gastrointestinal bleeding	584 (1.13)	3,161 (1.15)	461 (1.02)	1,440 (1.19)	971 (1.06)	289 (1.72)
Lower gastrointestinal bleeding	755 (1.47)	4,091 (1.50)	638 (1.41)	2062 (1.71)	1,047 (1.15)	344 (2.05)
Urogenital bleeding	681 (1.32)	4,061 (1.49)	563 (1.25)	1825 (1.52)	1,300 (1.43)	373 (2.23)
Bleeding from other sites	2,789 (5.60)	11,490 (4.30)	1,435 (3.23)	4,872 (4.14)	4,018 (4.53)	1,165 (7.12)

CRNMB: clinically relevant non-major bleeding; MB: major bleeding; NOAC: non-vitamin K antagonist oral anticoagulant; PY: person-year; VKA: vitamin K antagonist.

Regarding safety outcomes, NOACs were associated with significantly lower risks of MB/CRNMB (aHR 0.94, 95%CI (0.91–0.98)), major bleeding (aHR 0.88, 95%CI (0.84–0.92)), intracranial hemorrhage (aHR 0.73, 95%CI (0.66–0.79)) and bleeding from other sites (aHR 0.89, 95%CI (0.85–0.94)) after multivariable adjustment (Supplemental eTable S4). Risks of CRNMB (aHR 0.99, 95%CI (0.94–1.04)); any, upper or lower gastrointestinal bleeding (aHR 1.06, 95%CI (0.98–1.14), aHR 1.05, 95%CI (0.95–1.16), aHR 1.02, 95%CI (0.93–1.12), respectively); and urogenital bleeding (aHR 1.07, 95%CI (0.98–1.18)) were not significantly different compared to VKAs. Dabigatran, apixaban and edoxaban were associated with significantly lower risks of MB/CRNMB (aHR 0.88, 95%CI (0.84–0.93); aHR 0.87, 95%CI (0.83–0.91); aHR 0.86, 95%CI (0.78–0.95), respectively) and CRNMB (aHR 0.85, 95%CI (0.79–0.92); aHR 0.92, 95%CI (0.87–0.98); aHR 0.82, 95%CI (0.72–0.93), respectively), and dabigatran and apixaban also with significantly lower risks of major bleeding (aHR 0.91, 95%CI (0.85–0.97); aHR 0.78, 95%CI (0.73–0.83), respectively) compared to VKAs (Figure 2). The risks of MB/CRNMB, major bleeding and CRNMB were not significantly different when comparing rivaroxaban to VKAs. All NOACs were associated with significantly lower risks of intracranial hemorrhage and bleeding from other sites compared to VKAs, except for a non-significantly lower intracranial hemorrhage risk with edoxaban (aHR 0.93, 95%CI (0.65–1.33)). Compared to VKAs, the risk of gastrointestinal bleeding was significantly higher with dabigatran (aHR 1.12, 95%CI (1.01–1.23)) and rivaroxaban (aHR 1.21, 95%CI (1.12–1.30)), and non-significantly higher with edoxaban (aHR 1.15, 95%CI (0.93–1.42)), while significantly lower with apixaban (aHR 0.86, 95%CI (0.78–0.94)), driven by a lower risk of lower gastrointestinal bleeding. No significant differences in the risk of urogenital bleeding between NOACs and VKAs were observed, except for a significantly higher risk with rivaroxaban compared to VKAs (aHR 1.18, 95%CI (1.07–1.30)).

### NOAC *versus* NOAC

**FIGURE 3:**
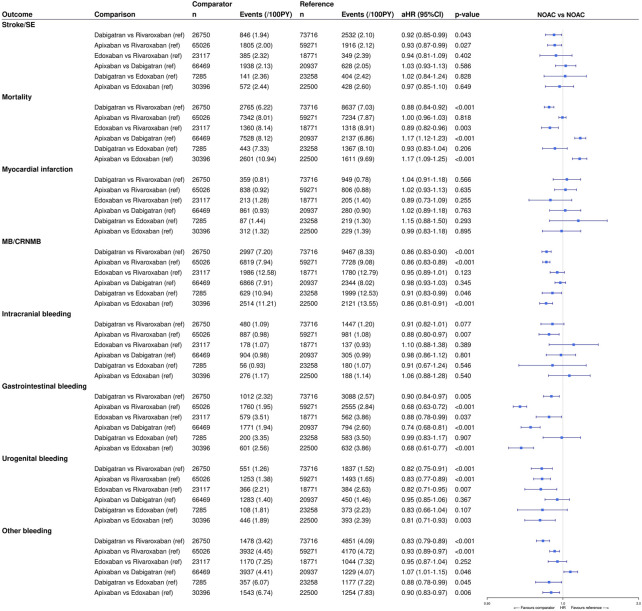
Effectiveness and safety compared between individual NOACs types after IPTW. The weighted number of subjects at risk in the pseudopopulation, weighted number of events, weighted event rates per 100 PY and adjusted HRs with 95%CIs after IPTW are illustrated. CI: confidence interval; CRNMB: clinically relevant non-major bleeding; (a)HR: (adjusted) hazard ratio; IPTW: inverse probability of treatment weighting; MB: major bleeding; NOAC: non-vitamin K antagonist oral anticoagulant; PY: person-years; Ref: reference category; SE: systemic embolism; vs. *versus*.

No significant differences in the risks of stroke/SE, any stroke, ischemic stroke, SE and myocardial infarction were observed between individual NOACs, except for significantly lower risks of stroke/SE with dabigatran (aHR 0.92, 95%CI (0.85–0.99)) and apixaban (aHR 0.93, 95%CI (0.87–0.99)) compared to rivaroxaban (Supplemental eTable S5, Figure 3). Dabigatran (aHR 0.88, 95%CI (0.84–0.92)) and edoxaban (aHR 0.89, 95%CI (0.82–0.96)) were associated with significantly lower risks of all-cause mortality compared to rivaroxaban, whereas apixaban was associated with a higher mortality risk compared to dabigatran (aHR 1.17, 95%CI (1.12–1.23)) and edoxaban (aHR 1.17, 95%CI (1.09–1.25)). No significant difference in the risk of death was observed between dabigatran and edoxaban, and apixaban and rivaroxaban.

In terms of safety, dabigatran and apixaban were associated with significantly lower risks of MB/CRNMB (aHR 0.86, 95%CI (0.83–0.90); aHR 0.86, 95%CI (0.83–0.89), respectively), major bleeding (aHR 0.89, 95%CI (0.85–0.94); aHR 0.77, 95%CI (0.74–0.81), respectively) and CRNMB (aHR 0.83, 95%CI (0.79–0.89); aHR 0.94, 95%CI (0.90–0.98), respectively) compared to rivaroxaban, driven by significantly lower risks of gastrointestinal bleeding (aHR 0.90, 95%CI (0.84–0.97); aHR 0.68, 95%CI (0.63–0.72), respectively), urogenital bleeding (aHR 0.82, 95%CI (0.75–0.91); aHR 0.83, 95%CI (0.77–0.89), respectively), and bleeding from other sites (aHR 0.83 (0.79–0.89); aHR 0.93 (0.89–0.97), respectively) (Supplemental eTable S5, Figure 3). Dabigatran and apixaban were also associated with significantly lower risks of MB/CRNMB (aHR 0.91, 95%CI (0.83–0.99); aHR 0.86, 95%CI (0.81–0.91), respectively) and CRNMB (aHR 0.85, 95%CI (0.75–0.96); aHR 0.91, 95%CI (0.85–0.99), respectively) compared to edoxaban, and apixaban with significantly lower risks of major bleeding (aHR 0.79, 95%CI (0.72–0.86)) compared to edoxaban. This was driven by significantly lower risks of bleeding from other sites with dabigatran (aHR 0.88, 95%CI (0.78–0.99)) and apixaban (aHR 0.90, 95%CI (0.83–0.97)) compared to edoxaban, and by significantly lower risks of gastrointestinal bleeding (aHR 0.68, 95%CI (0.61–0.77)) and urogenital bleeding (aHR 0.81, 95%CI (0.71–0.93)) with apixaban compared to edoxaban. Compared to dabigatran, apixaban was also associated with significantly lower risks of major bleeding (aHR 0.86, 95%CI (0.80–0.92)) and gastrointestinal bleeding (aHR 0.74, 95%CI (0.68–0.81)), whereas with significantly higher risks of CRNMB (aHR 1.09, 95%CI (1.02–1.17)) and bleeding from other sites (aHR 1.07, 95%CI (1.01–1.15)). No differences in the risks of MB/CRNMB, major bleeding and CRNMB were observed between edoxaban and rivaroxaban, and in the risk of major bleeding between edoxaban and dabigatran. However, edoxaban was associated with significantly lower risks of gastrointestinal (aHR 0.88, 95%CI (0.78–0.99)) and urogenital bleeding (aHR 0.82, 95%CI (0.71–0.95)) compared to rivaroxaban. No significant differences in the risk of intracranial hemorrhage were observed between individual NOACs, except for a significantly lower risk with apixaban compared to rivaroxaban (aHR 0.88, 95%CI (0.80–0.97)).

### Subgroup analyses

Baseline characteristics of standard and reduced dose NOAC users before weighting are summarized in Supplemental eTable S6 and covariate balance after weighting in Supplemental eFigures S3,4. Compared to standard dose NOACs, users of reduced dose NOACs were considerably older (81.7 ± 9.0 *versus* 73.0 ± 9.3 years) and had higher CHA_2_DS_2_-VASc scores (4.1 ± 1.7 *versus* 3.2 ± 1.7), which was most pronounced in users of reduced dose apixaban (e.g., mean age of 84.1 ± 8.0 years, CHA_2_DS_2_-VASc score 4.6 ± 1.7).

The unadjusted number of events and event rates by NOAC dose are summarized in Supplemental eTable S7. Results on the effectiveness and safety of standard and reduced dose NOACs *versus* VKAs were mostly in line with the main analysis, except for standard dose rivaroxaban being associated with a significantly lower risk of myocardial infarction (aHR 0.84, 95%CI (0.72–0.97)) and reduced dose rivaroxaban being associated with significantly higher risks of MB/CRNBM (aHR 1.08, 95%CI (1.02–1.13)) and major bleeding (aHR 1.09, 95%CI (1.03–1.16)) compared to VKAs (Supplemental eTable S8). Likewise, results on the effectiveness and safety between standard dose NOACs and between reduced dose NOACs were consistent, except for a similar risk of major bleeding observed between standard dose apixaban and dabigatran, and significantly higher mortality risks observed with reduced dose apixaban compared to reduced dose dabigatran (aHR 1.40, 95%CI (1.31–1.49)), rivaroxaban (aHR 1.14, 95%CI (1.09–1.19)) and edoxaban (aHR 1.23, 95%CI (1.13–1.34)) (Supplemental eTable S9).

### Sensitivity analyses

Trends were generally consistent with 1:1 PSM (eTable 10), an intention-to-treat approach (mean follow-up of 2.6 ± 1.7 years; 659,952 person-years) (Supplemental eTable S11) and when restricting the study population to recently hospitalized OAC-naïve subjects with an ICD-coded hospital discharge diagnosis of AF (n = 125,309) (Supplemental eTable S12) or to subjects having initiated treatment between 1 October 2016 and 1 January 2019 (n = 93,542) (Supplemental eTable S3). However, no significant differences in the risk of intracranial bleeding were observed between individual NOACs and VKAs in the latter analysis. Moreover, NOACs were associated with a significantly lower risk of AF-related mortality compared to VKAs (aHR 0.76, 95%CI (0.70–0.83)), while risks were not significantly different between individual NOACs (Supplemental eTable S14). Lastly, significant interactions between apixaban and diabetes mellitus (aHR interaction term 1.19, 95%CI (1.05–1.34)) were observed for the risk of all-cause mortality compared to dabigatran, and between apixaban and digoxin use (aHR interaction term 1.34, 95%CI (1.10–1.62)) compared to edoxaban (more details provided in Supplemental Material and Supplemental eTable S15).

## Discussion

In this nationwide cohort study including more than 250,000 AF subjects between 2013 and 2019 with up to 6 years of follow-up, we have demonstrated that NOACs were associated with a superior effectiveness and non-inferior to superior safety compared to VKAs. Although the risk of thromboembolism was mostly similar between individual NOACs, potential differences in safety were identified with apixaban being associated with the most favorable safety profile across NOACs followed by dabigatran. However, the higher observed mortality risk with apixaban compared to dabigatran and edoxaban, apparently driven by patients with diabetes or using digoxin, warrants some caution and highlights the need for more long-term research.

Our results are in line with findings from randomized (Connolly et al., 2009; Granger et al., 2011; Patel et al., 2011; Giugliano et al., 2013; Ruff et al., 2014; Carnicelli et al., 2022) and observational studies (Hernandez et al., 2017; Rutherford et al., 2020; Van Ganse et al., 2020; Graham et al., 2019; Halvorsen et al., 2017; Halvorsen et al., 2021; Yao et al., 2016; Chan et al., 2018; Hohnloser et al., 2018; Larsen et al., 2016; Liu et al., 2021; Ujeyl et al., 2018; Vinogradova et al., 2018; Wong et al., 2020; Bai et al., 2017a; Ntaios et al., 2017; van den Ham et al., 2021), and corroborate guideline recommendations (Steffel et al., 2021) to prefer NOACs over VKAs in the general AF population. This is reassuring, as we have investigated unselected AF patients on a full-population scale during long-term follow-up. By including a sufficient number of edoxaban users treated in recent years, we were also able to confirm findings from the ENGAGE AF-TIMI 48 trial (Giugliano et al., 2013) by demonstrating that the risk-benefit profile of edoxaban compared to VKAs is also preserved in real-life clinical practice. To the best of our knowledge, this is one of the first nationwide cohort studies with long-term follow-up investigating the effectiveness and safety between NOAC types since the approval of edoxaban.

Despite a comparable effectiveness, we have demonstrated that dabigatran and apixaban were associated with lower risks of major bleeding compared to rivaroxaban, and that apixaban was also associated with lower major bleeding risks compared to dabigatran and edoxaban. The favorable safety profile of apixaban was driven by lower risks of upper and especially lower gastrointestinal bleeding, urogenital bleeding and/or other bleeding, except for the higher risk of other bleeding compared to dabigatran. Previous real-world observational cohort studies have also demonstrated lower risks of major bleeding with apixaban compared to rivaroxaban (Bai et al., 2017a; Hernandez et al., 2017; Cohen et al., 2018; Lee et al., 2019; Li et al., 2019; Durand et al., 2020; Rutherford et al., 2020; Van Ganse et al., 2020; Han et al., 2021; Menichelli et al., 2021; Perreault et al., 2021; Ray et al., 2021; Zhang et al., 2021; Zhu et al., 2021; Mamas et al., 2022) and dabigatran (Cohen et al., 2018; Li et al., 2019; Han et al., 2021; Menichelli et al., 2021; Zhang et al., 2021; Zhu et al., 2021), and with dabigatran compared to rivaroxaban (Bai et al., 2017b; Hernandez et al., 2017; Lee et al., 2019; Li et al., 2019; Villines et al., 2019; Durand et al., 2020; Rutherford et al., 2020; Han et al., 2021; Menichelli et al., 2021; Zhang et al., 2021; Zhu et al., 2021). These findings were also driven by a significantly lower gastrointestinal bleeding risk with apixaban compared to rivaroxaban (Hernandez et al., 2017; Cohen et al., 2018; Graham et al., 2019; Lee et al., 2019; Rutherford et al., 2020; Van Ganse et al., 2020; Han et al., 2021; Menichelli et al., 2021; Perreault et al., 2021; Ray et al., 2021; Zhang et al., 2021; Zhu et al., 2021; Mamas et al., 2022) and dabigatran (Cohen et al., 2018; Graham et al., 2019; Lee et al., 2019; Villines et al., 2019; Rutherford et al., 2020; Van Ganse et al., 2020; Han et al., 2021; Menichelli et al., 2021; Zhang et al., 2021; Zhu et al., 2021), and a similar (Villines et al., 2019) to lower (Bai et al., 2017b; Hernandez et al., 2017; Lai et al., 2017; Graham et al., 2019; Lee et al., 2019; Li et al., 2019; Menichelli et al., 2021; Zhang et al., 2021; Zhu et al., 2021) gastrointestinal bleeding risk with dabigatran compared to rivaroxaban. However, some studies did not observe significant differences in major bleeding between apixaban and dabigatran (Bai et al., 2017a; Hernandez et al., 2017; Durand et al., 2020; Rutherford et al., 2020; Van Ganse et al., 2020). Although our results should be considered as hypothesis-generating and interpreted with caution given the observational nature of our data, these findings may help clinicians in choosing an anticoagulant in clinical practice while awaiting further evidence.

In line with results from RCTs (Connolly et al., 2009; Granger et al., 2011; Patel et al., 2011; Giugliano et al., 2013; Ruff et al., 2014; Carnicelli et al., 2022) and observational studies (Hernandez et al., 2017; Van Ganse et al., 2020; Graham et al., 2019; Halvorsen et al., 2021; Yao et al., 2016; Hohnloser et al., 2018; Larsen et al., 2016; Liu et al., 2021; Ujeyl et al., 2018; Vinogradova et al., 2018; Wong et al., 2020; Bai et al., 2017a; Ntaios et al., 2017; van den Ham et al., 2021; Bai et al., 2017b; Forslund et al., 2018), the risk of intracranial bleeding, the most feared and frequently fatal complication of anticoagulation, was significantly lower with NOACs compared to VKAs (Ruff et al., 2014). No differences between individual NOACs on the risk of intracranial bleeding were observed, except for a significantly lower risk with apixaban compared to rivaroxaban, which has been noted before (Cohen et al., 2018; Rutherford et al., 2020; Zhang et al., 2021; Zhu et al., 2021). However, the non-significantly lower risk of intracranial bleeding with edoxaban compared to VKAs may be due to the short follow-up duration and insufficient number of events among edoxaban users, given that edoxaban has only been approved in Belgium since October 2016 and intracranial bleeding is a rare complication of anticoagulation. Exemplary, when analyses were restricted to the subgroup of patients having initiated therapy from October 2016 onwards, the risk of intracranial bleeding was no longer significantly lower with dabigatran, apixaban and rivaroxaban compared to VKAs as well. Likewise, other studies conducted shortly after the approval of apixaban could also not illustrate a significantly lower risk of intracranial bleeding with apixaban compared to VKAs (Larsen et al., 2016; Ujeyl et al., 2018).

Identified as a research gap in real-world data (Cohen et al., 2018), we have demonstrated that the risk of urogenital bleeding was not significantly different between NOACs and VKAs, except for a significantly higher risk with rivaroxaban compared to VKAs and other NOACs, and a lower risk with apixaban compared to edoxaban. One other observational study also assessed urogenital bleeding risks and observed similar trends, namely that rivaroxaban was associated with an increased risk of hematuria compared to apixaban (Vinogradova et al., 2018). Future real-world studies are needed to confirm these findings.

Despite early reports of potentially increased risks of myocardial infarction with dabigatran (Connolly et al., 2009), we did not observe significant differences in the risk of myocardial infarction between individual NOACs and VKAs or between NOAC types. Likewise, other observational studies could also not replicate these early findings and highlighted comparable risks of myocardial infarction between anticoagulants (Bai et al., 2017b; Lai et al., 2017; Ntaios et al., 2017; Li et al., 2019; Durand et al., 2020; Menichelli et al., 2021). In contrast, a significantly lower risk of myocardial infarction was observed with standard dose rivaroxaban compared to VKAs, which is of interest. Future studies are needed to elaborate on the role of rivaroxaban and other NOACs for (secondary) prevention of vascular events in AF patients at high atherothrombotic risk.

Remarkably, the risk of all-cause mortality was significantly higher with apixaban compared to dabigatran and edoxaban, especially in subjects using reduced dose apixaban. Despite additional adjustment for potential residual confounding in DRE models, the risk of death associated with apixaban was still significantly increased in subjects having diabetes or using digoxin. It should be noted that thromboembolic and intracranial bleeding risks were however similar and major and gastrointestinal bleeding risks were significantly lower with apixaban compared to other NOACs, which may indicate that the higher mortality risks in apixaban users were driven by higher risks of non-AF-related death. Exemplary, no significant differences in the risk of AF-related mortality, defined as deaths occurring within 60 days after an event of thromboembolism, bleeding or myocardial infarction, were observed between individual NOACs. Moreover, it remains important to note that (reduced dose) apixaban users were older, had more comorbidities and more polypharmacy than other NOAC users, which may indicate selective prescribing of apixaban to the oldest and sickest AF patients (e.g., due to pre-existing physician-perceived differences in safety between NOACs). Although we minimized confounding by indication using IPTW, we cannot exclude any influence of unmeasured confounding or selective prescribing, since vulnerable geriatric traits in older AF patients, such as underweight, sarcopenia, renal dysfunction or general poor health are difficult to capture in administrative healthcare databases, given the lack of data on body weight, muscle strength, creatinine clearance and disease severity (Chan et al., 2018; Forslund et al., 2018; Ujeyl et al., 2018; Halvorsen et al., 2021; Zhang et al., 2021). More studies are needed to replicate these exploratory findings, especially in the subgroup of subjects with diabetes or using digoxin, including cause-specific mortality.

### Strengths and limitations

Strengths of this first nationwide cohort study on AF-related anticoagulant use in Belgium include the long-term follow-up up to 6 years for a total of 328,796 person-years, inclusion of unselected AF patients on a full-population scale including many edoxaban users and difficult-to-reach subgroups to eliminate selection bias, and assessment of anticoagulant dispensing in ambulatory and hospital care. However, several limitations should be mentioned. First, coding errors and misclassification bias may be present due to our observational design using healthcare databases, for which regular assessments of the proportion of potential coding errors could further improve quality. However, by identifying comorbidities based on ICD, medical procedure codes and/or drug prescription claims assessed in ambulatory and hospital care, missing data and misclassification of characteristics were reduced. Second, although we thoroughly adjusted for confounders using IPTW, there is a risk of unmeasured confounding due to missing lifestyle characteristics (e.g., weight, smoking) and laboratory values (e.g., renal function, INR, hemoglobin levels). In line, (in)appropriate NOAC dosing and time in therapeutic range of VKA users could not be assessed. Third, although patients with competing treatment indications were excluded (e.g., pulmonary embolism), subjects were not required to have an ICD-coded hospital discharge diagnosis of AF to be included (to reduce selection bias), as this would have limited the study population to recently hospitalized AF patients and excluded AF patients treated exclusively in primary or ambulatory care (Hellfritzsch et al., 2020). Nevertheless, trends were consistent when specifically investigating subjects with an ICD-coded diagnosis of AF (to reduce misclassification bias). Fourth, the follow-up duration of edoxaban users was considerably shorter than other NOACs due to variable approval dates. Nevertheless, results were broadly consistent in a sensitivity analysis restricting the study population to subjects having initiated treatment since October 2016. Fifth, although the risk of AF-related mortality was explored, data were lacking on the exact causes of death, which would have been of interest to explore why differences in the risk of all-cause mortality between individual NOACs were observed. Sixth, anticoagulant use was assessed based on dispensing data to account for discontinuation or switch of treatment, not on the patients’ actual intake or physicians’ prescriptions. In line, the impact of therapy adherence on the benefit-risk profile of OACs was not considered. Nevertheless, findings were consistent using an intention-to-treat approach. Lastly, AF patients using free (NOAC) drug samples were not identified and data of over-the-counter or non-reimbursed drug use were not available.

## Conclusion

In conclusion, NOACs were associated with a superior long-term effectiveness and non-inferior to superior safety compared to VKAs in an unselected real-world population with AF. Although effectiveness was comparable between individual NOACs, safety outcomes differed with apixaban being associated with the most favorable safety profile across NOACs followed by dabigatran, driven by lower risks of upper and especially lower gastrointestinal, urogenital and/or other bleeding. However, the potentially increased mortality risk with apixaban compared to dabigatran and edoxaban warrants some caution, while awaiting further research.

## Data Availability

The datasets presented in this article are not readily available because Requests for the data underlying this article should be directed to the administrators of the InterMutualistic Agency (IMA) database or Minimal Hospital Dataset and is subject to approval. Requests to access the datasets should be directed to https://ima-aim.be/and https://www.health.belgium.be/en/node/23607.
