# Supplementary material for: Long-term comparative effectiveness and safety of dabigatran, rivaroxaban, apixaban and edoxaban in patients with atrial fibrillation: A nationwide cohort study
Source: Front Pharmacol. 2023 Feb 2;14:1125576. doi: 10.3389/fphar.2023.1125576 (PMC9932194; doi:10.3389/fphar.2023.1125576)
Supplement: Supplementary file 1 [file Table1.DOCX]

**Supplementary materials**

Table of contents

[Supplemental methods 4](#_Toc124947003)

[Source population 4](#_Toc124947004)

[Follow-up 4](#_Toc124947005)

[Covariates 5](#_Toc124947006)

[Supplemental results 6](#_Toc124947007)

[Sensitivity analyses 6](#_Toc124947008)

[Supplemental tables 7](#_Toc124947009)

[eTable 1: STROBE reporting guideline 7](#_Toc124947010)

[eTable 2: Definition of in- and exclusion criteria, comorbidities, medication history and clinical risk scores 9](#_Toc124947011)

[eTable 3: Definition of outcomes 17](#_Toc124947012)

[eTable 4: Adjusted HRs of outcomes compared between NOACs and VKAs after IPTW (main analysis) 19](#_Toc124947013)

[eTable 5: Adjusted HRs of outcomes compared between individual NOACs after IPTW (main analysis) 20](#_Toc124947014)

[eTable 6: Baseline characteristics of standard and reduced dose NOACs versus VKAs 21](#_Toc124947015)

[eTable 7: Number of events and crude event rates of outcomes with standard and reduced dose NOACs 23](#_Toc124947016)

[eTable 8: Adjusted HRs of outcomes compared between standard or reduced dose NOACs and VKAs after IPTW 24](#_Toc124947017)

[eTable 9: Adjusted HRs of outcomes compared between individual standard or reduced dose NOAC types after IPTW 26](#_Toc124947018)

[eTable 10: Adjusted HRs of outcomes after 1:1 PSM 28](#_Toc124947019)

[eTable 11: Adjusted HRs of outcomes after IPTW (intention-to-treat analysis) 30](#_Toc124947020)

[eTable 12: Adjusted HRs of outcomes after IPTW (patients with ICD-coded hospital discharge diagnosis of AF) 32](#_Toc124947021)

[eTable 13: Adjusted HRs of outcomes after IPTW (October 1^st^, 2016 – January 1^st^, 2019) 34](#_Toc124947022)

[eTable 14: The risk of AF-related mortality 36](#_Toc124947023)

[eTable 15: Significant interactions between treatment and covariates regarding the risk of mortality between individual NOACs using doubly robust estimation models 37](#_Toc124947024)

[Supplemental figures 39](#_Toc124947025)

[eFigure 1: Overview of study design 39](#_Toc124947026)

[eFigure 2: Love plots (main analysis) 40](#_Toc124947027)

[eFigure 3: Love plots (standard dose NOACs) 46](#_Toc124947028)

[eFigure 4: Love plots (reduced dose NOACs) 52](#_Toc124947029)

[References 58](#_Toc124947030)

# Supplemental methods

## Source population

Two nationwide databases provided the source population, namely the InterMutualistic Agency (IMA) database and Minimal Hospital Dataset (MHD) , as described before.^1^ The IMA centralizes all claims data from Belgian health insurance funds on reimbursed ambulatory and hospital care, including demographic characteristics (e.g. age, sex), medical procedures (diagnostic or therapeutic procedures and other reimbursed care) and drug prescription claims.^2,3^ Since health insurance is legally mandatory in Belgium, the source population represents all legal residents with reimbursed medication or care. The MHD is managed by the Belgian Ministry of Health and aggregates hospital discharge diagnoses of every hospital admission (including hospitalizations, day-care stays and emergency room contacts), coded in International Classification of Diseases (ICD) codes (ICD-9 up to 2014, ICD-10 from 2015 onwards).^2,4^ Both databases were deterministically linked by the Trusted Third Party ‘eHealth’ using the national social security number as unique patient identifier. Only pseudonymized data were available to the researchers on the secured IMA servers after applying an encrypting procedure for privacy protection. This study was approved by the IMA and MHD database administrators and by the ‘Sectoral Committee of Social Security and Health, Section Health’, a subcommittee of the Belgian Commission for the Protection of Privacy (approval code IVC/KSZG/20/344), waiving the need for individual informed consents.^5^

## Follow-up

Patients were followed from oral anticoagulant (OAC) initiation (=index date) until occurrence of the investigated outcome, discontinuation or switch of treatment, death, emigration or end of the study period (January 1^st^, 2019), whichever came first (per-protocol analysis). Discontinuation was defined using a >60-day supply gap after the calculated last day of supply, with the possibility to extend this gap for vitamin K antagonists (VKAs) in case of intervening INR testing at least every 42 days.^6-9^ This approach has shown to substantially reduce misclassification of VKA discontinuation^7,8^ and accounts for delays in refilling, minor non-adherence, variable VKA dosing regimens and residual drug effects.^8-12^ The date of discontinuation was defined as the calculated last day of supply (or last INR test date if VKA-treated, whichever came last).^6-9^ INR test dates were identified using medical procedure codes (eTable 3). The last day of supply was calculated based on ambulatory and hospital prescription claims data (dispensing date, tablet strength, package size and number of packages supplied) and the recommended dosing regimen (twice-daily for dabigatran and apixaban; once-daily for rivaroxaban and edoxaban; defined daily dose (DDD) of 7.5 mg for warfarin, 5 mg for acenocoumarol and 3 mg for phenprocoumon^13^). In case of overlap between two consecutive refills of the same OAC, the start date of the new dispensing was shifted to the day after the last day of supply of the prior dispensing.^14^ Stockpiling was allowed up to 180 days of accumulated days of supply.^15^ Switching was defined as the dispensing of another OAC before or within 60 days after the last day of supply of the index OAC, considering the dispensing date of the new OAC as the switch date.

## Covariates

Age, sex, comorbidities, medication history and clinical risk scores were assessed at baseline. Comorbidities were identified using specific ICD-coded (International Classification of Diseases) diagnoses (e.g. cancer), medical procedure codes (e.g. cancer-related surgery) and/or ATC-coded (Anatomical Therapeutic Chemical) prescription claims (e.g. antineoplastic drugs) ≤1 year before the index date (eTable 1). Comorbidities included hypertension, heart failure, coronary and peripheral artery disease, valvular heart disease (aortic, mitral, pulmonary or tricuspid valve disease, valve repair or bioprosthetic heart valve replacement), dyslipidemia, chronic kidney disease (CKD stage III-IV or renal transplant), chronic liver disease (including liver cirrhosis or liver transplant), chronic lung disease (including COPD, asthma or interstitial lung disease), obstructive sleep apnea, cancer, upper and lower gastrointestinal tract disorders (gastroesophageal reflux disease or peptic ulcer disease; and diverticulosis, angiodysplasia, colorectal polyposis or hemorrhoids, respectively), diabetes mellitus, anemia, dementia, history of falling, frailty (using the Claims-based Frailty Indicator^16^), prior thromboembolism (stroke or systemic embolism) and prior major or clinically relevant non-major bleeding (intracranial, gastrointestinal, urogenital or other bleeding event necessitating hospitalization).

Medication history was identified with ATC-coded prescription claims, considering recent use ≤6 months before the index date. Based on the total number of concomitant drugs, polypharmacy and hyperpolypharmacy were defined as the dispensing of 5-9 and ≥10 concomitant drugs during ≥30 days (consecutive or not) in the last 6 months before the index date, respectively.^17^

Lastly, the CHA_2_DS_2_-VASc score, HAS-BLED score and age-adjusted Charlson Comorbidity Index were calculated.^18^ Since only INR test dates but not values were available, a modified HAS-BLED score was used without the ‘labile INR’ criterion. Alcohol abuse was indirectly identified with ICD (e.g. alcoholic liver disease), ATC (e.g. disulfiram) or medical procedure codes (e.g. visit to psychologist for alcohol abuse).

# Supplemental results

## Sensitivity analyses

Lastly, significant interactions between apixaban and diabetes mellitus (aHR interaction term 1.19, 95%CI (1.05-1.34), p-value 0.005) were observed after weighting and multivariable adjustment in exploratory DRE models for the risk of all-cause mortality compared to dabigatran, and between apixaban and the use of digoxin (aHR interaction term 1.34, 95%CI (1.10-1.62), p-value 0.003) compared to edoxaban, while the use of corticosteroids was associated with a significantly lower all-cause mortality risk (aHR interaction term 0.85, 95%CI (0.74-0.99), p-value 0.034) (eTable 15). Significant interactions with the risk of all-cause mortality and dabigatran, apixaban and edoxaban compared to rivaroxaban were observed in patients with lower gastrointestinal tract disorders. Edoxaban was also associated with lower mortality risks in patients with chronic liver disease (aHR interaction term 0.69, 95%CI (0.50-0.95), p-value 0.024) compared to rivaroxaban (eTable 15).

# Supplemental tables

## eTable 1: STROBE reporting guideline

|  | **Item No.** | **Recommendation** | **Page No.** |
| --- | --- | --- | --- |
| **Title and abstract** | 1 | (*a*) Indicate the study’s design with a commonly used term in the title or the abstract | 1-2 |
|  |  | (*b*) Provide in the abstract an informative and balanced summary of what was done and what was found | 2 |
| **Introduction** | | |  |
| Background/rationale | 2 | Explain the scientific background and rationale for the investigation being reported | 3 |
| Objectives | 3 | State specific objectives, including any prespecified hypotheses | 4 |
| **Methods** | | |  |
| Study design | 4 | Present key elements of study design early in the paper | 5 |
| Setting | 5 | Describe the setting, locations, and relevant dates, including periods of recruitment, exposure, follow-up, and data collection | 5-7 |
| Participants | 6 | (*a*) Give the eligibility criteria, and the sources and methods of selection of participants. Describe methods of follow-up | 5-7 |
|  |  | (*b*) For matched studies, give matching criteria and number of exposed and unexposed | 7-8 |
| Variables | 7 | Clearly define all outcomes, exposures, predictors, potential confounders, and effect modifiers. Give diagnostic criteria, if applicable | 6-7, eTable 2-3 |
| Data sources/ measurement | 8* | For each variable of interest, give sources of data and details of methods of assessment (measurement). Describe comparability of assessment methods if there is more than one group | 5-7 |
| Bias | 9 | Describe any efforts to address potential sources of bias | 7-9 |
| Study size | 10 | Explain how the study size was arrived at | 5-6 |
| Quantitative variables | 11 | Explain how quantitative variables were handled in the analyses. If applicable, describe which groupings were chosen and why | 7-8 |
| Statistical methods | 12 | (*a*) Describe all statistical methods, including those used to control for confounding | 7-8 |
|  |  | (*b*) Describe any methods used to examine subgroups and interactions | 8-9 |
|  |  | (*c*) Explain how missing data were addressed | 7 |
|  |  | (*d*) If applicable, explain how loss to follow-up was addressed | / |
|  |  | (*e*) Describe any sensitivity analyses | 9 |
| **Results** | | |  |
| Participants | 13* | (a) Report numbers of individuals at each stage of study—eg numbers potentially eligible, examined for eligibility, confirmed eligible, included in the study, completing follow-up, and analysed | 10, Table 1 |
|  |  | (b) Give reasons for non-participation at each stage | / |
|  |  | (c) Consider use of a flow diagram | Figure 1 |
| Descriptive data | 14* | (a) Give characteristics of study participants (eg demographic, clinical, social) and information on exposures and potential confounders | Table 1 |
|  |  | (b) Indicate number of participants with missing data for each variable of interest | Table 1 |
|  |  | (c) Summarise follow-up time (eg, average and total amount) | Table 1 |
| Outcome data | 15* | Report numbers of outcome events or summary measures over time | 10-14, Table 2 |
| Main results | 16 | (*a*) Give unadjusted estimates and, if applicable, confounder-adjusted estimates and their precision (eg, 95% confidence interval). Make clear which confounders were adjusted for and why they were included | 10-14, Table 3-4 |
|  |  | (*b*) Report category boundaries when continuous variables were categorized | 10-14, Table 1 |
|  |  | (*c*) If relevant, consider translating estimates of relative risk into absolute risk for a meaningful time period | / |
| Other analyses | 17 | Report other analyses done—eg analyses of subgroups and interactions, and sensitivity analyses | 12-14, eTable 6-12 |
| **Discussion** | | |  |
| Key results | 18 | Summarise key results with reference to study objectives | 15-17 |
| Limitations | 19 | Discuss limitations of the study, taking into account sources of potential bias or imprecision. Discuss both direction and magnitude of any potential bias | 17-18 |
| Interpretation | 20 | Give a cautious overall interpretation of results considering objectives, limitations, multiplicity of analyses, results from similar studies, and other relevant evidence | 15-17 |
| Generalisability | 21 | Discuss the generalisability (external validity) of the study results | 15-17 |
| **Other information** | | |  |
| Funding | 22 | Give the source of funding and the role of the funders for the present study and, if applicable, for the original study on which the present article is based | 20 |

*Give information separately for exposed and unexposed groups.

**eTable 1:** Compliance to the STROBE (Strengthening the Reporting of Observational Studies in Epidemiology) reporting guideline.^19^

## eTable 2: Definition of in- and exclusion criteria, comorbidities, medication history and clinical risk scores

| **VARIABLE** | | **ICD, ATC AND MEDICAL PROCEDURE CODES** |
| --- | --- | --- |
| **INCLUSION CRITERIA** | | |
| OAC use (NOAC or VKA) | | **ATC:** B01AE07, B01AF01, B01AF02, B01AF03, B01AA03, B01AA04, B01AA07 |
| ≥45 years | | Age ≥45 years on index date |
| ≥1 year coverage | | ≥1 year coverage by a Belgian health insurance fund |
| Atrial fibrillation (sensitivity analysis) | | **ICD 9:** 427.3  **ICD 10:** I48 |
| **EXCLUSION CRITERIA** | | |
| Recent total hip/knee replacement surgery | | **Medical procedure group code:** N32 (≤6 months before index date) |
| Recent venous thromboembolism  (deep vein thrombosis or pulmonary embolism) | | **ICD-9:** 451.1, 451.2, 451.81, 451.89, 451.9, 452, 453.2, 453.3, 453.4, 453.5, 453.77, 453.79, 453.87, 453.89, 671.3, 671.4 (≤6 months before index date)  **ICD-10:** I80.1, I80.2, I80.3, I80.8, I80.9, I81, I82.2, I82.3, I82.4, I82.5, I82.89, I82.9, O22.3, O22.5, O87.1, O87.3 (≤6 months before index date) |
| Valvular atrial fibrillation | |  |
|  | Moderate-severe mitral stenosis | **ICD-9:** 394.0, 394.2, 396.0, 396.1, 746.5  **ICD-10:** I05.0, I05.2, I34.2, Q23.2 |
|  | Mechanical prosthetic heart valve | **ICD-9:** V43.3  **ICD-10:** Z95.2  **Medical procedure code:** 159110, 159121, 159132, 159143, 159154, 159165 |
| End-stage renal disease | |  |
|  | CKD stage V (without dialysis) | **ICD-9:** 403.01, 403.11, 403.91, 404.02, 404.12, 404.92, 585.5, 585.6, 586  **ICD-10:** N18.5, N18.6, N19, I12.0, I13.11 |
|  | Dialysis | **ICD-9:** V45.11, V56  **ICD-10:** Z49, Z99.2  **Medical procedure group code:** N81 |
| **DEMOGRAFICS (on index date)** | | |
| Age | | Age on index date based on the year and month of birth, not the exact date due to patient privacy. |
| Sex | | Sex on index date |
| **COMORBIDITIES (≤1 year before index date)**^13,20,21^ | | |
| Hypertension | | **ICD-9:** 401-405, 437.2  **ICD-10:** I10-I13, I15, I16, I67.4  **ATC:** combination treatment with ≥2 of the following drug classes:  **I) Cardioselective beta blocker:** C07AB, C07AG, C07BB, C07BG, C07CB, C07CG, C07DB, C07FB, C07FX03, C07FX04, C07FX05, C07FX06, C09BX02, C09BX04, C09BX05, C09DX05  **II) ACE inhibitor or angiotensin II receptor blocker:** C09A, C09B, C09C, C09D, C10BX04, C10BX06, C10BX07, C10BX10, C10BX11, C10BX12, C10BX13, C10BX14, C10BX15, C10BX16, C10BX17, C10BX18  **III) Calcium channel blocker:** C07FB, C08C, C08G, C09BB, C09DB, C09BX01, C09BX03, C09BX04, C09DX01, C09DX03, C09DX06, C09DX07, C09XA53, C09XA54, C10BX03, C10BX07, C10BX09, C10BX11, C10BX14, C10BX18  **IV) Non-loop diuretic:** C02L, C03A, C03BA, C03BB, C03EA, C07B, C07C, C07D, C08GA, C09BA, C09BX01, C09BX03, C09DA, C09DX01, C09DX03, C09DX06, C09DX07, C09XA52, C09XA54, C10BX13, C03D, C03EA, C03EB  **V) Other antihypertensive (alpha adrenergic blocker, vasodilator)**: C02A, C02B, C02C, C02DB, C02DD, C02DG, C02L |
| Coronary artery disease  (stable or recent myocardial infarction) | | **ICD-9:** 410, 411, 412, 413, 414, 429.2, 429.7, V45.81, V45.82  **ICD-10:** I20, I21, I22, I23, I24, I25, Z95.1, Z95.5, Z98.61  **Medical procedure code:** 158992, 159003, 159014, 159025, 159036, 159040, 229515, 229526, 229574, 229585, 229611, 229622, 229633, 229644, 589013, 589024, 589153, 589164, 589934, 589945, 589956, 589960, 680315, 680326, 680352, 680363, 687875, 687886 |
| Congestive heart failure | | **ICD-9:** 398.91, 402.01, 402.11, 402.91, 404.01, 404.03, 404.11, 404.13, 404.91, 404.93, 425.4–425.9, 428  **ICD-10:** I09.81, I11.0, I13.0, I13.2, I42.0, I42.6-I42.9, I43, I50  **ATC:** combination treatment of all of the following drug classes:  **I) Cardioselective beta blocker:** C07AB, C07AG, C07BB, C07BG, C07CB, C07CG, C07DB, C07FB, C07FX03, C07FX04, C07FX05, C07FX06, C09BX02, C09BX04, C09BX05, C09DX05  **II) ACE-inhibitor or ARB:** C09A, C09B, C09C, C09D, C10BX04, C10BX06, C10BX07, C10BX10, C10BX11, C10BX12, C10BX13, C10BX14, C10BX15, C10BX16, C10BX17, C10BX18  **III) Potassium-sparing diuretic:** C03D, C03EA, C03EB  **IV) Loop diuretic:** C03C, C03EB |
| Valvular heart disease  (aortic, mitral or other/non-specific valve disease, valve repair, bioprosthetic heart valve) *(except for moderate-severe mitral stenosis or mechanical prosthetic heart valve)* | | **ICD 9:** 036.42, 074.22, 093.2, 098.84, 112.81, 391.1, 394.1, 394.2, 394.9, 395.0, 395.1, 395.2, 395.9, 396, 397, 421, 424.0, 424.1, 424.2, 424.3, 424.9, 746.0, 746.1, 746.3, 746.4, 746.6, V42.2  **ICD 10:** A32.82, A39.51, A52.03, A54.83, B33.21, B37.6, I01.1, I05.1, I05.2, I05.8, I05.9, I06.0, I06.1, I06.2, I06.8, I06.9, I07, I08, I09.1, I09.89, I33, I34.0, I34.1, I34.8, I34.9, I35.0, I35.1, I35.2, I35.8, I35.9, I36, I37, I38, I39, M32.11, Q22, Q23.0, Q23.1, Q23.3, Q23.8, Q23.9, Z95.3, Z95.4  **Medical procedure code:** 159176, 159180, 159191, 159202, 159213, 159224, 159235, 159246, 159250, 159261, 159272, 159283, 159294, 159305, 170634, 170645, 172491, 172502, 172513, 172524, 172734, 172745, 172756, 172760, 172771, 172782, 172955, 172966, 172970, 172981, 172992, 173003, 229515, 229526, 229596, 229600, 589190, 589201, 680153, 680164, 680175, 680186, 680993, 681004, 684736, 684740, 688192, 688203, 691950, 691961, 704616, 704620, 704631, 704642, 704653, 704664 |
| Peripheral artery disease | | **ICD-9:** 440, 441, 443.89, 443.9, 444, 447.1, 557.1, 557.9, V43.4  **ICD-10:** I70, I71, I73.8, I73.9, I74, I77.1, I79.0, K55.1, K55.8, K55.9, Z95.82, Z98.62  **Medical procedure code:** 229294, 229305, 229316, 229320, 229331, 229342, 235071, 235082, 235093, 235104, 235115, 235126, 235196, 235200, 235211, 235222, 236014, 236025, 236036, 236040, 236051, 236062, 237016, 237020, 237031, 237042, 237053, 237064, 237075, 237086, 237090, 237101, 237171, 237182, 589050, 589061, 589094, 589105, 589175, 589186, 589595, 589606, 589610, 589621, 589632, 589643, 589654, 589665 |
| Dyslipidemia | | **ICD-9:** 272.0, 272.1, 272.3, 272.4, 272.5, 272.8, 272.9  **ICD-10:** E78  **ATC:** C10 |
| Chronic kidney disease  (CKD stage III-IV, renal transplant, other/non-specific CKD) *(except for CKD stage V or dialysis)* | | **ICD-9:** 249.4, 250.4, 403.00, 403.10, 403.90, 404.00, 404.10, 404.90, 580, 581, 582, 583, 584, 585.3, 585.4, 585.9, 586, 588, 590.0, 753.12-753.15, 996.81, V42.0  **ICD-10:** E08.2, E09.2, E10.2, E11.2, E13.2, I12.9, I13.10, M32.14, M32.15, N00, N01, N02, N03, N04, N05, N07, N11, N14, N15.0, N17, N18.3, N18.4, N18.9, N19, N25, Q61.1-Q61.4, T86.1, Z94.0  **Medical procedure code:** 107096, 107111, 107133, 107155, 318010, 318021, 318290, 318301, 754294, 757433, 757492 |
| Chronic liver disease  (mild, moderate-severe, cirrhosis) | | **ICD-9:** 070.0, 070.2, 070.3, 070.4, 070.51, 070.52, 070.54, 070.6, 070.70, 070.71, 155.0, 155.1, 155.2, 197.7, 456.0, 456.1, 456.2, 567.23, 570, 571, 571.2, 571.5, 571.6, 572.2, 572.3, 572.4, 572.8, 573.0, 573.5, 573.8, 573.9, 789.59, V42.7  **ICD-10:** B15.0, B16.0, B16.2, B17.0, B17.10, B17.11, B18, B19.0, B19.10, B19.11, B19.20, B19.21, C22, C78.7, I85, I86.4, K65.2, K70.0, K70.1, K70.2, K70.3, K70.4, K70.9, K71.1, K71.3, K71.4, K71.5, K71.6, K71.7, K71.8, K71.9, K72, K73, K74, K75.3, K75. 4, K75.8, K75.9, K76.0, K76.1, K76.2, K76.5, K76.6, K76.7, K76.81, K76.89, K76.9, K77, R18.8, Z94.4  **ATC:** J05AB04, J05AF05, J05AF07, J05AF08, J05AF10, J05AE11, J05AE12, J05AE14, J05AX15, J05AX65, J05AP  **Medical procedure code:** 318076, 318080, 318334, 318345, 472113, 472124, 556754, 556765, 589352, 589363 |
| Chronic lung disease  (COPD, asthma, other) | | **ICD-9**: 416, 491-496, 500-505, 506.4, 508.1, 515, 516.3, 516.9, 518.1, 518.2, 518.83, 518.84  **ICD-10**: I27, J41-J45, J47, J60-J67, J68.4, J70.1, J70.3, J84.1, J84.9, J96.1, J96.2, J98.2, J98.3  **ATC:** R03DC, R03DX |
| Obstructive sleep apnea | | **ICD-9:** 327.23  **ICD-10**: G47.33  **Medical procedure code:** 765951, 779870, 779881, 779892, 779903, 779914, 779925, 779936, 779951, 788012, 788023 |
| Cancer | | **ICD-9:** 140-209, 223, 230-239, 258.0, V58.0, V58.11, V58.12  **ICD-10:** C00-C96, D00-D09, D37-D49, E31.2, Z51.0, Z51.11, Z51.12  **ATC:** L01  **Medical procedure code:** 154873, 154884, 154895, 154906, 157231, 157242, 201191, 201202, 201213, 201224, 220275, 220286, 220371, 220382, 201213, 201224, 226914, 226925, 226936, 226940, 227216, 227220, 227275, 227286, 227636, 227640, 227651, 227662, 227673, 227684, 227695, 227706, 227710, 227721, 227732, 227743, 227754, 227765, 227776, 227780, 227791, 227802, 227813, 227824, 227835, 227846, 228012, 228023, 228174, 228185, 228233, 228244, 228255, 228266, 228270, 228281, 228292, 228303, 228314, 228325, 228336, 228340, 230473, 230484, 231033, 231044, 241231, 241242, 241415, 241426, 241430, 241441, 241452, 241463, 241555, 241566, 242012, 242023, 242034, 242045, 242292, 242303, 242314, 242325, 242830, 242841, 242852, 242863, 242874, 242885, 242896, 242900, 243051, 243062, 243073, 243084, 243235, 243246, 243736, 243740, 243751, 243762, 243773, 243784, 244016, 244020, 244031, 244042, 244075, 244086, 244790, 244801, 244856, 244860, 244893, 244904, 244915, 244926, 244930, 244941, 244952, 244963, 244974, 244985, 245512, 245523, 245534, 245545, 246050, 246061, 246072, 246083, 247111, 247122, 247133, 247144, 251753, 251764, 251775, 251786, 254892, 254903, 256115, 256126, 256336, 256340, 256572, 256583, 257191, 257202, 258355, 258366, 258370, 258381, 258392, 258403, 258451, 258462, 258554, 258565, 258856, 258860, 258871, 258882, 258893, 258904, 259033, 259044, 259114, 259125, 260190, 260201, 260411, 260422, 260433, 260444, 260551, 260562, 260654, 260665, 260750, 260761, 261111, 261122, 261391, 261402, 261472, 261483, 261671, 261682, 261774, 261785, 261796, 261800, 262334, 262345, 262570, 262581, 277756, 277760, 277771, 277782, 278795, 278806, 278810, 278821, 281831, 281842, 281956, 281960, 282310, 282321, 282671, 282682, 284056, 284060, 288455, 288466, 288470, 288481, 289892, 289903, 291056, 291060, 310494, 310505, 311312, 311323, 312550, 312561, 312572, 312583, 312594, 312605, 312653, 312664, 312970, 312981, 350114, 350125, 350136, 350140, 350276, 350280, 350291, 350302, 350372, 350383, 350674, 350685, 350696, 350700, 431174, 431185, 431336, 431340, 431351, 431362, 432294, 432305, 444113, 444124, 444135, 444146, 444150, 444161, 444172, 444183, 444194, 444205, 444216, 444220, 444231, 444242, 444253, 444264, 444275, 444286, 444290, 444301, 444312, 444323, 444334, 444345, 444474, 444485, 444592, 444603, 473970, 473981, 474795, 474806, 565073, 565084, 565095, 565106, 565110, 565121, 565132, 565143, 565154, 565165, 587834, 587845, 587871, 587882, 587893, 587904, 587915, 587926, 588431, 588442, 588453, 588464, 588475, 588486, 588490, 588501, 588512, 588523, 588534, 588545, 588556, 588560, 588571, 588582, 588593, 588604, 588770, 588781, 588976, 588980, 589691, 589702, 589713, 589724, 589831, 589842, 589875, 589886, 594016, 594020, 594031, 594042, 594053, 594064, 594075, 594086, 594090, 594101, 594112, 594123, 594252, 594263, 594274, 594285, 594296, 594300, 594311, 594322, 594333, 594344, 594355, 594366, 594370, 594381, 594392, 594403, 594414, 594425, 594436, 594440, 594451, 594462, 594495, 594506, 594510, 594521, 594532, 594543, 594554, 594565, 594576, 594580, 594591, 594602, 594613, 594624, 594635, 594646, 594694, 594705, 594716, 594720, 594753, 594764, 594775, 594786, 594790, 594801, 594812, 594823, 594834, 594845, 594856, 594860, 594871, 594882, 594893, 594904, 594915, 594926, 594930, 594941, 598581, 682636, 682640, 682732, 682743, 687934, 687945, 698051, 698062, 698095, 698106, 698390, 698401, 698456, 698460, 698471, 698482, 698493, 698504, 698530, 698541, 745010, 745021, 745032, 745043, 745113, 745124, 745135, 745146, 745150, 745161 |
| Upper gastrointestinal tract disorder (gastroesophageal reflux disease or peptic ulcer disease) | | **ICD-9:** 041.86, 530.1, 530.2, 530.81, 530.85, 531, 532, 533, 534, 535, V12.71  **ICD-10:** B96.81, K20, K21, K22.1, K22.7, K25, K26, K27, K28, K29, Z87.11  **ATC:** A02BD04, A02BD11  **Medical procedure code:** 172616, 172620, 172631, 172642, 172653, 172664, 474854, 474865, 550093, 550104, 552370, 552381 |
| Lower gastrointestinal tract disorder (polyposis, diverticulosis, angiodysplasia, hemorrhoids) | | **ICD-9:** 211.3, 211.4, 448.0, 455, 537.82, 537.83, 537.84, 562, 569.84, 569.85, 569.86, V12.72  **ICD-10:** D12, I78.0, K31.81, K31.82, K55.2, K57, K63.5, K64, K63.81, Z86.010  **Medical procedure code:** 112313, 112324, 243294, 243305, 244311, 244322, 244355, 244366, 244370, 244381, 244554, 244565, 244576, 244580, 244591, 244602, 472150, 472161, 473211, 473222, 473476, 473480, 473675, 473686, 473955, 473966, 473970, 473981, 474795, 474806 |
| Diabetes mellitus | | **ICD-9:** 249, 250, 357.2, 362.0, 366.41, V45.85, V53.91, V65.46  **ICD-10:** E8, E9, E10, E11, E13, Z46.81, Z96.41  **ATC:** A10  **Medical procedure code:** 102852, 107015, 107030, 107052, 107074, 109594, 174370, 174381, 174392, 174403, 174414, 174425, 174436, 174440, 174451, 174462, 174473, 174484, 174495, 174506, 174510, 174521, 653671, 653682, 697093, 697104, 754176, 754191, 754250, 754272, 754736, 757352, 757374, 757396, 757411, 757514, 757536, 757551, 770070, 773393, 773496, 784630, 784641, 784652, 784663, 785735, 785750, 785772, 785794, 785816, 785831, 785853, 785875, 785890, 785912, 785934, 785956, 786015, 786030, 786100, 788756, 788771, 788793, 788815, 788830, 788852, 788874, 788896, 788911, 788933, 788955, 789751, 789773, 789795, 789810, 789832, 789854, 789876, 789891, 789913, 789935, 794032, 794113, 794135, 794150, 794194, 794216, 794231, 794253, 794275, 794290, 794312, 794334, 794356, 794371, 794393, 794415, 794430, 794452, 961295, 961306, 961332, 961343 |
| Anemia | | **ICD-9:** 280-285  **ICD-10:** D46.0-D46.4, D50-D53, D56-D64 |
| Dementia  (Alzheimer’s disease, vascular, other) | | **ICD-9:** 046.11, 046.19, 290.0, 290.1, 290.2, 290.3, 290.4, 291.1, 294, 331.0, 331.11, 331.19, 331.82  **ICD-10:** A81.0, F01, F02, F03, F10.27, F10.97, G30, G31.0, G31.83, G31.85  **ATC:** N06D |
| History of falling | | **ICD-9:** E804, E833, E834, E835, E843, E880-E886, E888, E917.5-E917.9, E987, V15.88  **ICD-10:** R29.6, V00.141, V00.811, V00.831, V81.5, V81.6, V82.5, V82.6, V92.0, V93.3, V94.0, V97.0, W00, W01, W03, W05-W15, W16.0-W16.4, W17, W18, W19, Y21.1, Y21.3, Y30, Z91.81 |
| Frailty | | John Hopkins Claims-based Frailty Indicator^16^ (see below) |
| Prior stroke or systemic embolism | | **ICD-9:** 431, 432.9, 433.01, 433.1, 433.21, 433.31, 433.81, 433.91, 434.01, 434.11, 434.91, 436, 438, 444, 557.0, 593.81, V12.54  **ICD-10:** D73.5, I61, I62.9, I63.0, I63.1, I63.2, I63.3, I63.4, I63.5, I63.8, I63.9, I67.89, I69.1, I69.2, I69.3, I69.8, I69.9, I74, K55.01, K55.02, N28.0, Z86.73  **Medical procedure code:** 182136, 182140, 182151, 182162, 182173, 182184, 235130, 235141, 237112, 237123, 477724, 477746, 477761, 477783, 589175, 589186 |
| Prior major or clinically relevant non-major bleeding | | **ICD-9:** 285.1, 287.8, 287.9, 336.1, 360.43, 362.43, 362.81, 363.6, 363.72, 364.41, 372.72, 376.32, 377.42, 379.23, 388.69, 423.0, 430, 431, 432.0, 432.1, 432.9, 455.8, 456.0, 456.20, 459.0, 530.21, 530.7, 530.82, 531.00, 531.20, 531.40, 531.60, 532.00, 532.20, 532.40, 532.60, 533.00, 533.20, 533.40, 533.60, 534.00, 534.20, 534.40, 534.60, 535.01, 535.11, 535.21, 535.31, 535.41, 535.51, 535.61, 535.71, 537.83, 537.84, 562.02, 562.03, 562.12, 562.13, 568.81, 569.3, 569.85, 569.86, 578.0, 578.1, 578.9, 596.7, 599.7, 602.1, 620.7, 621.4, 623.6, 626.2, 626.5, 626.6, 626.7, 626.8, 626.9, 627.0, 627.1, 719.1, 729.92, 784.7, 784.8, 786.30, 786.39, 852.0, 852.2, 852.4, 853.0, 958.2  **ICD-10:** D62, D68.32, D69.8, D69.9, G95.19, H05.23, H11.3, H21.0, H31.3, H31.41, H35.6, H35.73, H43.1, H44.81, H47.02, H92.2x, I23.0, I31.2, I60, I61, I62.0, I62.1, I62.9, I85.01, I85.11, J94.2, J95.01, K22.11, K22.6, K22.8, K25.0, K25.2, K25.4, K25.6, K26.0, K26.2, K26.4, K26.6, K27.0, K27.2, K27.4, K27.6, K28.0, K28.2, K28.4, K28.6, K29.01, K29.21, K29.31, K29.41, K29.51, K29.61, K29.71, K29.81, K29.91, K31.811, K31.82, K50.011, K50.111, K50.811, K50.911, K51.011, K51.211, K51.311, K51.411, K51.511, K51.811, K51.911, K55.21, K57.01, K57.11, K57.13, K57.21, K57.31, K57.33, K57.41, K57.51, K57.53, K57.81, K57.91, K57.93, K62.5, K63.81, K64.9, K66.1, K92.0, K92.1, K92.2, K94.01, K94.11, K94.21, K94.31, M25.0, M79.81, N02, N30.01, N30.11, N30.21, N30.31, N30.41, N30.81, N30.91, N42.1, N83.6, N83.7, N85.7, N89.7, N92.0, N92.1, N92.3, N92.4, N93.0, N93.8, N93.9, N95.0, N99.510, N99.520, N99.530, R04.0, R04.1, R04.2, R04.89, R04.9, R31, R58, S06.340A, S06.341A, S06.342A, S06.343A, S06.344A, S06.345A, S06.346A, S06.347A, S06.348A, S06.349A, S06.350A, S06.351A, S06.352A, S06.353A, S06.354A, S06.355A, S06.356A, S06.357A, S06.358A, S06.359A, S06.360A, S06.361A, S06.362A, S06.363A, S06.364A, S06.365A, S06.366A, S06.367A, S06.368A, S06.369A, S06.4X0A, S06.4X1A, S06.4X2A, S06.4X3A, S06.4X4A, S06.4X5A, S06.4X6A, S06.4X7A, S06.4X8A, S06.4X9A, S06.5X0A, S06.5X1A, S06.5X2A, S06.5X3A, S06.5X4A, S06.5X5A, S06.5X6A, S06.5X7A, S06.5X8A, S06.5X9A, S06.6X0A, S06.6X1A, S06.6X2A, S06.6X3A, S06.6X4A, S06.6X5A, S06.6X6A, S06.6X7A, S06.6X8A, S06.6X9A, T79.2  **Medical procedure code:** 144605, 144620, 144642, 144664, 144686, 227441, 230403, 230425, 230440, 243600, 254940, 255242, 257445, 431620, 431944, 472124, 473686, 473782 |
| **MEDICATION HISTORY (≤6 months before index date)^13^** | | |
| Cardioselective beta-blockers | | **ATC:** C07AB, C07AG, C07BB, C07BG, C07CB, C07CG, C07DB, C07FB, C07FX03, C07FX04, C07FX05, C07FX06, C09BX02, C09BX04, C09BX05, C09DX05 |
| Verapamil, diltiazem | | **ATC:** C08D |
| Digoxin | | **ATC:** C01AA05, C01AA08 |
| Class I AAD | | **ATC:** C01BA, C01BB, C01BC |
| Class III AAD | | **ATC:** C01BD, C07AA07, C07BA07, C07FX02 |
| Acetylsalicylic acid | | **ATC:** B01AC56, B01AC06, C07FX02, C07FX03, C07FX04, C10BX01, C10BX02, C10BX04, C10BX05, C10BX06, C10BX08, C10BX12 |
| P2Y12-inhibitor | | **ATC:** B01AC04, B01AC05, B01AC22 , B01AC24 , B01AC25 |
| Proton pump inhibitor | | **ATC:** A02BC |
| NSAID | | **ATC:** M01AA, M01AB, M01AC, M01AE, M01AG, M01AH, N02AJ08, N02AJ14, N02AJ19, C08CA51 |
| Oral corticosteroid | | **ATC:** H02AB, H02BX01 |
| SSRI/SNRI | | **ATC**: N06AB, N06AX16, N06AX21, N06CA03 |
| **CLINICAL RISK SCORES** | | |
| CHA_2_DS_2_-VASc score^22^ | | - **Congestive heart failure:** 1 point (definition mentioned above: ‘Congestive heart failure’)  - **Hypertension**: 1 point (definition mentioned above: ‘Hypertension’)  - **Age ≥75 years**: 2 point  - **Diabetes mellitus**: 1 point (definition mentioned above: ‘Diabetes mellitus’)  - **Stroke or systemic embolism**: 2 point (definition mentioned above: ‘Thromboembolism (stroke and/or systemic embolism)’)  - **Vascular disease**: 1 point (definition mentioned above: ‘Coronary artery disease’ & ‘Peripheral artery disease’)  - **Age 65-74 years**: 1 point  - **Sex category (female)**: 1 point |
| HAS-BLED score^22^ | | - **Hypertension**: 1 point (definition mentioned above: ‘Hypertension’)  - **Abnormal renal function**: 1 point (definition mentioned above: ‘Chronic kidney disease’)  - **Abnormal liver function**: 1 point (definition mentioned above: ‘Chronic liver disease’)  - **Stroke**: 1 point (definition mentioned above: ‘Stroke’)  - **Bleeding history or predisposition**: 1 point (definition mentioned above: ‘Prior major or clinically relevant non-major bleeding’)  - **Labile** INR: not available  - **Elderly (>65 years or frailty):** 1 point  - **Drugs (antiplatelet, NSAID):** 1 point (definition mentioned above: ‘Antiplatelet’ & ‘NSAID’)  - **Excessive alcohol drinking**: 1 point:   - **ICD-9:** 265.1, 291, 303, 305.0, 357.5, 425.5, 535.3, 571.0-571.3, 980.0, E860.1, V11.3 - **ICD-10:** E51, F10, G31.2, G62.1, G72.1, I42.6, K29.2, K70, K85.2, K86, O35.4, T51.0, T51.9, Z71.4 - **ATC:** N07BB - **Medical procedure code:** 790090 |
| Charlson Comorbidity Index^18,23^ | | - **Myocardial infarction:** 1 point:   - **ICD-9:** 410.00, 410.01, 410.10, 410.11, 410.20, 410.21, 410.30, 410.31, 410.40, 410.41, 410.50, 410.51, 410.60, 410.61, 410.70, 410.71, 410.80, 410.81, 410.90, 410.91 - **ICD-10:** I21, I22   - **Congestive heart failure**: 1 point (definition mentioned above: ‘Congestive heart failure’)  - **Peripheral vascular disease**: 1 point (definition mentioned above: ‘Peripheral artery disease’)  - **Cerebrovascular disease:** 1 point   - **ICD-9:** 362.34, 430, 431, 432, 433, 434, 435, 436, 437, 438 - **ICD-10:** I61, I62, I63, I65, I66, I67, I68, I69, G45, G46, H34.0   - **Dementia**: 1 point (definition mentioned above: ‘Dementia’)  - **Chronic pulmonary disease**: 1 point (definition mentioned above: ‘Chronic lung disease’)  - **Connective tissue disease**: 1 point:   - **ICD-9:** 136.1, 287.0, 446, 447.5, 447.6, 710, 711.2, 725, 728.5, 729.30 - **ICD-10:** D69.0, M30, M31, M32, M33, M34, M35, M36.0, M36.8   - **Peptic ulcer disease**: 1 point:   - **ICD-9:** 041.86, 530.2, 531, 532, 533, 534, V12.71 - **ICD-10:** B96.81, K22.1, K25, K26, K27, K28, Z87.11 - **ATC:** A02BD04, A02BD11 - **Medical procedure code:** 550093, 550104, 552370, 552381   - **Mild liver disease**: 1 point   - **ICD-9:** 070.3, 070.51, 070.52, 070.54, 070.70, 571, 572.8, 573.0, 573.8, 573.9 - **ICD-10:** B17.0, B17.10, B18, B19.10, B19.20, K70.0, K70.1, K70.2, K70.3, K70.9, K71.3, K71.4, K71.5, K71.6, K71.7, K71.8, K71.9, K73, K74, K75.3, K75.4, K75.8, K75.9, K76.0, K76.1, K76.2, K76.89, K76.9, K77 - **ATC:** J05AB04, J05AF05, J05AF07, J05AF08, J05AF10, J05AE11, J05AE12, J05AE14, J05AX15, J05AX65, J05AP - **Medical procedure code:** 556754, 556765   - **Diabetes without chronic complications**: 1 point   - **ICD-9:** 249.4-249.7, 249.9, 250.4-250.7, 250.9, 357.2, 362.0, 364.41 - **ICD-10:** E08.2-E08.5, E08.8, E09.2-E09.5, E09.8, E10.2-E10.5, E10.8, E11.2-E11.5, E11.8, E13.2-E13.5, E13.8 - **Medical procedure code:** 653671, 653682, 697093, 697104, 770070, 773393, 773496   - **Diabetes with chronic complications**: 2 points   - **ICD-9:** 249.0-249.3, 249.8, 250.0-250.3, 250.8 - **ICD-10:** E08.0, E08.1, E08.6, E08.9, E09.0, E09.1, E09.6, E09.9, E10.1, E10.6, E10.9, E11.0, E11.1, E11.6, E11.9, E13.0, E13.1, E13.6, E13.9   - **Hemiplegia or paraplegia**: 2 points:   - **ICD-9:** 334.1, 342, 343.0, 343.1, 343.2, 343.4, 344.0, 344.1, 344.2, 344.9, 438.2 - **ICD-10:** G04.1, G11.4, G80.0, G80.1, G80.2, G81, G82, G83.0, G86.9, I69.05, I69.15, I69.25, I69.35, I69.85, I69.95 - **Medical procedure code:** 643414, 643425   - **Renal disease:** 2 points   - **ICD-9:** 403.01, 403.11, 403.91, 404.02, 404.12, 404.92, 585.5, 585.6, 586, 996.81, V42.0, V45.11, V56 - **ICD-10:** N18.5, N18.6, N19, I12.0, I13.11, T86.1, Z49, Z94.0, Z99.2 - **Medical procedure code:** N81 (group code), 318010, 318021, 318290, 318301   - **Any malignancy, including leukemia and lymphoma:** 2 points:   - **ICD-9:** 140-195, 199.1, 199.2, 200-209, 223, 230-239, 258.0, V58.0, V58.11, V58.12 - **ICD-10:** C00-C76, C80.1, C80.2, C81-C96, D00-D09, D37-D49, E31.2, Z51.0, Z51.11, Z51.12 - **ATC:** L01 - **Medical procedure code:** 154873, 154884, 154895, 154906, 157231, 157242, 201191, 201202, 201213, 201224, 220275, 220286, 220371, 220382, 201213, 201224, 226914, 226925, 226936, 226940, 227216, 227220, 227275, 227286, 227636, 227640, 227651, 227662, 227673, 227684, 227695, 227706, 227710, 227721, 227732, 227743, 227754, 227765, 227776, 227780, 227791, 227802, 227813, 227824, 227835, 227846, 228012, 228023, 228174, 228185, 228233, 228244, 228255, 228266, 228270, 228281, 228292, 228303, 228314, 228325, 228336, 228340, 230473, 230484, 231033, 231044, 241231, 241242, 241415, 241426, 241430, 241441, 241452, 241463, 241555, 241566, 242012, 242023, 242034, 242045, 242292, 242303, 242314, 242325, 242830, 242841, 242852, 242863, 242874, 242885, 242896, 242900, 243051, 243062, 243073, 243084, 243235, 243246, 243736, 243740, 243751, 243762, 243773, 243784, 244016, 244020, 244031, 244042, 244075, 244086, 244790, 244801, 244856, 244860, 244893, 244904, 244915, 244926, 244930, 244941, 244952, 244963, 244974, 244985, 245512, 245523, 245534, 245545, 246050, 246061, 246072, 246083, 247111, 247122, 247133, 247144, 251753, 251764, 251775, 251786, 254892, 254903, 256115, 256126, 256336, 256340, 256572, 256583, 257191, 257202, 258355, 258366, 258370, 258381, 258392, 258403, 258451, 258462, 258554, 258565, 258856, 258860, 258871, 258882, 258893, 258904, 259033, 259044, 259114, 259125, 260190, 260201, 260411, 260422, 260433, 260444, 260551, 260562, 260654, 260665, 260750, 260761, 261111, 261122, 261391, 261402, 261472, 261483, 261671, 261682, 261774, 261785, 261796, 261800, 262334, 262345, 262570, 262581, 277756, 277760, 277771, 277782, 278795, 278806, 278810, 278821, 281831, 281842, 281956, 281960, 282310, 282321, 282671, 282682, 284056, 284060, 288455, 288466, 288470, 288481, 289892, 289903, 291056, 291060, 310494, 310505, 311312, 311323, 312550, 312561, 312572, 312583, 312594, 312605, 312653, 312664, 312970, 312981, 350114, 350125, 350136, 350140, 350276, 350280, 350291, 350302, 350372, 350383, 350674, 350685, 350696, 350700, 431174, 431185, 431336, 431340, 431351, 431362, 432294, 432305, 444113, 444124, 444135, 444146, 444150, 444161, 444172, 444183, 444194, 444205, 444216, 444220, 444231, 444242, 444253, 444264, 444275, 444286, 444290, 444301, 444312, 444323, 444334, 444345, 444474, 444485, 444592, 444603, 473970, 473981, 474795, 474806, 565073, 565084, 565095, 565106, 565110, 565121, 565132, 565143, 565154, 565165, 587834, 587845, 587871, 587882, 587893, 587904, 587915, 587926, 588431, 588442, 588453, 588464, 588475, 588486, 588490, 588501, 588512, 588523, 588534, 588545, 588556, 588560, 588571, 588582, 588593, 588604, 588770, 588781, 588976, 588980, 589691, 589702, 589713, 589724, 589831, 589842, 589875, 589886, 594016, 594020, 594031, 594042, 594053, 594064, 594075, 594086, 594090, 594101, 594112, 594123, 594252, 594263, 594274, 594285, 594296, 594300, 594311, 594322, 594333, 594344, 594355, 594366, 594370, 594381, 594392, 594403, 594414, 594425, 594436, 594440, 594451, 594462, 594495, 594506, 594510, 594521, 594532, 594543, 594554, 594565, 594576, 594580, 594591, 594602, 594613, 594624, 594635, 594646, 594694, 594705, 594716, 594720, 594753, 594764, 594775, 594786, 594790, 594801, 594812, 594823, 594834, 594845, 594856, 594860, 594871, 594882, 594893, 594904, 594915, 594926, 594930, 594941, 598581, 682636, 682640, 682732, 682743, 687934, 687945, 698051, 698062, 698095, 698106, 698390, 698401, 698456, 698460, 698471, 698482, 698493, 698504, 698530, 698541, 745010, 745021, 745032, 745043, 745113, 745124, 745135, 745146, 745150, 745161   - **Moderate or severe liver disease:** 3 points   - **ICD-9:** 070.0, 070.2, 070.4, 070.6, 070.71, 155.0, 155.1, 155.2, 197.7, 456.0, 456.1, 456.2, 567.23, 570, 571.2, 571.5, 571.6, 572.2, 572.3, 572.4, 573.0, 573.5, 789.59, V42.7 - **ICD-10:** B15.0, B16.0, B16.2, B17.11, B19.0, B19.11, B19.21, C22, C78.7, I85, I86.4, K65.2, K70.2, K70.3, K70.4, K71.1, K71.7, K72, K74, K76.1, K76.5, K76.6, K76.7, K76.81, R18.8, Z94.4 - **Medical procedure code:** 318076, 318080, 318334, 318345, 472113, 472124, 589352, 589363   - **Metastatic solid tumor:** 6 points   - **ICD-9:** 196-198, 199.0 - **ICD-10:** C77-C79, C80.0   - **AIDS/HIV:** 6 points   - **ICD-9:** 042, V08 - **ICD-10:** B20, Z21 - **ATC:** J05AE01, J05AE02, J05AE03, J05AE04, J05AE05, J05AE07, J05AE08, J05AE09, J05AE10, J05AF01, J05AF02, J05AF03, J05AF04, J05AF05, J05AF06, J05AF09, J05AF11, J05AF12, J05AF13, J05AG, J05AR, J05AX07, J05AX08, J05AX09, J05AX12   - **Age:**   - <50 years: 0 points - 50-59 years: 1 point - 60-69 years: 2 points - 70-79 years: 3 points - ≥80 years: 4 points   *The following comorbid conditions were mutually exclusive: diabetes with chronic complications and diabetes without chronic complications; mild liver disease and moderate or severe liver disease; and any malignancy and metastatic solid tumor*. |
| John Hopkins Claims-based Frailty Indicator^16^ | | - **Impaired mobility**: beta coefficient 1.24:   - **ICD-9:** 334, 719.7, 781.2, V46.3, V49.84, V57.81 - **ICD-10:** G11, G32.81, M62.3, R26, R29.6, Z74.01, Z74.09, Z99.3 - **Medical procedure code:** N83, 643451, 643462, 653656, 653660, 770394, 770405, 770416, 770420   - **Depression**: beta coefficient 0.54:   - **ICD-9**: 293.83, 296, 300.4, 301.12, 309.0, 309.1, 309.28, 311 - **ICD-10**: F06.31, F06.32, F30, F31, F32, F33, F34.1, F43.21, F43.23 - **ATC:** N06A   - **Congestive heart failure**: beta coefficient 0.50: (definition mentioned above: ‘Congestive heart failure’)  - **Parkinson’s disease**: beta coefficient 0.50: (definition mentioned above: ‘Parkinson’s disease’)  - **White race**: beta coefficient -0.49: not available  - **Arthritis (any type):** beta coefficient 0.43:   - **ICD-9:** 099.3, 696.0, 711.1, 711.3, 713.1, 714.0, 714.1, 714.2, 714.3, 714.4, 714.8, 714.9, 716.5, 716.6, 720.0, 720.2, 720.89, 720.9, V13.4 - **ICD-10:** L40.5, M02.1, M02.3, M05, M06, M07, M08, M13.0, M13.1, M45, M46.1, M46.8, M46.9, Z87.39 - **ATC:** L04AA13, L04AA24, L04AA29, L04AA37 - **Medical procedure code:** 478030, 478041   - **Cognitive impairment**: beta coefficient 0.33:   - **ICD-9:** 331.2, 331.83, 331.89, 331.9, 797 - **ICD-10:** G31.1, G31.84, G31.89, G31.9, R41.81   - **Charlson comorbidity index** **(> 0)**: beta coefficient 0.31  - **Stroke**: beta coefficient 0.28: (definition mentioned above: ‘Stroke’)  - **Paranoia**: beta coefficient 0.24:   - **ICD-9:** 293.81, 293.82, 295, 297, 298 - **ICD-10:** F06.0, F06.2, F20, F22, F23, F24, F28, F29   - **Chronic skin ulcer**: beta coefficient 0.23:   - **ICD-9:** 707 - **ICD-10:** E08.621, E08.622, E09.621, E09.622, E10.621, E10.622, E11.621, E11.622, E13.621, E13.622, L89, L97, L98.4 - **Medical procedure code:** 114074, 114085   - **Pneumonia**: beta coefficient 0.21:   - **ICD-9:** 480, 481, 482, 483, 484, 485, 486, 487.0 - **ICD-10:** A48.1, J11.0, J12, J13, J14, J15, J16, J17, J18   - **Male sex**: beta coefficient -0.19  - **Skin and soft tissue infection**: beta coefficient 0.18:   - **ICD-9:** 680, 681, 682, 683, 684, 685, 686, 695.81 - **ICD-10:** L00, L01, L02, L03, L04, L05, L08   - **Mycoses**: beta coefficient 0.14:   - **ICD-9:** 110, 111, 112, 114, 115, 116, 117, 118 - **ICD-10:** B35, B36, B37, B38, B39, B40, B41, B42, B43, B44, B45, B46, B47, B48, B49   - **Age (in 5 year categories)**: beta coefficient 0.09  - **Admission in past 6 months**: beta coefficient 0.09  - **Gout or other crystal-induced arthropathy**: beta coefficient 0.08:   - **ICD-9:** 274, 712.3, 712.8, 712.90 - **ICD-10:** M10, M11, M1A - **ATC:** M04A   - **Falls**: beta coefficient 0.08: (definition mentioned above: ‘History of falling’)  - **Musculoskeletal problems**: beta coefficient 0.05:   - **ICD-9:** 713, 716.0, 716.2, 716.3, 716.4, 716.5, 716.6, 716.8, 716.9, 718.1, 718.2, 718.5, 718.6, 718.7, 718.8, 718.9, 719-724, 733.0, 733.1, 733.93, 733.94, 733.95, 733.96, 733.97, 733.98, V13.51, V13.52 - **ICD-10:** M07, M12.0, M12.1, M12.2, M12.3, M12.4, M12.8, M12.9, M13, M14, M24.0, M24.3, M24.6, M24.7, M24.8, M24.9, M25, M45, M46.0, M46.1, M46.4, M46.8, M46.9, M47-M51, M53, M54, M80, M81, M84.3, M84.4, M84.5, M84.6, Z87.31   - **Urinary tract infection**: beta coefficient 0.05:   - **ICD-9:** 590.1, 590.8, 590.9, 595.0, 595.4, 595.89, 595.9, 597, 599.0 - **ICD-10:** N30.0, N30.8, N30.9, N10, N12, N13.6, N15.9, N16, N34, N39.0 - **ATC:** J01XE01, J01XX01 |
| **LABORATORY TESTING** | | |
| INR measurement | | **Medical procedure code:** 554573, 554584 |

**eTable 2:** Definition of in- and exclusion criteria, comorbidities, medication history and clinical risk scores based on ICD-coded hospital discharge diagnoses (ICD-9-CM up to 2014 and ICD-10-BE from 2015 onward)^20^, medical procedure codes^21^ and/or ATC-coded prescription claims^13^.

AAD: antiarrhythmic drug; AF: Atrial fibrillation; ATC: Anatomical Therapeutic Chemical Classification; CKD: Chronic kidney disease; ICD-9-CM: International Classification of Diseases (ICD) codes, 9^th^ revision, Clinical Modification; ICD-10-BE: International Classification of Diseases (ICD) codes, 10^th^ Revision, Belgian Modification; INR: International Normalized Ratio; NOAC: non-vitamin K antagonist oral anticoagulant; NSAID: non-steroidal anti-inflammatory drug; OAC: oral anticoagulant; VKA: vitamin K antagonist; y: year.

## eTable 3: Definition of outcomes

| **OUTCOME** | **ICD, ATC AND MEDICAL PROCEDURE CODES** |
| --- | --- |
| Stroke or systemic embolism | **ICD 9:** 431, 432.9, 433.01, 433.1, 433.21, 433.31, 433.81, 433.91, 434.01, 434.11, 434.91, 436, 444, 557.0, 593.81  **ICD 10:** D73.5, I61, I62.9, I63.0, I63.1, I63.2, I63.3, I63.4, I63.5, I63.8, I63.9, I67.89, I74, K55.01, K55.02, N28.0  **Medical procedure code:** 182136, 182140, 182151, 182162, 182173, 182184, 235130, 235141, 237112, 237123, 477724, 477746, 477761, 477783, 589175, 589186 |
| Stroke | **ICD 9:** 431, 432.9, 433.01, 433.1, 433.21, 433.31, 433.81, 433.91, 434.01, 434.11, 434.91, 436  **ICD 10:** I61, I62.9, I63.0, I63.1, I63.2, I63.3, I63.4, I63.5, I63.8, I63.9, I67.89  **Medical procedure code:** 182136, 182140, 182151, 182162, 182173, 182184, 477724, 477746, 477761, 477783 |
| Ischemic stroke | **ICD 9:** 433.01, 433.1, 433.21, 433.31, 433.81, 433.91, 434.01, 434.11, 434.91  **ICD 10:** I63.0, I63.1, I63.2, I63.3, I63.4, I63.5, I63.8, I63.9  **Medical procedure code:** 182136, 182140, 182151, 182162, 182173, 182184 |
| Systemic embolism | **ICD 9:** 444, 557.0, 593.81  **ICD 10:** D73.5, I74, K55.01, K55.02, N28.0  **Medical procedure code:** 235130, 235141, 237112, 237123, 589175, 589186 |
| All-cause mortality | Death of date, based on the year and month of death, not the exact date due to patient privacy |
| Acute myocardial infarction | **ICD 9:** 410.00, 410.01, 410.10, 410.11, 410.20, 410.21, 410.30, 410.31, 410.40, 410.41, 410.50, 410.51, 410.60, 410.61, 410.70, 410.71, 410.80, 410.81, 410.90, 410.91  **ICD 10:** I21, I22 |
| Major or clinically relevant non-major bleeding | **ICD 9:** 336.1, 376.32, 372.72, 364.41, 363.6, 363.72, 362.81, 362.43, 379.23, 360.43, 377.42, 423.0, 430, 431, 432.0, 432.1, 432.9, 455.8, 456.0, 456.20, 530.21, 530.7, 530.82, 531.00, 531.20, 531.40, 531.60, 532.00, 532.20, 532.40, 532.60, 533.00, 533.20, 533.40, 533.60, 534.00, 534.20, 534.40, 534.60, 535.01, 535.11, 535.21, 535.31, 535.41, 535.51, 535.61, 535.71, 537.83, 537.84, 562.02, 562.03, 562.12, 562.13, 568.81, 569.3, 569.85, 569.86, 578.0, 578.1, 578.9, 719.1, 852.0, 852.2, 852.4, 853.0  **ICD 10:** H05.23, H11.3, H21.0, H31.3, H31.41, H35.6, H35.73, H43.1, H44.81, H47.02, I23.0, I31.2, I60, I61, I62.0, I62.1, I62.9, I85.01, I85.11, J94.2, K22.11, K22.6, K22.8, K25.0, K25.2, K25.4, K25.6, K26.0, K26.2, K26.4, K26.6, K27.0, K27.2, K27.4, K27.6, K28.0, K28.2, K28.4, K28.6, K29.01, K29.21, K29.31, K29.41, K29.51, K29.61, K29.71, K29.81, K29.91, K31.811, K31.82, K50.011, K50.111, K50.811, K50.911, K51.011, K51.211, K51.311, K51.411, K51.511, K51.811, K51.911, K55.21, K57.01, K57.11, K57.13, K57.21, K57.31, K57.33, K57.41, K57.51, K57.53, K57.81, K57.91, K57.93, K62.5, K63.81, K64.9, K66.1, K92.0, K92.1, K92.2, K94.01, K94.11, K94.21, K94.31, G95.19, M25.0, S06.340A, S06.341A, S06.342A, S06.343A, S06.344A, S06.345A, S06.346A, S06.347A, S06.348A, S06.349A, S06.350A, S06.351A, S06.352A, S06.353A, S06.354A, S06.355A, S06.356A, S06.357A, S06.358A, S06.359A, S06.360A, S06.361A, S06.362A, S06.363A, S06.364A, S06.365A, S06.366A, S06.367A, S06.368A, S06.369A, S06.4X0A, S06.4X1A, S06.4X2A, S06.4X3A, S06.4X4A, S06.4X5A, S06.4X6A, S06.4X7A, S06.4X8A, S06.4X9A, S06.5X0A, S06.5X1A, S06.5X2A, S06.5X3A, S06.5X4A, S06.5X5A, S06.5X6A, S06.5X7A, S06.5X8A, S06.5X9A, S06.6X0A, S06.6X1A, S06.6X2A, S06.6X3A, S06.6X4A, S06.6X5A, S06.6X6A, S06.6X7A, S06.6X8A, S06.6X9A  **Medical procedure code (hospitalized):** 227441, 230403, 230425, 230440, 243600, 431620, 472124, 473686, 473782 |
| Major bleeding | **ICD 9:** 336.1, 376.32, 372.72, 364.41, 363.6, 363.72, 362.81, 362.43, 379.23, 360.43, 377.42, 423.0, 430, 431, 432.0, 432.1, 432.9, 455.8, 456.0, 456.20, 530.21, 530.7, 530.82, 531.00, 531.20, 531.40, 531.60, 532.00, 532.20, 532.40, 532.60, 533.00, 533.20, 533.40, 533.60, 534.00, 534.20, 534.40, 534.60, 535.01, 535.11, 535.21, 535.31, 535.41, 535.51, 535.61, 535.71, 537.83, 537.84, 562.02, 562.03, 562.12, 562.13, 568.81, 569.3, 569.85, 569.86, 578.0, 578.1, 578.9, 719.1, 852.0, 852.2, 852.4, 853.0  **ICD 10:** H05.23, H11.3, H21.0, H31.3, H31.41, H35.6, H35.73, H43.1, H44.81, H47.02, I23.0, I31.2, I60, I61, I62.0, I62.1, I62.9, I85.01, I85.11, J94.2, K22.11, K22.6, K22.8, K25.0, K25.2, K25.4, K25.6, K26.0, K26.2, K26.4, K26.6, K27.0, K27.2, K27.4, K27.6, K28.0, K28.2, K28.4, K28.6, K29.01, K29.21, K29.31, K29.41, K29.51, K29.61, K29.71, K29.81, K29.91, K31.811, K31.82, K50.011, K50.111, K50.811, K50.911, K51.011, K51.211, K51.311, K51.411, K51.511, K51.811, K51.911, K55.21, K57.01, K57.11, K57.13, K57.21, K57.31, K57.33, K57.41, K57.51, K57.53, K57.81, K57.91, K57.93, K62.5, K63.81, K64.9, K66.1, K92.0, K92.1, K92.2, K94.01, K94.11, K94.21, K94.31, G95.19, M25.0, S06.340A, S06.341A, S06.342A, S06.343A, S06.344A, S06.345A, S06.346A, S06.347A, S06.348A, S06.349A, S06.350A, S06.351A, S06.352A, S06.353A, S06.354A, S06.355A, S06.356A, S06.357A, S06.358A, S06.359A, S06.360A, S06.361A, S06.362A, S06.363A, S06.364A, S06.365A, S06.366A, S06.367A, S06.368A, S06.369A, S06.4X0A, S06.4X1A, S06.4X2A, S06.4X3A, S06.4X4A, S06.4X5A, S06.4X6A, S06.4X7A, S06.4X8A, S06.4X9A, S06.5X0A, S06.5X1A, S06.5X2A, S06.5X3A, S06.5X4A, S06.5X5A, S06.5X6A, S06.5X7A, S06.5X8A, S06.5X9A, S06.6X0A, S06.6X1A, S06.6X2A, S06.6X3A, S06.6X4A, S06.6X5A, S06.6X6A, S06.6X7A, S06.6X8A, S06.6X9A  **Medical procedure code (hospitalized):** 227441, 230403, 230425, 230440, 243600, 431620, 472124, 473686, 473782  ***OR*** Clinically relevant non-major bleeding with blood transfusion or death within 10 days after admission^24,25^: **Medical procedure code (blood transfusion):** 752124, 752463, 752581 |
| Clinically relevant non-major bleeding | **ICD 9:** 285.1, 287.8, 287.9, 388.69, 459.0, 596.7, 599.7, 602.1, 620.7, 621.4, 623.6, 626.2, 626.5, 626.6, 626.7, 626.8, 626.9, 627.0, 627.1, 729.92, 784.7, 784.8, 786.30, 786.39, 958.2  **ICD 10:** D62, D68.32, D69.8, D69.9, H92.2, J95.01, M79.81, N02, N30.01, N30.11, N30.21, N30.31, N30.41, N30.81, N30.91, N42.1, N83.6, N83.7, N85.7, N89.7, N92.0, N92.1, N92.3, N92.4, N93.0, N93.8, N93.9, N95.0, N99.510, N99.520, N99.530, R04.0, R04.1, R04.2, R04.89, R04.9, R31, R58, T79.2  **Medical procedure code (hospitalized):** 144605, 144620, 144642, 144664, 144686, 254940, 255242, 257445, 431944 |
| Intracranial bleeding | **ICD 9:** 430, 431, 432.0, 432.1, 432.9, 852.0, 852.2, 852.4, 853.0  **ICD 10:** I60, I61, I62.0, I62.1, I62.9, S06.340A, S06.341A, S06.342A, S06.343A, S06.344A, S06.345A, S06.346A, S06.347A, S06.348A, S06.349A, S06.350A, S06.351A, S06.352A, S06.353A, S06.354A, S06.355A, S06.356A, S06.357A, S06.358A, S06.359A, S06.360A, S06.361A, S06.362A, S06.363A, S06.364A, S06.365A, S06.366A, S06.367A, S06.368A, S06.369A, S06.4X0A, S06.4X1A, S06.4X2A, S06.4X3A, S06.4X4A, S06.4X5A, S06.4X6A, S06.4X7A, S06.4X8A, S06.4X9A, S06.5X0A, S06.5X1A, S06.5X2A, S06.5X3A, S06.5X4A, S06.5X5A, S06.5X6A, S06.5X7A, S06.5X8A, S06.5X9A, S06.6X0A, S06.6X1A, S06.6X2A, S06.6X3A, S06.6X4A, S06.6X5A, S06.6X6A, S06.6X7A, S06.6X8A, S06.6X9A  **Medical procedure (hospitalized):** 230403, 230425, 230440 |
| Gastrointestinal bleeding | **ICD 9:** 455.8, 456.0, 456.20, 530.21, 530.7, 530.82, 531.00, 531.20, 531.40, 531.60, 532.00, 532.20, 532.40, 532.60, 533.00, 533.20, 533.40, 533.60, 534.00, 534.20, 534.40, 534.60, 535.01, 535.11, 535.21, 535.31, 535.41, 535.51, 535.61, 535.71, 537.83, 537.84, 562.02, 562.03, 562.12, 562.13, 569.3, 569.85, 569.86, 578.0, 578.1, 578.9  **ICD 10:** I85.01, I85.11, K22.11, K22.6, K22.8, K25.0, K25.2, K25.4, K25.6, K26.0, K26.2, K26.4, K26.6, K27.0, K27.2, K27.4, K27.6, K28.0, K28.2, K28.4, K28.6, K29.01, K29.21, K29.31, K29.41, K29.51, K29.61, K29.71, K29.81, K29.91, K31.811, K31.82, K50.011, K50.111, K50.811, K50.911, K51.011, K51.211, K51.311, K51.411, K51.511, K51.811, K51.911, K55.21, K57.01, K57.11, K57.13, K57.21, K57.31, K57.33, K57.41, K57.51, K57.53, K57.81, K57.91, K57.93, K62.5, K63.81, K64.9, K92.0, K92.1, K92.2, K94.01, K94.11, K94.21, K94.31  **Medical procedure (hospitalized):** 472124, 473686, 473782 |
| Upper gastrointestinal bleeding | **ICD 9:** 456.0, 456.20, 530.21, 530.7, 530.82, 531.00, 531.20, 531.40, 531.60, 532.00, 532.20, 532.40, 532.60, 533.00, 533.20, 533.40, 533.60, 534.00, 534.20, 534.40, 534.60, 535.01, 535.11, 535.21, 535.31, 535.41, 535.51, 535.61, 535.71, 537.83, 537.84, 578.0  **ICD 10:** I85.01, I85.11, K22.11, K22.6, K22.8, K25.0, K25.2, K25.4, K25.6, K26.0, K26.2, K26.4, K26.6, K27.0, K27.2, K27.4, K27.6, K28.0, K28.2, K28.4, K28.6, K29.01, K29.21, K29.31, K29.41, K29.51, K29.61, K29.71, K29.81, K29.91, K31.811, K31.82, K92.0, K92.1, K94.21, K94.31  **Medical procedure (hospitalized):** 472124 |
| Lower gastrointestinal bleeding | **ICD 9:** 455.8, 562.02, 562.03, 562.12, 562.13, 569.3, 569.85, 569.86, 578.1, 578.9  **ICD 10:** K50.011, K50.111, K50.811, K50.911, K51.011, K51.211, K51.311, K51.411, K51.511, K51.811, K51.911, K55.21, K57.01, K57.11, K57.13, K57.21, K57.31, K57.33, K57.41, K57.51, K57.53, K57.81, K57.91, K57.93, K62.5, K63.81, K64.9, K92.2, K94.01, K94.11  **Medical procedure (hospitalized):** 473686, 473782 |
| Urogenital bleeding | **ICD 9:** 596.7, 599.7, 602.1, 620.7, 621.4, 623.6, 626.2, 626.5, 626.6, 626.7, 626.8, 626.9, 627.0, 627.1  **ICD 10:** N02, N30.01, N30.11, N30.21, N30.31, N30.41, N30.81, N30.91, N42.1, N83.6, N83.7, N85.7, N89.7, N92.0, N92.1, N92.3, N92.4, N93.0, N93.8, N93.9, N95.0, N99.510, N99.520, N99.530, R31  **Medical procedure (hospitalized):** 431944 |
| Bleeding from other sites | **ICD 9:** 285.1, 287.8, 287.9, 336.1, 376.32, 372.72, 364.41, 363.6, 363.72, 362.81, 362.43, 379.23, 360.43, 377.42, 388.69, 423.0, 459.0, 568.81, 719.1, 729.92, 784.7, 784.8, 786.30, 786.39, 958.2  **ICD 10:** D62, D68.32, D69.8, D69.9, J95.01, H05.23, H11.3, H21.0, H31.3, H31.41, H35.6, H35.73, H43.1, H44.81, H47.02, H92.2, I23.0, I31.2, J94.2, K66.1, G95.19, M25.0, M79.81, R04.0, R04.1, R04.2, R04.89, R04.9, R58, T79.2  **Medical procedure (hospitalized):** 144605, 144620, 144642, 144664, 144686, 227441, 243600, 254940, 255242, 257445, 431620 |

**eTable 3:** Definition of outcomes based on ICD-coded hospital discharge diagnoses (ICD-9-CM up to 2014 and ICD-10-BE from 2015 onward)^20^, medical procedure codes^21^ and/or ATC-coded prescription claims^13^.

ATC: Anatomical Therapeutic Chemical Classification; ICD-9-CM: International Classification of Diseases (ICD) codes, 9^th^ revision, Clinical Modification; ICD-10-BE: International Classification of Diseases (ICD) codes, 10^th^ Revision, Belgian Modification.

## eTable 4: Adjusted HRs of outcomes compared between NOACs and VKAs after IPTW (main analysis)

|  | **NOAC vs VKA** | | **Dabigatran vs VKA** | | **Rivaroxaban vs VKA** | | **Apixaban vs VKA** | | **Edoxaban vs VKA** | |
| --- | --- | --- | --- | --- | --- | --- | --- | --- | --- | --- |
|  | **HR (95%CI)** | **p-value** | **HR (95%CI)** | **p-value** | **HR (95%CI)** | **p-value** | **HR (95%CI)** | **p-value** | **HR (95%CI)** | **p-value** |
| **Effectiveness** |  |  |  |  |  |  |  |  |  |  |
| Stroke or systemic embolism | 0.68 (0.64-0.72) | <0.001 | 0.71 (0.65-0.78) | <0.001 | 0.73 (0.68-0.78) | <0.001 | 0.65 (0.60-0.71) | <0.001 | 0.61 (0.50-0.74) | <0.001 |
| Stroke | 0.73 (0.68-0.78) | <0.001 | 0.77 (0.70-0.86) | <0.001 | 0.79 (0.73-0.85) | <0.001 | 0.74 (0.67-0.81) | <0.001 | 0.69 (0.54-0.87) | 0.002 |
| Ischemic stroke | 0.66 (0.61-0.72) | <0.001 | 0.79 (0.70-0.89) | <0.001 | 0.73 (0.66-0.80) | <0.001 | 0.68 (0.60-0.76) | <0.001 | 0.65 (0.48-0.87) | 0.004 |
| Systemic embolism | 0.53 (0.47-0.60) | <0.001 | 0.56 (0.45-0.69) | <0.001 | 0.55 (0.47-0.63) | <0.001 | 0.42 (0.36-0.50) | <0.001 | 0.48 (0.33-0.68) | <0.001 |
| All-cause mortality | 0.76 (0.74-0.79) | <0.001 | 0.78 (0.74-0.83) | <0.001 | 0.83 (0.79-0.86) | <0.001 | 0.82 (0.78-0.85) | <0.001 | 0.60 (0.54-0.68) | <0.001 |
| Myocardial infarction | 0.94 (0.84-1.06) | 0.311 | 1.06 (0.91-1.25) | 0.454 | 0.94 (0.83-1.06) | 0.319 | 1.01 (0.88-1.16) | 0.872 | 0.86 (0.64-1.17) | 0.348 |
| **Safety** |  |  |  |  |  |  |  |  |  |  |
| MB/CRNMB | 0.94 (0.91-0.98) | <0.001 | 0.88 (0.84-0.93) | <0.001 | 1.00 (0.96-1.04) | 0.923 | 0.87 (0.83-0.91) | <0.001 | 0.86 (0.78-0.95) | 0.004 |
| Major bleeding | 0.88 (0.84-0.92) | <0.001 | 0.91 (0.85-0.97) | 0.007 | 0.99 (0.94-1.04) | 0.719 | 0.78 (0.73-0.83) | <0.001 | 0.93 (0.80-1.07) | 0.304 |
| CRNMB | 0.99 (0.94-1.04) | 0.754 | 0.85 (0.79-0.92) | <0.001 | 1.00 (0.94-1.05) | 0.872 | 0.92 (0.87-0.98) | 0.012 | 0.82 (0.72-0.93) | 0.002 |
| Intracranial hemorrhage | 0.73 (0.66-0.79) | <0.001 | 0.81 (0.71-0.92) | <0.001 | 0.86 (0.78-0.95) | 0.002 | 0.72 (0.64-0.82) | <0.001 | 0.93 (0.65-1.33) | 0.699 |
| Gastrointestinal bleeding | 1.06 (0.98-1.14) | 0.122 | 1.12 (1.01-1.23) | 0.027 | 1.21 (1.12-1.30) | <0.001 | 0.86 (0.78-0.94) | <0.001 | 1.15 (0.93-1.42) | 0.212 |
| Upper gastrointestinal bleeding | 1.05 (0.95-1.16) | 0.344 | 1.00 (0.87-1.15) | 0.995 | 1.13 (1.01-1.25) | 0.028 | 0.91 (0.80-1.03) | 0.121 | 1.15 (0.86-1.54) | 0.350 |
| Lower gastrointestinal bleeding | 1.02 (0.93-1.12) | 0.632 | 1.11 (0.98-1.25) | 0.103 | 1.25 (1.14-1.37) | <0.001 | 0.79 (0.70-0.88) | <0.001 | 1.13 (0.86-1.50) | 0.377 |
| Urogenital bleeding | 1.07 (0.98-1.18) | 0.131 | 0.97 (0.86-1.11) | 0.700 | 1.18 (1.07-1.30) | <0.001 | 0.93 (0.83-1.05) | 0.233 | 0.84 (0.65-1.09) | 0.183 |
| Bleeding from other sites | 0.89 (0.85-0.94) | <0.001 | 0.76 (0.71-0.82) | <0.001 | 0.89 (0.85-0.94) | <0.001 | 0.84 (0.79-0.89) | <0.001 | 0.77 (0.68-0.86) | <0.001 |

**eTable 4:** Adjusted hazard ratios of outcomes compared between NOACs and VKAs after IPTW.

CI: confidence interval; CRNMB: clinically relevant non-major bleeding; HR: hazard ratio; IPTW: inverse probability of treatment weighting; MB: major bleeding; NOAC: non-vitamin K antagonist oral anticoagulant; VKA: vitamin K antagonist; vs: versus.

## eTable 5: Adjusted HRs of outcomes compared between individual NOACs after IPTW (main analysis)

|  | **Dabigatran vs Rivaroxaban** | | **Apixaban vs Rivaroxaban** | | **Edoxaban vs Rivaroxaban** | | **Apixaban vs Dabigatran** | | **Dabigatran vs Edoxaban** | | **Apixaban vs Edoxaban** | |
| --- | --- | --- | --- | --- | --- | --- | --- | --- | --- | --- | --- | --- |
|  | **HR (95%CI)** | **p-value** | **HR (95%CI)** | **p-value** | **HR (95%CI)** | **p-value** | **HR (95%CI)** | **p-value** | **HR (95%CI)** | **p-value** | **HR (95%CI)** | **p-value** |
| **Effectiveness** |  |  |  |  |  |  |  |  |  |  |  |  |
| Stroke or systemic embolism | 0.92 (0.85-0.99) | 0.043 | 0.93 (0.87-0.99) | 0.027 | 0.94 (0.81-1.09) | 0.402 | 1.03 (0.93-1.13) | 0.586 | 1.02 (0.84-1.24) | 0.828 | 0.97 (0.85-1.10) | 0.649 |
| Stroke | 0.91 (0.83-0.99) | 0.025 | 0.95 (0.88-1.02) | 0.131 | 0.95 (0.80-1.12) | 0.509 | 1.05 (0.95-1.16) | 0.337 | 1.06 (0.86-1.32) | 0.565 | 1.00 (0.87-1.16) | 0.952 |
| Ischemic stroke | 1.02 (0.92-1.13) | 0.761 | 0.95 (0.87-1.04) | 0.282 | 0.97 (0.78-1.20) | 0.786 | 0.94 (0.83-1.07) | 0.374 | 1.24 (0.96-1.61) | 0.099 | 1.05 (0.88-1.27) | 0.577 |
| Systemic embolism | 1.06 (0.88-1.28) | 0.548 | 0.84 (0.72-0.99) | 0.044 | 0.93 (0.69-1.26) | 0.650 | 0.84 (0.67-1.05) | 0.123 | 0.98 (0.65-1.48) | 0.919 | 0.80 (0.61-1.06) | 0.128 |
| All-cause mortality | 0.88 (0.84-0.92) | <0.001 | 1.00 (0.96-1.03) | 0.818 | 0.89 (0.82-0.96) | 0.003 | 1.17 (1.12-1.23) | <0.001 | 0.93 (0.83-1.04) | 0.206 | 1.17 (1.09-1.25) | <0.001 |
| Myocardial infarction | 1.04 (0.91-1.18) | 0.566 | 1.02 (0.93-1.13) | 0.635 | 0.89 (0.73-1.09) | 0.255 | 1.02 (0.89-1.18) | 0.763 | 1.15 (0.88-1.50) | 0.293 | 0.99 (0.83-1.18) | 0.895 |
| **Safety** |  |  |  |  |  |  |  |  |  |  |  |  |
| MB/CRNMB | 0.86 (0.83-0.90) | <0.001 | 0.86 (0.83-0.89) | <0.001 | 0.95 (0.89-1.01) | 0.123 | 0.98 (0.93-1.03) | 0.345 | 0.91 (0.83-0.99) | 0.046 | 0.86 (0.81-0.91) | <0.001 |
| Major bleeding | 0.89 (0.85-0.94) | <0.001 | 0.77 (0.74-0.81) | <0.001 | 0.95 (0.87-1.04) | 0.295 | 0.86 (0.80-0.92) | <0.001 | 0.97 (0.85-1.11) | 0.678 | 0.79 (0.72-0.86) | <0.001 |
| CRNMB | 0.83 (0.79-0.89) | <0.001 | 0.94 (0.90-0.98) | 0.004 | 0.93 (0.86-1.02) | 0.113 | 1.09 (1.02-1.17) | 0.008 | 0.85 (0.75-0.96) | 0.007 | 0.91 (0.85-0.99) | 0.021 |
| Intracranial hemorrhage | 0.91 (0.82-1.01) | 0.077 | 0.88 (0.80-0.97) | 0.007 | 1.10 (0.88-1.38) | 0.389 | 0.98 (0.86-1.12) | 0.801 | 0.91 (0.67-1.24) | 0.546 | 1.06 (0.88-1.28) | 0.540 |
| Gastrointestinal bleeding | 0.90 (0.84-0.97) | 0.005 | 0.68 (0.63-0.72) | <0.001 | 0.88 (0.78-0.99) | 0.037 | 0.74 (0.68-0.81) | <0.001 | 0.99 (0.83-1.17) | 0.907 | 0.68 (0.61-0.77) | <0.001 |
| Upper gastrointestinal bleeding | 0.89 (0.80-0.99) | 0.030 | 0.76 (0.69-0.82) | <0.001 | 0.81 (0.69-0.96) | 0.014 | 0.79 (0.70-0.89) | <0.001 | 1.08 (0.85-1.37) | 0.527 | 0.78 (0.67-0.91) | 0.002 |
| Lower gastrointestinal bleeding | 0.86 (0.78-0.94) | <0.001 | 0.60 (0.55-0.65) | <0.001 | 0.86 (0.74-1.00) | 0.051 | 0.74 (0.66-0.82) | <0.001 | 0.96 (0.77-1.20) | 0.709 | 0.61 (0.52-0.71) | <0.001 |
| Urogenital bleeding | 0.82 (0.75-0.91) | <0.001 | 0.83 (0.77-0.89) | <0.001 | 0.82 (0.71-0.95) | 0.007 | 0.95 (0.85-1.06) | 0.367 | 0.83 (0.66-1.04) | 0.107 | 0.81 (0.71-0.93) | 0.003 |
| Bleeding from other sites | 0.83 (0.79-0.89) | <0.001 | 0.93 (0.89-0.97) | <0.001 | 0.95 (0.87-1.04) | 0.252 | 1.07 (1.01-1.15) | 0.046 | 0.88 (0.78-0.99) | 0.045 | 0.90 (0.83-0.97) | 0.006 |

**eTable 5:** Adjusted hazard ratios of outcomes compared between individual NOACs after IPTW.

CI: confidence interval; CRNMB: clinically relevant non-major bleeding; HR: hazard ratio; IPTW: inverse probability of treatment weighting; MB: major bleeding; NOAC: non-vitamin K antagonist oral anticoagulant; vs: versus.

## eTable 6: Baseline characteristics of standard and reduced dose NOACs versus VKAs

**A)**

| **Patient characteristics** | | **VKA**  **(n = 61406)** | **Standard dose NOAC** | | | | | **SMD^*^** | |
| --- | --- | --- | --- | --- | --- | --- | --- | --- | --- |
|  |  |  | **Overall**  **(n = 121230)** | **Dabigatran**  **(n = 12662)** | **Rivaroxaban**  **(n = 44703)** | **Apixaban**  **(n = 47261)** | **Edoxaban**  **(n = 16604)** | **Before IPTW** | **After IPTW** |
|  | Age (years) | 70.9 +/- 12.1 | 73.0 +/- 9.3 | 69.2 +/- 7.8 | 72.6 +/- 9.5 | 74.5 +/- 9.1 | 73.0 +/- 9.6 | 0.197 | 0.005 |
|  | <65 years | 21158 (34.5%) | 20590 (17.0%) | 3075 (24.3%) | 8213 (18.4%) | 6270 (13.3%) | 3032 (18.3%) | NA | NA |
|  | 65-74 years | 15128 (24.6%) | 47319 (39.0%) | 6538 (51.6%) | 17516 (39.2%) | 16957 (35.9%) | 6308 (38.0%) | NA | NA |
|  | 75-84 years | 16798 (27.4%) | 42183 (34.8%) | 2927 (23.1%) | 15280 (34.2%) | 18390 (38.9%) | 5586 (33.6%) | NA | NA |
|  | ≥85 years | 8322 (13.6%) | 11138 ( 9.2%) | 122 ( 1.0%) | 3694 ( 8.3%) | 5644 (11.9%) | 1678 (10.1%) | NA | NA |
|  | Female | 28766 (46.8%) | 50745 (41.9%) | 4829 (38.1%) | 18514 (41.4%) | 20761 (43.9%) | 6641 (40.0%) | 0.101 | 0.01 |
|  | Follow-up (years) | 0.8 +/- 1.3 | 1.5 +/- 1.5 | 1.7 +/- 1.7 | 1.8 +/- 1.7 | 1.5 +/- 1.4 | 0.7 +/- 0.6 | NA | NA |
| **Comorbidities** | | | | | | | | | |
|  | Hypertension | 36945 (60.2%) | 77695 (64.1%) | 7905 (62.4%) | 27805 (62.2%) | 31764 (67.2%) | 10221 (61.6%) | 0.082 | 0.008 |
|  | Coronary artery disease | 14142 (23.0%) | 19350 (16.0%) | 1646 (13.0%) | 6865 (15.4%) | 8261 (17.5%) | 2578 (15.5%) | 0.176 | 0.025 |
|  | Congestive heart failure | 9779 (15.9%) | 15453 (12.7%) | 1184 (9.4%) | 5487 (12.3%) | 6866 (14.5%) | 1915 (11.5%) | 0.087 | 0.023 |
|  | Valvular heart disease | 11889 (19.4%) | 12772 (10.5%) | 1036 (8.2%) | 4197 (9.4%) | 5641 (11.9%) | 1898 (11.4%) | 0.251 | 0.014 |
|  | Peripheral artery disease | 6866 (11.2%) | 7881 (6.5%) | 767 (6.1%) | 2571 (5.8%) | 3639 (7.7%) | 904 (5.4%) | 0.155 | 0.009 |
|  | Dyslipidemia | 34288 (55.8%) | 69955 (57.7%) | 7542 (59.6%) | 24699 (55.3%) | 28310 (59.9%) | 9404 (56.6%) | 0.038 | 0.006 |
|  | Chronic kidney disease | 8607 (14.0%) | 7298 (6.0%) | 406 (3.2%) | 2081 (4.7%) | 3973 (8.4%) | 838 (5.0%) | 0.256 | 0.009 |
|  | Chronic liver disease | 2563 (4.2%) | 3538 (2.9%) | 323 (2.5%) | 1292 (2.9%) | 1459 (3.1%) | 465 (2.8%) | 0.058 | 0.002 |
|  | Chronic lung disease | 8428 (13.7%) | 13678 (11.3%) | 1253 (9.9%) | 5012 (11.2%) | 5714 (12.1%) | 1699 (10.2%) | 0.065 | 0.002 |
|  | Obstructive sleep apnea | 2280 (3.7%) | 5037 (4.2%) | 643 (5.1%) | 1792 (4.0%) | 1890 (4.0%) | 712 (4.3%) | 0.028 | 0.018 |
|  | Cancer | 5939 (9.7%) | 11184 (9.2%) | 1013 (8.0%) | 4031 (9.0%) | 4614 (9.8%) | 1526 (9.2%) | 0.008 | 0.012 |
|  | Upper GI tract disorder^**^ | 5163 (8.4%) | 7746 (6.4%) | 686 (5.4%) | 2883 (6.4%) | 3279 (6.9%) | 899 (5.4%) | 0.068 | 0.011 |
|  | Lower GI tract disorder^**^ | 4175 (6.8%) | 8405 (6.9%) | 794 (6.3%) | 3039 (6.8%) | 3422 (7.2%) | 1149 (6.9%) | 0.016 | 0.005 |
|  | Diabetes mellitus | 22453 (36.6%) | 35631 (29.4%) | 3398 (26.8%) | 12576 (28.1%) | 15054 (31.9%) | 4603 (27.7%) | 0.149 | 0.018 |
|  | Anemia | 6485 (10.6%) | 6745 (5.6%) | 488 (3.9%) | 2373 (5.3%) | 3070 (6.5%) | 815 (4.9%) | 0.170 | 0.005 |
|  | Dementia | 2809 (4.6%) | 4092 (3.4%) | 201 (1.6%) | 1494 (3.3%) | 1947 (4.1%) | 449 (2.7%) | 0.054 | 0.015 |
|  | History of falling | 4003 (6.5%) | 7582 (6.3%) | 542 (4.3%) | 2429 (5.4%) | 3575 (7.6%) | 1036 (6.2%) | 0.002 | 0.04 |
|  | Frailty | 13746 (22.4%) | 20811 (17.2%) | 713 (5.6%) | 7000 (15.7%) | 10464 (22.1%) | 2634 (15.9%) | 0.128 | 0.018 |
|  | Prior stroke/SE | 8759 (14.3%) | 15517 (12.8%) | 2218 (17.5%) | 4160 (9.3%) | 7763 (16.4%) | 1375 (8.3%) | 0.031 | 0.012 |
|  | Prior MB/CRNMB | 4008 (6.5%) | 5388 (4.4%) | 501 (4.0%) | 1802 (4.0%) | 2444 (5.2%) | 641 (3.9%) | 0.077 | 0.004 |
| **Medication history** | | | | | | | | | |
|  | Number of concomitant drugs | 6.8 +/- 4.5 | 6.2 +/- 4.0 | 5.8 +/- 3.7 | 6.2 +/- 4.0 | 6.6 +/- 4.2 | 5.9 +/- 3.8 | 0.138 | 0.007 |
|  | Beta blockers | 31820 (51.8%) | 75188 (62.0%) | 7715 (60.9%) | 27141 (60.7%) | 30040 (63.6%) | 10292 (62.0%) | 0.207 | 0.012 |
|  | Verapamil, diltiazem | 2162 (3.5%) | 4970 (4.1%) | 510 (4.0%) | 1974 (4.4%) | 1952 (4.1%) | 534 (3.2%) | 0.030 | 0.021 |
|  | Digoxin | 3694 (6.0%) | 10684 (8.8%) | 976 (7.7%) | 3810 (8.5%) | 4547 (9.6%) | 1351 (8.1%) | 0.107 | 0.012 |
|  | Class I AAD | 3490 (5.7%) | 14704 (12.1%) | 1798 (14.2%) | 6031 (13.5%) | 4855 (10.3%) | 2020 (12.2%) | 0.228 | 0.005 |
|  | Class III AAD | 11578 (18.9%) | 31359 (25.9%) | 3231 (25.5%) | 12454 (27.9%) | 12121 (25.6%) | 3553 (21.4%) | 0.169 | 0.029 |
|  | Acetylsalicylic acid | 21648 (35.3%) | 47570 (39.2%) | 4802 (37.9%) | 17268 (38.6%) | 19240 (40.7%) | 6260 (37.7%) | 0.083 | 0.007 |
|  | P2Y12 inhibitor | 3391 (5.5%) | 6214 (5.1%) | 538 (4.2%) | 2003 (4.5%) | 2691 (5.7%) | 982 (5.9%) | 0.018 | 0.01 |
|  | Proton pump inhibitor | 25707 (41.9%) | 45303 (37.4%) | 4365 (34.5%) | 16406 (36.7%) | 18526 (39.2%) | 6006 (36.2%) | 0.092 | 0.013 |
|  | NSAID | 16501 (26.9%) | 30920 (25.5%) | 3384 (26.7%) | 11487 (25.7%) | 11790 (24.9%) | 4259 (25.7%) | 0.031 | 0.004 |
|  | Oral corticosteroids | 13892 (22.6%) | 22787 (18.8%) | 2164 (17.1%) | 8448 (18.9%) | 9121 (19.3%) | 3054 (18.4%) | 0.095 | 0.008 |
|  | SSRI/SNRI | 8233 (13.4%) | 13089 (10.8%) | 1253 (9.9%) | 4917 (11.0%) | 5427 (11.5%) | 1492 (9.0%) | 0.080 | 0.013 |
| **Clinical risk score** | | | | | | | | | |
|  | CHA_2_DS_2_-VASc score | 3.2 +/- 2.0 | 3.2 +/- 1.7 | 2.9 +/- 1.6 | 3.0 +/- 1.7 | 3.5 +/- 1.7 | 3.0 +/- 1.6 | 0.004 | 0.014 |
|  | HAS-BLED score | 2.3 +/- 1.4 | 2.4 +/- 1.2 | 2.3 +/- 1.2 | 2.3 +/- 1.1 | 2.5 +/- 1.2 | 2.3 +/- 1.1 | 0.075 | 0.007 |
|  | Charlson Comorbidity Index | 4.0 +/- 2.5 | 4.0 +/- 2.1 | 3.5 +/- 1.9 | 3.9 +/- 2.1 | 4.3 +/- 2.1 | 3.9 +/- 2.0 | 0.005 | 0.003 |

**B)**

| **Patient characteristics** | | **VKA**  **(n = 61406)** | **Reduced dose NOAC** | | | | | **SMD^*^** | |
| --- | --- | --- | --- | --- | --- | --- | --- | --- | --- |
|  |  |  | **Overall**  **(n = 71842)** | **Dabigatran**  **(n = 15482)** | **Rivaroxaban**  **(n = 29718)** | **Apixaban**  **(n = 19664)** | **Edoxaban**  **(n = 6978)** | **Before IPTW** | **After IPTW** |
|  | Age (years) | 70.9 +/- 12.1 | 81.7 +/- 9.0 | 81.5 +/- 7.5 | 80.1 +/- 10.1 | 84.1 +/- 8.0 | 82.5 +/- 8.6 | 1.013 | 0.009 |
|  | <65 years | 21158 (34.5%) | 4151 ( 5.8%) | 557 ( 3.6%) | 2628 ( 8.8%) | 670 ( 3.4%) | 296 ( 4.2%) | NA | NA |
|  | 65-74 years | 15128 (24.6%) | 8387 (11.7%) | 1799 (11.6%) | 4179 (14.1%) | 1505 ( 7.7%) | 904 (13.0%) | NA | NA |
|  | 75-84 years | 16798 (27.4%) | 30334 (42.2%) | 7964 (51.4%) | 12474 (42.0%) | 7224 (36.7%) | 2672 (38.3%) | NA | NA |
|  | ≥85 years | 8322 (13.6%) | 28970 (40.3%) | 5162 (33.3%) | 10437 (35.1%) | 10265 (52.2%) | 3106 (44.5%) | NA | NA |
|  | Female | 28766 (46.8%) | 41263 (57.4%) | 8301 (53.6%) | 16674 (56.1%) | 12131 (61.7%) | 4157 (59.6%) | 0.213 | 0.019 |
|  | Follow-up (years) | 0.8 +/- 1.3 | 1.3 +/- 1.4 | 1.5 +/- 1.6 | 1.5 +/- 1.5 | 1.1 +/- 1.2 | 0.7 +/- 0.6 | NA | NA |
| **Comorbidities** | | | | | | | | | |
|  | Hypertension | 36945 (60.2%) | 50236 (69.9%) | 10477 (67.7%) | 19980 (67.2%) | 14713 (74.8%) | 5066 (72.6%) | 0.206 | 0.018 |
|  | Coronary artery disease | 14142 (23.0%) | 14351 (20.0%) | 2668 (17.2%) | 5398 (18.2%) | 4667 (23.7%) | 1618 (23.2%) | 0.074 | 0.007 |
|  | Congestive heart failure | 9779 (15.9%) | 14661 (20.4%) | 2397 (15.5%) | 5354 (18.0%) | 5231 (26.6%) | 1680 (24.1%) | 0.115 | 0.001 |
|  | Valvular heart disease | 11889 (19.4%) | 11501 (16.0%) | 2073 (13.4%) | 3948 (13.3%) | 4032 (20.5%) | 1448 (20.8%) | 0.097 | 0.01 |
|  | Peripheral artery disease | 6866 (11.2%) | 6189 (8.6%) | 1099 (7.1%) | 2294 (7.7%) | 2119 (10.8%) | 678 (9.7%) | 0.081 | 0.003 |
|  | Dyslipidemia | 34288 (55.8%) | 39672 (55.2%) | 8791 (56.8%) | 15811 (53.2%) | 11063 (56.3%) | 4007 (57.4%) | 0.012 | 0.01 |
|  | Chronic kidney disease | 8607 (14.0%) | 13590 (18.9%) | 1455 (9.4%) | 4788 (16.1%) | 5311 (27.0%) | 2035 (29.2%) | 0.135 | 0.031 |
|  | Chronic liver disease | 2563 (4.2%) | 2355 (3.3%) | 415 (2.7%) | 943 (3.2%) | 717 (3.6%) | 280 (4.0%) | 0.044 | 0.005 |
|  | Chronic lung disease | 8428 (13.7%) | 9940 (13.8%) | 1786 (11.5%) | 3916 (13.2%) | 3140 (16.0%) | 1097 (15.7%) | 0.009 | 0.008 |
|  | Obstructive sleep apnea | 2280 (3.7%) | 1456 (2.0%) | 236 (1.5%) | 689 (2.3%) | 389 (2.0%) | 142 (2.0%) | 0.097 | 0.009 |
|  | Cancer | 5939 (9.7%) | 8064 (11.2%) | 1467 (9.5%) | 3338 (11.2%) | 2315 (11.8%) | 944 (13.5%) | 0.054 | 0.018 |
|  | Upper GI tract disorder^**^ | 5163 (8.4%) | 6269 (8.7%) | 1063 (6.9%) | 2515 (8.5%) | 2127 (10.8%) | 565 (8.1%) | 0.015 | 0.004 |
|  | Lower GI tract disorder^**^ | 4175 (6.8%) | 5077 (7.1%) | 996 (6.4%) | 2067 (7.0%) | 1520 (7.7%) | 494 (7.1%) | 0.015 | 0.002 |
|  | Diabetes mellitus | 22453 (36.6%) | 24619 (34.3%) | 4492 (29.0%) | 9346 (31.4%) | 7997 (40.7%) | 2784 (39.9%) | 0.05 | 0.023 |
|  | Anemia | 6485 (10.6%) | 7882 (11.0%) | 1138 (7.4%) | 2909 (9.8%) | 2905 (14.8%) | 930 (13.3%) | 0.018 | 0.019 |
|  | Dementia | 2809 (4.6%) | 6661 (9.3%) | 1096 (7.1%) | 2552 (8.6%) | 2365 (12.0%) | 649 (9.3%) | 0.188 | 0.002 |
|  | History of falling | 4003 (6.5%) | 8588 (12.0%) | 1275 (8.2%) | 2861 (9.6%) | 3354 (17.1%) | 1098 (15.7%) | 0.191 | 0.041 |
|  | Frailty | 13746 (22.4%) | 37927 (52.8%) | 7022 (45.4%) | 13841 (46.6%) | 13159 (66.9%) | 3906 (56.0%) | 0.664 | 0.029 |
|  | Prior stroke/SE | 8759 (14.3%) | 11119 (15.5%) | 2657 (17.2%) | 3654 (12.3%) | 3934 (20.0%) | 874 (12.5%) | 0.039 | 0.014 |
|  | Prior MB/CRNMB | 4008 (6.5%) | 4883 (6.8%) | 878 (5.7%) | 1767 (5.9%) | 1709 (8.7%) | 529 (7.6%) | 0.016 | 0.011 |
| **Comedication** | | | | | | | | | |
|  | Number of concomitant drugs | 6.8 +/- 4.5 | 7.3 +/- 4.2 | 6.6 +/- 3.8 | 7.1 +/- 4.2 | 7.9 +/- 4.5 | 7.6 +/- 4.3 | 0.098 | 0.021 |
|  | Beta blockers | 31820 (51.8%) | 44809 (62.4%) | 9486 (61.3%) | 17537 (59.0%) | 13093 (66.6%) | 4693 (67.3%) | 0.214 | 0.009 |
|  | Verapamil, diltiazem | 2162 (3.5%) | 2771 (3.9%) | 637 (4.1%) | 1218 (4.1%) | 675 (3.4%) | 241 (3.5%) | 0.018 | 0.013 |
|  | Digoxin | 3694 (6.0%) | 8153 (11.3%) | 1588 (10.3%) | 3124 (10.5%) | 2564 (13.0%) | 877 (12.6%) | 0.19 | 0.012 |
|  | Class I AAD | 3490 (5.7%) | 5107 (7.1%) | 1270 (8.2%) | 2165 (7.3%) | 1169 (5.9%) | 503 (7.2%) | 0.058 | 0.016 |
|  | Class III AAD | 11578 (18.9%) | 18514 (25.8%) | 4011 (25.9%) | 7694 (25.9%) | 5096 (25.9%) | 1713 (24.5%) | 0.167 | 0.027 |
|  | Acetylsalicylic acid | 21648 (35.3%) | 30763 (42.8%) | 6628 (42.8%) | 12296 (41.4%) | 8820 (44.9%) | 3019 (43.3%) | 0.156 | 0.004 |
|  | P2Y12 inhibitor | 3391 (5.5%) | 5076 (7.1%) | 957 (6.2%) | 1866 (6.3%) | 1542 (7.8%) | 711 (10.2%) | 0.064 | 0.022 |
|  | Proton pump inhibitor | 25707 (41.9%) | 31238 (43.5%) | 6083 (39.3%) | 12513 (42.1%) | 9339 (47.5%) | 3303 (47.3%) | 0.033 | 0.027 |
|  | NSAID | 16501 (26.9%) | 15561 (21.7%) | 3472 (22.4%) | 7078 (23.8%) | 3723 (18.9%) | 1288 (18.5%) | 0.122 | 0.004 |
|  | Oral corticosteroids | 13892 (22.6%) | 15460 (21.5%) | 2945 (19.0%) | 6554 (22.1%) | 4386 (22.3%) | 1575 (22.6%) | 0.027 | 0.007 |
|  | SSRI/SNRI | 8233 (13.4%) | 10005 (13.9%) | 2030 (13.1%) | 4231 (14.2%) | 2909 (14.8%) | 835 (12.0%) | 0.015 | 0.004 |
| **Clinical risk score** | | | | | | | | | |
|  | CHA_2_DS_2_-VASc score | 3.2 +/- 2.0 | 4.1 +/- 1.7 | 4.0 +/- 1.6 | 3.9 +/- 1.7 | 4.6 +/- 1.7 | 4.3 +/- 1.6 | 0.507 | 0.004 |
|  | HAS-BLED score | 2.3 +/- 1.4 | 2.8 +/- 1.2 | 2.7 +/- 1.1 | 2.6 +/- 1.3 | 3.0 +/- 1.3 | 2.9 +/- 1.3 | 0.365 | 0.004 |
|  | Charlson Comorbidity Index | 4.0 +/- 2.5 | 5.1 +/- 2.2 | 5.0 +/- 2.0 | 4.9 +/- 2.3 | 5.6 +/- 2.2 | 5.3 +/- 2.1 | 0.505 | 0.015 |

**eTable 6:** Baseline characteristics of OAC-naïve AF patients treated with **A)** standard dose NOACs or VKAs, and **B)** reduced dose NOACs or VKAs.

Data shown as mean ± standard deviation, or counts and percentages. VKA users included 29 650 acenocoumarol, 16 859 warfarin and 14 897 phenprocoumon users. *Absolute SMDs illustrated for comparison of NOACs versus VKAs before and after inverse probability of treatment weighting. **Upper and lower gastrointestinal tract disorders were defined as gastroesophageal reflux disease or peptic ulcer disease; and diverticulosis, angiodysplasia, colorectal polyposis or hemorrhoids, respectively. AAD: antiarrhythmic drug; AF: atrial fibrillation; CRNMB: clinically relevant non-major bleeding; GI: gastrointestinal; MB: major bleeding; NA: not applicable; NOAC: non-vitamin K antagonist oral anticoagulant; NSAID: non-steroidal anti-inflammatory drug; OAC: oral anticoagulant; SE: systemic embolism; SMD: standardized mean difference; SNRI: serotonin and norepinephrine reuptake inhibitor; SSRI: selective serotonin reuptake inhibitor; VKA: vitamin K antagonist.

eTable 7: Number of events and crude event rates of outcomes with standard and reduced dose NOACs

**A) Standard dose NOACs**

| **Outcome** | **NOAC**  **(n = 121230)** | **Dabigatran**  **(n = 12662)** | **Rivaroxaban**  **(n = 44703)** | **Apixaban**  **(n = 47261)** | **Edoxaban**  **(n = 16604)** |
| --- | --- | --- | --- | --- | --- |
|  | **Events (per 100 PY)** | **Events (per 100 PY)** | **Events (per 100 PY)** | **Events (per 100 PY)** | **Events (per 100 PY)** |
| **Effectiveness** |  |  |  |  |  |
| Stroke or systemic embolism | 3206 (1.79) | 330 (1.55) | 1325 (1.71) | 1303 (1.90) | 248 (2.05) |
| Stroke | 2733 (1.52) | 273 (1.28) | 1128 (1.45) | 1133 (1.65) | 199 (1.64) |
| Ischemic stroke | 1608 (0.89) | 181 (0.84) | 672 (0.86) | 642 (0.93) | 113 (0.93) |
| Systemic embolism | 554 (0.30) | 72 (0.33) | 225 (0.29) | 202 (0.29) | 55 (0.45) |
| Mortality | 8358 (4.59) | 561 (2.59) | 3507 (4.46) | 3731 (5.35) | 559 (4.57) |
| Myocardial infarction | 1284 (0.71) | 119 (0.55) | 483 (0.62) | 560 (0.81) | 122 (1.00) |
| **Safety** |  |  |  |  |  |
| MB/CRNMB | 12733 (7.49) | 1114 (5.47) | 5475 (7.53) | 4871 (7.46) | 1273 (10.95) |
| Major bleeding | 6861 (3.90) | 602 (2.88) | 3150 (4.19) | 2482 (3.67) | 627 (5.24) |
| CRNMB | 7229 (4.12) | 623 (2.97) | 2936 (3.89) | 2902 (4.33) | 768 (6.49) |
| Intracranial hemorrhage | 1817 (1.01) | 187 (0.88) | 871 (1.13) | 648 (0.94) | 111 (0.91) |
| Gastrointestinal bleeding | 3630 (2.03) | 310 (1.45) | 1738 (2.26) | 1214 (1.77) | 368 (3.05) |
| Upper gastrointestinal bleeding | 1776 (0.98) | 146 (0.68) | 805 (1.03) | 647 (0.93) | 178 (1.46) |
| Lower gastrointestinal bleeding | 2296 (1.27) | 199 (0.93) | 1165 (1.50) | 708 (1.02) | 224 (1.85) |
| Urogenital bleeding | 2383 (1.32) | 197 (0.92) | 1040 (1.34) | 892 (1.29) | 254 (2.10) |
| Bleeding from other sites | 6944 (3.94) | 573 (2.72) | 2815 (3.71) | 2829 (4.21) | 727 (6.13) |

**B) Reduced dose NOACs**

| **Outcome** | **NOAC**  **(n = 71842)** | **Dabigatran**  **(n = 15482)** | **Rivaroxaban**  **(n = 29718)** | **Apixaban**  **(n = 19664)** | **Edoxaban**  **(n = 6978)** |
| --- | --- | --- | --- | --- | --- |
|  | **Events (per 100 PY)** | **Events (per 100 PY)** | **Events (per 100 PY)** | **Events (per 100 PY)** | **Events (per 100 PY)** |
| **Effectiveness** |  |  |  |  |  |
| Stroke or systemic embolism | 2514 (2.71) | 579 (2.47) | 1142 (2.68) | 649 (2.96) | 144 (3.10) |
| Stroke | 2172 (2.34) | 505 (2.15) | 986 (2.30) | 567 (2.58) | 114 (2.45) |
| Ischemic stroke | 1444 (1.55) | 361 (1.53) | 638 (1.48) | 373 (1.69) | 72 (1.54) |
| Systemic embolism | 398 (0.42) | 89 (0.37) | 181 (0.42) | 92 (0.41) | 36 (0.77) |
| Mortality | 12231 (12.94) | 2126 (8.87) | 5181 (11.92) | 4119 (18.39) | 805 (17.15) |
| Myocardial infarction | 1108 (1.18) | 228 (0.96) | 465 (1.08) | 318 (1.43) | 97 (2.08) |
| **Safety** |  |  |  |  |  |
| MB/CRNMB | 8664 (9.86) | 1842 (8.19) | 4000 (9.97) | 2110 (10.09) | 712 (16.24) |
| Major bleeding | 5169 (5.69) | 1198 (5.19) | 2459 (5.91) | 1134 (5.23) | 378 (8.30) |
| CRNMB | 4476 (4.92) | 856 (3.68) | 2007 (4.81) | 1200 (5.58) | 413 (9.19) |
| Intracranial hemorrhage | 1194 (1.28) | 289 (1.22) | 572 (1.33) | 267 (1.20) | 66 (1.41) |
| Gastrointestinal bleeding | 2853 (3.08) | 692 (2.94) | 1356 (3.19) | 592 (2.69) | 213 (4.61) |
| Upper gastrointestinal bleeding | 1385 (1.48) | 315 (1.32) | 635 (1.47) | 324 (1.46) | 111 (2.38) |
| Lower gastrointestinal bleeding | 1795 (1.92) | 439 (1.85) | 897 (2.09) | 339 (1.53) | 120 (2.58) |
| Urogenital bleeding | 1678 (1.80) | 366 (1.55) | 785 (1.83) | 408 (1.84) | 119 (2.56) |
| Bleeding from other sites | 4546 (4.99) | 862 (3.70) | 2057 (4.92) | 1189 (5.52) | 438 (9.76) |

**eTable 7:** The number of events and crude event rates per 100 person-years of outcomes in subgroup of AF patients treated with **A)** standard dose NOACs and **B)** reduced dose NOACs.

CRNMB: clinically relevant non-major bleeding; MB: major bleeding; NOAC: non-vitamin K antagonist oral anticoagulant; PY: person-year; VKA: vitamin K antagonist.

## eTable 8: Adjusted HRs of outcomes compared between standard or reduced dose NOACs and VKAs after IPTW

**A) Standard dose NOACs vs VKAs**

|  | **NOAC vs VKA** | | **Standard dose**  **dabigatran vs VKA** | | **Standard dose**  **rivaroxaban vs VKA** | | **Standard dose**  **apixaban vs VKA** | | **Standard dose**  **edoxaban vs VKA** | |
| --- | --- | --- | --- | --- | --- | --- | --- | --- | --- | --- |
|  | **HR (95%CI)** | **p-value** | **HR (95%CI)** | **p-value** | **HR (95%CI)** | **p-value** | **HR (95%CI)** | **p-value** | **HR (95%CI)** | **p-value** |
| **Effectiveness** |  |  |  |  |  |  |  |  |  |  |
| Stroke or systemic embolism | 0.64 (0.60-0.68) | <0.001 | 0.63 (0.54-0.74) | <0.001 | 0.70 (0.64-0.76) | <0.001 | 0.61 (0.56-0.67) | <0.001 | 0.54 (0.43-0.67) | <0.001 |
| Stroke | 0.70 (0.65-0.76) | <0.001 | 0.74 (0.62-0.88) | <0.001 | 0.78 (0.71-0.85) | <0.001 | 0.71 (0.64-0.78) | <0.001 | 0.65 (0.50-0.84) | <0.001 |
| Ischemic stroke | 0.62 (0.56-0.68) | <0.001 | 0.77 (0.62-0.95) | 0.016 | 0.70 (0.62-0.78) | <0.001 | 0.63 (0.56-0.71) | <0.001 | 0.60 (0.43-0.84) | 0.003 |
| Systemic embolism | 0.46 (0.40-0.53) | <0.001 | 0.49 (0.35-0.68) | <0.001 | 0.49 (0.41-0.59) | <0.001 | 0.37 (0.31-0.46) | <0.001 | 0.34 (0.22-0.52) | <0.001 |
| All-cause mortality | 0.68 (0.65-0.71) | <0.001 | 0.75 (0.66-0.85) | <0.001 | 0.76 (0.72-0.80) | <0.001 | 0.70 (0.67-0.74) | <0.001 | 0.53 (0.46-0.62) | <0.001 |
| Myocardial infarction | 0.89 (0.79-0.99) | 0.048 | 1.06 (0.81-1.39) | 0.656 | 0.84 (0.72-0.97) | 0.015 | 0.98 (0.84-1.14) | 0.773 | 0.77 (0.56-1.07) | 0.116 |
| **Safety** |  |  |  |  |  |  |  |  |  |  |
| MB/CRNMB | 0.93 (0.90-0.97) | <0.001 | 0.82 (0.75-0.90) | <0.001 | 1.00 (0.95-1.04) | 0.890 | 0.89 (0.84-0.93) | <0.001 | 0.88 (0.78-0.98) | 0.019 |
| Major bleeding | 0.85 (0.81-0.89) | <0.001 | 0.77 (0.68-0.87) | <0.001 | 0.97 (0.92-1.03) | 0.330 | 0.80 (0.75-0.86) | <0.001 | 0.96 (0.82-1.12) | 0.589 |
| CRNMB | 1.01 (0.95-1.06) | 0.800 | 0.91 (0.81-1.02) | 0.119 | 1.00 (0.94-1.07) | 0.959 | 0.94 (0.88-1.00) | 0.062 | 0.82 (0.71-0.94) | 0.005 |
| Intracranial hemorrhage | 0.70 (0.63-0.77) | <0.001 | 0.80 (0.65-1.00) | 0.052 | 0.86 (0.77-0.96) | 0.006 | 0.74 (0.65-0.84) | <0.001 | 0.85 (0.58-1.25) | 0.403 |
| Gastrointestinal bleeding | 1.04 (0.96-1.12) | 0.307 | 0.96 (0.82-1.13) | 0.636 | 1.23 (1.13-1.33) | <0.001 | 0.87 (0.79-0.96) | 0.007 | 1.31 (1.04-1.64) | 0.020 |
| Upper gastrointestinal bleeding | 1.00 (0.90-1.12) | 0.931 | 0.87 (0.68-1.10) | 0.228 | 1.13 (1.00-1.27) | 0.054 | 0.89 (0.78-1.02) | 0.088 | 1.23 (0.90-1.67) | 0.193 |
| Lower gastrointestinal bleeding | 1.02 (0.93-1.12) | 0.683 | 0.96 (0.78-1.18) | 0.683 | 1.28 (1.15-1.42) | <0.001 | 0.81 (0.71-0.92) | <0.001 | 1.35 (1.00-1.82) | 0.052 |
| Urogenital bleeding | 1.04 (0.94-1.14) | 0.477 | 0.90 (0.72-1.12) | 0.340 | 1.15 (1.03-1.29) | 0.011 | 0.93 (0.82-1.05) | 0.242 | 0.91 (0.69-1.20) | 0.496 |
| Bleeding from other sites | 0.90 (0.86-0.95) | <0.001 | 0.74 (0.65-0.84) | <0.001 | 0.89 (0.84-0.95) | <0.001 | 0.85 (0.80-0.91) | <0.001 | 0.74 (0.65-0.85) | <0.001 |

**B) Reduced dose NOACs vs VKAs**

|  | **NOAC vs VKA** | | **Reduced dose**  **dabigatran vs VKA** | | **Reduced dose**  **rivaroxaban vs VKA** | | **Reduced dose**  **apixaban vs VKA** | | **Reduced dose**  **edoxaban vs VKA** | |
| --- | --- | --- | --- | --- | --- | --- | --- | --- | --- | --- |
|  | **HR (95%CI)** | **p-value** | **HR (95%CI)** | **p-value** | **HR (95%CI)** | **p-value** | **HR (95%CI)** | **p-value** | **HR (95%CI)** | **p-value** |
| **Effectiveness** |  |  |  |  |  |  |  |  |  |  |
| Stroke or systemic embolism | 0.79 (0.73-0.84) | <0.001 | 0.81 (0.70-0.93) | 0.003 | 0.80 (0.73-0.87) | <0.001 | 0.77 (0.68-0.88) | <0.001 | 0.94 (0.68-1.30) | 0.712 |
| Stroke | 0.84 (0.77-0.91) | <0.001 | 0.86 (0.74-0.99) | 0.044 | 0.87 (0.79-0.96) | 0.005 | 0.87 (0.76-1.01) | 0.069 | 0.80 (0.57-1.11) | 0.182 |
| Ischemic stroke | 0.79 (0.72-0.87) | <0.001 | 0.86 (0.72-1.03) | 0.098 | 0.82 (0.73-0.92) | <0.001 | 0.82 (0.68-0.98) | 0.028 | 0.76 (0.52-1.09) | 0.137 |
| Systemic embolism | 0.63 (0.54-0.74) | <0.001 | 0.70 (0.49-0.99) | 0.048 | 0.61 (0.49-0.75) | <0.001 | 0.56 (0.40-0.77) | <0.001 | 1.28 (0.70-2.33) | 0.426 |
| All-cause mortality | 0.97 (0.93-1.01) | 0.173 | 0.84 (0.79-0.90) | <0.001 | 1.03 (0.99-1.08) | 0.153 | 1.00 (0.95-1.06) | 0.887 | 0.84 (0.73-0.97) | 0.016 |
| Myocardial infarction | 1.12 (0.96-1.30) | 0.162 | 1.24 (0.98-1.57) | 0.070 | 1.16 (1.00-1.34) | 0.052 | 1.25 (0.93-1.69) | 0.128 | 1.36 (0.89-2.08) | 0.153 |
| **Safety** |  |  |  |  |  |  |  |  |  |  |
| MB/CRNMB | 1.02 (0.98-1.06) | 0.381 | 0.95 (0.88-1.02) | 0.167 | 1.08 (1.02-1.13) | 0.003 | 0.92 (0.85-0.99) | 0.035 | 1.00 (0.87-1.15) | 0.998 |
| Major bleeding | 0.99 (0.94-1.05) | 0.808 | 1.02 (0.93-1.12) | 0.672 | 1.09 (1.03-1.16) | 0.006 | 0.81 (0.73-0.89) | <0.001 | 0.98 (0.81-1.19) | 0.871 |
| CRNMB | 1.04 (0.99-1.11) | 0.136 | 0.89 (0.80-0.99) | 0.040 | 1.06 (0.99-1.13) | 0.105 | 1.02 (0.92-1.13) | 0.678 | 1.04 (0.86-1.24) | 0.699 |
| Intracranial hemorrhage | 0.82 (0.74-0.91) | <0.001 | 0.98 (0.80-1.20) | 0.850 | 0.91 (0.81-1.04) | 0.160 | 0.74 (0.60-0.91) | 0.004 | 1.47 (0.89-2.43) | 0.135 |
| Gastrointestinal bleeding | 1.19 (1.10-1.28) | <0.001 | 1.22 (1.08-1.38) | 0.002 | 1.31 (1.20-1.43) | <0.001 | 0.95 (0.82-1.11) | 0.514 | 1.10 (0.84-1.44) | 0.501 |
| Upper gastrointestinal bleeding | 1.18 (1.05-1.31) | 0.005 | 1.06 (0.89-1.28) | 0.505 | 1.20 (1.05-1.36) | 0.005 | 1.00 (0.81-1.24) | 0.979 | 1.42 (0.98-2.07) | 0.066 |
| Lower gastrointestinal bleeding | 1.15 (1.05-1.27) | 0.005 | 1.25 (1.07-1.47) | 0.005 | 1.37 (1.23-1.53) | <0.001 | 0.91 (0.75-1.12) | 0.389 | 0.91 (0.64-1.29) | 0.605 |
| Urogenital bleeding | 1.24 (1.12-1.37) | <0.001 | 1.01 (0.86-1.19) | 0.888 | 1.33 (1.18-1.50) | <0.001 | 1.15 (0.97-1.36) | 0.102 | 0.98 (0.69-1.40) | 0.922 |
| Bleeding from other sites | 0.94 (0.89-0.99) | 0.030 | 0.81 (0.72-0.90) | <0.001 | 0.96 (0.90-1.02) | 0.189 | 0.90 (0.81-0.99) | 0.036 | 0.97 (0.81-1.15) | 0.717 |

**eTable 8:** Adjusted hazard ratios of outcomes compared between **A)** (individual) standard dose NOACs and VKAs and B**)** (individual) reduced dose NOACs and VKAs after IPTW.

CI: confidence interval; CRNMB: clinically relevant non-major bleeding; HR: hazard ratio; IPTW: inverse probability of treatment weighting; MB: major bleeding; NOAC: non-vitamin K antagonist oral anticoagulant; VKA: vitamin K antagonist; vs: versus.

## eTable 9: Adjusted HRs of outcomes compared between individual standard or reduced dose NOAC types after IPTW

**A) Standard dose NOACs**

|  | **Standard dose**  **Dabigatran vs Rivaroxaban** | | **Standard dose**  **Apixaban vs Rivaroxaban** | | **Standard dose**  **Edoxaban vs Rivaroxaban** | | **Standard dose**  **Apixaban vs Dabigatran** | | **Standard dose**  **Dabigatran vs Edoxaban** | | **Standard dose**  **Apixaban vs Edoxaban** | |
| --- | --- | --- | --- | --- | --- | --- | --- | --- | --- | --- | --- | --- |
|  | **HR (95%CI)** | **p-value** | **HR (95%CI)** | **p-value** | **HR (95%CI)** | **p-value** | **HR (95%CI)** | **p-value** | **HR (95%CI)** | **p-value** | **HR (95%CI)** | **p-value** |
| **Effectiveness** |  |  |  |  |  |  |  |  |  |  |  |  |
| Stroke or systemic embolism | 0.88 (0.77-1.01) | 0.062 | 0.95 (0.87-1.03) | 0.203 | 0.91 (0.76-1.10) | 0.343 | 1.04 (0.89-1.21) | 0.644 | 1.00 (0.73-1.35) | 0.980 | 0.95 (0.81-1.12) | 0.551 |
| Stroke | 0.86 (0.75-1.00) | 0.053 | 0.96 (0.87-1.05) | 0.380 | 0.93 (0.75-1.14) | 0.467 | 1.09 (0.91-1.29) | 0.346 | 1.07 (0.77-1.50) | 0.686 | 1.00 (0.84-1.20) | 0.987 |
| Ischemic stroke | 0.97 (0.81-1.17) | 0.754 | 0.95 (0.84-1.08) | 0.459 | 0.98 (0.74-1.30) | 0.906 | 0.97 (0.78-1.20) | 0.761 | 1.43 (0.95-2.16) | 0.090 | 1.06 (0.83-1.34) | 0.648 |
| Systemic embolism | 1.07 (0.81-1.43) | 0.624 | 0.89 (0.73-1.10) | 0.301 | 0.87 (0.59-1.28) | 0.475 | 0.79 (0.55-1.12) | 0.179 | 0.73 (0.37-1.43) | 0.363 | 0.74 (0.52-1.07) | 0.112 |
| All-cause mortality | 0.83 (0.75-0.91) | <0.001 | 0.97 (0.93-1.03) | 0.320 | 0.84 (0.75-0.95) | 0.005 | 1.09 (0.97-1.22) | 0.160 | 0.95 (0.75-1.20) | 0.671 | 1.18 (1.07-1.31) | <0.001 |
| Myocardial infarction | 1.07 (0.86-1.33) | 0.556 | 1.09 (0.95-1.24) | 0.223 | 0.91 (0.70-1.19) | 0.496 | 1.01 (0.79-1.29) | 0.915 | 0.94 (0.60-1.48) | 0.795 | 1.06 (0.84-1.34) | 0.615 |
| **Safety** |  |  |  |  |  |  |  |  |  |  |  |  |
| MB/CRNMB | 0.83 (0.78-0.89) | <0.001 | 0.87 (0.84-0.91) | <0.001 | 0.95 (0.87-1.03) | 0.194 | 1.03 (0.95-1.12) | 0.452 | 0.89 (0.77-1.03) | 0.117 | 0.86 (0.80-0.92) | <0.001 |
| Major bleeding | 0.80 (0.72-0.87) | <0.001 | 0.79 (0.75-0.84) | <0.001 | 0.95 (0.84-1.07) | 0.386 | 0.99 (0.88-1.11) | 0.830 | 0.94 (0.76-1.16) | 0.567 | 0.80 (0.72-0.89) | <0.001 |
| CRNMB | 0.87 (0.80-0.96) | 0.005 | 0.94 (0.89-0.99) | 0.040 | 0.95 (0.86-1.06) | 0.355 | 1.08 (0.97-1.20) | 0.180 | 0.87 (0.72-1.04) | 0.128 | 0.90 (0.82-0.99) | 0.032 |
| Intracranial hemorrhage | 0.86 (0.73-1.02) | 0.087 | 0.87 (0.78-0.98) | 0.020 | 1.02 (0.77-1.35) | 0.910 | 0.99 (0.78-1.25) | 0.926 | 1.10 (0.68-1.77) | 0.693 | 1.09 (0.86-1.39) | 0.475 |
| Gastrointestinal bleeding | 0.79 (0.69-0.90) | <0.001 | 0.68 (0.63-0.73) | <0.001 | 0.90 (0.78-1.05) | 0.185 | 0.88 (0.75-1.02) | 0.093 | 0.91 (0.69-1.21) | 0.524 | 0.67 (0.58-0.77) | <0.001 |
| Upper gastrointestinal bleeding | 0.81 (0.67-0.98) | 0.033 | 0.75 (0.67-0.84) | <0.001 | 0.81 (0.66-1.01) | 0.057 | 0.91 (0.74-1.13) | 0.399 | 1.08 (0.74-1.57) | 0.708 | 0.78 (0.63-0.95) | 0.014 |
| Lower gastrointestinal bleeding | 0.74 (0.63-0.87) | <0.001 | 0.60 (0.54-0.66) | <0.001 | 0.92 (0.76-1.12) | 0.403 | 0.84 (0.69-1.02) | 0.081 | 0.83 (0.57-1.21) | 0.339 | 0.59 (0.48-0.71) | <0.001 |
| Urogenital bleeding | 0.79 (0.67-0.94) | 0.007 | 0.84 (0.76-0.93) | <0.001 | 0.90 (0.75-1.08) | 0.274 | 1.08 (0.88-1.32) | 0.461 | 0.84 (0.59-1.21) | 0.359 | 0.74 (0.62-0.88) | <0.001 |
| Bleeding from other sites | 0.83 (0.75-0.92) | <0.001 | 0.96 (0.90-1.01) | 0.113 | 0.94 (0.84-1.04) | 0.234 | 1.09 (0.98-1.22) | 0.105 | 0.88 (0.73-1.06) | 0.173 | 0.92 (0.84-1.02) | 0.114 |

**B) Reduced dose NOACs**

|  | **Reduced dose**  **Dabigatran vs Rivaroxaban** | | **Reduced dose**  **Apixaban vs Rivaroxaban** | | **Reduced dose**  **Edoxaban vs Rivaroxaban** | | **Reduced dose**  **Apixaban vs Dabigatran** | | **Reduced dose**  **Dabigatran vs Edoxaban** | | **Reduced dose**  **Apixaban vs Edoxaban** | |
| --- | --- | --- | --- | --- | --- | --- | --- | --- | --- | --- | --- | --- |
|  | **HR (95%CI)** | **p-value** | **HR (95%CI)** | **p-value** | **HR (95%CI)** | **p-value** | **HR (95%CI)** | **p-value** | **HR (95%CI)** | **p-value** | **HR (95%CI)** | **p-value** |
| **Effectiveness** |  |  |  |  |  |  |  |  |  |  |  |  |
| Stroke or systemic embolism | 0.91 (0.82-1.01) | 0.072 | 0.99 (0.89-1.10) | 0.851 | 1.06 (0.83-1.36) | 0.629 | 1.14 (0.99-1.30) | 0.066 | 0.94 (0.72-1.25) | 0.687 | 1.02 (0.82-1.27) | 0.839 |
| Stroke | 0.90 (0.81-1.00) | 0.061 | 1.01 (0.90-1.13) | 0.909 | 1.03 (0.78-1.36) | 0.842 | 1.13 (0.98-1.31) | 0.090 | 1.00 (0.73-1.35) | 0.980 | 1.00 (0.78-1.28) | 0.992 |
| Ischemic stroke | 1.00 (0.88-1.14) | 0.995 | 1.04 (0.90-1.21) | 0.553 | 1.03 (0.72-1.45) | 0.887 | 1.06 (0.89-1.27) | 0.507 | 1.02 (0.70-1.49) | 0.911 | 1.09 (0.80-1.47) | 0.594 |
| Systemic embolism | 0.98 (0.75-1.27) | 0.857 | 0.90 (0.68-1.18) | 0.436 | 1.23 (0.74-2.04) | 0.432 | 1.07 (0.75-1.53) | 0.702 | 0.89 (0.51-1.56) | 0.687 | 1.01 (0.65-1.57) | 0.981 |
| All-cause mortality | 0.83 (0.79-0.88) | <0.001 | 1.14 (1.09-1.19) | <0.001 | 0.94 (0.85-1.04) | 0.239 | 1.40 (1.31-1.49) | <0.001 | 0.79 (0.69-0.91) | <0.001 | 1.23 (1.13-1.34) | <0.001 |
| Myocardial infarction | 0.96 (0.82-1.14) | 0.653 | 1.07 (0.92-1.26) | 0.384 | 0.92 (0.68-1.24) | 0.586 | 1.17 (0.96-1.44) | 0.128 | 0.98 (0.68-1.40) | 0.903 | 0.96 (0.73-1.26) | 0.769 |
| **Safety** |  |  |  |  |  |  |  |  |  |  |  |  |
| MB/CRNMB | 0.89 (0.84-0.94) | <0.001 | 0.88 (0.83-0.93) | <0.001 | 1.01 (0.91-1.13) | 0.857 | 0.92 (0.86-0.99) | 0.033 | 0.93 (0.81-1.07) | 0.303 | 0.87 (0.79-0.97) | 0.009 |
| Major bleeding | 0.95 (0.89-1.02) | 0.183 | 0.79 (0.73-0.85) | <0.001 | 0.99 (0.85-1.15) | 0.892 | 0.79 (0.72-0.87) | <0.001 | 1.03 (0.85-1.25) | 0.755 | 0.78 (0.68-0.90) | <0.001 |
| CRNMB | 0.83 (0.76-0.90) | <0.001 | 0.97 (0.90-1.05) | 0.483 | 0.97 (0.85-1.12) | 0.715 | 1.08 (0.97-1.19) | 0.162 | 0.80 (0.67-0.97) | 0.023 | 0.95 (0.83-1.09) | 0.469 |
| Intracranial hemorrhage | 0.98 (0.85-1.14) | 0.805 | 0.94 (0.80-1.10) | 0.436 | 1.43 (0.97-2.10) | 0.068 | 0.94 (0.77-1.15) | 0.560 | 0.80 (0.50-1.28) | 0.361 | 0.99 (0.72-1.37) | 0.968 |
| Gastrointestinal bleeding | 0.99 (0.90-1.09) | 0.860 | 0.72 (0.65-0.80) | <0.001 | 0.87 (0.72-1.06) | 0.161 | 0.70 (0.62-0.80) | <0.001 | 1.11 (0.86-1.43) | 0.406 | 0.71 (0.59-0.87) | <0.001 |
| Upper gastrointestinal bleeding | 0.94 (0.82-1.08) | 0.373 | 0.81 (0.70-0.94) | 0.006 | 0.86 (0.65-1.12) | 0.252 | 0.74 (0.62-0.89) | <0.001 | 1.18 (0.83-1.69) | 0.361 | 0.80 (0.62-1.04) | 0.095 |
| Lower gastrointestinal bleeding | 0.97 (0.86-1.09) | 0.565 | 0.65 (0.56-0.74) | <0.001 | 0.77 (0.60-0.99) | 0.047 | 0.72 (0.61-0.85) | <0.001 | 1.11 (0.81-1.54) | 0.508 | 0.66 (0.51-0.85) | 0.002 |
| Urogenital bleeding | 0.85 (0.74-0.96) | 0.011 | 0.89 (0.78-1.01) | 0.076 | 0.73 (0.57-0.94) | 0.015 | 0.90 (0.77-1.07) | 0.234 | 0.90 (0.64-1.25) | 0.521 | 0.97 (0.76-1.23) | 0.775 |
| Bleeding from other sites | 0.84 (0.77-0.91) | <0.001 | 0.92 (0.85-0.99) | 0.046 | 1.04 (0.90-1.19) | 0.610 | 1.02 (0.92-1.13) | 0.772 | 0.85 (0.70-1.02) | 0.078 | 0.88 (0.78-1.01) | 0.064 |

**eTable 9:** Adjusted hazard ratios of outcomes compared between **A)** individual standard dose NOAC types, and **B)** individual reduced dose NOAC types after IPTW.

CI: confidence interval; CRNMB: clinically relevant non-major bleeding; HR: hazard ratio; IPTW: inverse probability of treatment weighting; MB: major bleeding; NOAC: non-vitamin K antagonist oral anticoagulant; vs: versus.

eTable 10: Adjusted HRs of outcomes after 1:1 PSM

**A)**

|  | **NOAC vs VKA**  **(n = 55564 per group)** | | **Dabigatran vs VKA**  **(n = 23180 per group)** | | **Rivaroxaban vs VKA**  **(n = 45779 per group)** | | **Apixaban vs VKA**  **(n = 34362 per group)** | | **Edoxaban vs VKA**  **(n = 8496 per group)** | |
| --- | --- | --- | --- | --- | --- | --- | --- | --- | --- | --- |
|  | **HR (95%CI)** | **p-value** | **HR (95%CI)** | **p-value** | **HR (95%CI)** | **p-value** | **HR (95%CI)** | **p-value** | **HR (95%CI)** | **p-value** |
| **Effectiveness** |  |  |  |  |  |  |  |  |  |  |
| Stroke or systemic embolism | 0.72 (0.68-0.78) | <0.001 | 0.64 (0.58-0.71) | <0.001 | 0.75 (0.70-0.81) | <0.001 | 0.63 (0.58-0.69) | <0.001 | 0.56 (0.45-0.70) | <0.001 |
| Stroke | 0.81 (0.75-0.87) | <0.001 | 0.68 (0.61-0.76) | <0.001 | 0.81 (0.75-0.88) | <0.001 | 0.72 (0.65-0.79) | <0.001 | 0.67 (0.52-0.86) | 0.002 |
| Ischemic stroke | 0.74 (0.67-0.81) | <0.001 | 0.70 (0.62-0.80) | <0.001 | 0.75 (0.68-0.83) | <0.001 | 0.65 (0.57-0.73) | <0.001 | 0.60 (0.44-0.83) | 0.002 |
| Systemic embolism | 0.52 (0.45-0.60) | <0.001 | 0.53 (0.43-0.66) | <0.001 | 0.58 (0.49-0.68) | <0.001 | 0.41 (0.34-0.50) | <0.001 | 0.36 (0.24-0.56) | <0.001 |
| All-cause mortality | 0.86 (0.83-0.90) | <0.001 | 0.68 (0.64-0.72) | <0.001 | 0.83 (0.80-0.87) | <0.001 | 0.78 (0.74-0.82) | <0.001 | 0.67 (0.60-0.76) | <0.001 |
| Myocardial infarction | 1.07 (0.95-1.20) | 0.275 | 0.93 (0.78-1.10) | 0.380 | 0.97 (0.86-1.10) | 0.647 | 1.03 (0.89-1.20) | 0.649 | 0.96 (0.70-1.32) | 0.813 |
| **Safety** |  |  |  |  |  |  |  |  |  |  |
| MB/CRNMB | 0.99 (0.96-1.03) | 0.742 | 0.86 (0.81-0.91) | <0.001 | 1.01 (0.97-1.05) | 0.683 | 0.85 (0.81-0.89) | <0.001 | 0.91 (0.82-1.01) | 0.088 |
| Major bleeding | 0.92 (0.87-0.97) | 0.002 | 0.87 (0.81-0.94) | <0.001 | 0.99 (0.94-1.04) | 0.678 | 0.75 (0.70-0.80) | <0.001 | 1.04 (0.89-1.21) | 0.619 |
| CRNMB | 1.06 (1.01-1.12) | 0.036 | 0.82 (0.75-0.89) | <0.001 | 1.01 (0.96-1.08) | 0.616 | 0.93 (0.87-0.99) | 0.024 | 0.80 (0.70-0.91) | <0.001 |
| Intracranial hemorrhage | 0.74 (0.67-0.81) | <0.001 | 0.77 (0.67-0.88) | <0.001 | 0.86 (0.78-0.96) | 0.006 | 0.67 (0.59-0.77) | <0.001 | 1.01 (0.69-1.48) | 0.969 |
| Gastrointestinal bleeding | 1.14 (1.06-1.23) | <0.001 | 1.09 (0.98-1.21) | 0.125 | 1.21 (1.11-1.30) | <0.001 | 0.84 (0.76-0.93) | <0.001 | 1.29 (1.04-1.59) | 0.020 |
| Upper gastrointestinal bleeding | 1.10 (0.99-1.23) | 0.071 | 0.95 (0.82-1.11) | 0.526 | 1.13 (1.01-1.27) | 0.037 | 0.88 (0.77-1.00) | 0.056 | 1.25 (0.93-1.68) | 0.138 |
| Lower gastrointestinal bleeding | 1.10 (1.01-1.21) | 0.047 | 1.09 (0.95-1.25) | 0.206 | 1.25 (1.14-1.38) | <0.001 | 0.77 (0.68-0.87) | <0.001 | 1.31 (0.99-1.73) | 0.063 |
| Urogenital bleeding | 1.14 (1.04-1.26) | 0.007 | 0.92 (0.80-1.06) | 0.256 | 1.18 (1.07-1.31) | <0.001 | 0.92 (0.81-1.04) | 0.195 | 0.98 (0.75-1.28) | 0.873 |
| Bleeding from other sites | 0.94 (0.90-0.99) | 0.027 | 0.75 (0.69-0.81) | <0.001 | 0.91 (0.86-0.97) | 0.002 | 0.83 (0.78-0.88) | <0.001 | 0.75 (0.66-0.86) | <0.001 |

**B)**

|  | **Dabigatran vs Rivaroxaban**  **(n = 28043 per group)** | | **Apixaban vs Rivaroxaban**  **(n = 56257 per group)** | | **Edoxaban vs Rivaroxaban**  **(n = 17282 per group)** | | **Apixaban vs Dabigatran**  **(n = 22287 per group)** | | **Dabigatran vs Edoxaban**  **(n = 7692 per group)** | | **Apixaban vs Edoxaban**  **(n = 22348 per group)** | |
| --- | --- | --- | --- | --- | --- | --- | --- | --- | --- | --- | --- | --- |
|  | **HR (95%CI)** | **p-value** | **HR (95%CI)** | **p-value** | **HR (95%CI)** | **p-value** | **HR (95%CI)** | **p-value** | **HR (95%CI)** | **p-value** | **HR (95%CI)** | **p-value** |
| **Effectiveness** |  |  |  |  |  |  |  |  |  |  |  |  |
| Stroke or systemic embolism | 0.87 (0.80-0.95) | 0.002 | 0.91 (0.85-0.98) | 0.010 | 0.94 (0.80-1.10) | 0.447 | 1.04 (0.94-1.17) | 0.439 | 1.02 (0.82-1.27) | 0.857 | 0.97 (0.84-1.11) | 0.616 |
| Stroke | 0.86 (0.78-0.94) | <0.001 | 0.94 (0.87-1.01) | 0.077 | 0.96 (0.80-1.14) | 0.622 | 1.05 (0.93-1.18) | 0.411 | 1.01 (0.80-1.29) | 0.915 | 1.00 (0.85-1.16) | 0.965 |
| Ischemic stroke | 0.95 (0.84-1.07) | 0.391 | 0.94 (0.85-1.03) | 0.184 | 0.94 (0.75-1.19) | 0.615 | 0.95 (0.81-1.10) | 0.466 | 1.19 (0.88-1.60) | 0.262 | 1.04 (0.85-1.27) | 0.708 |
| Systemic embolism | 1.02 (0.82-1.27) | 0.849 | 0.82 (0.69-0.97) | 0.024 | 0.90 (0.65-1.25) | 0.532 | 0.95 (0.73-1.24) | 0.709 | 1.29 (0.77-2.17) | 0.333 | 0.81 (0.60-1.09) | 0.162 |
| All-cause mortality | 0.84 (0.80-0.89) | <0.001 | 0.98 (0.95-1.01) | 0.207 | 0.87 (0.80-0.94) | <0.001 | 1.09 (1.03-1.16) | 0.003 | 1.00 (0.88-1.15) | 0.968 | 1.21 (1.13-1.30) | <0.001 |
| Myocardial infarction | 0.98 (0.84-1.13) | 0.774 | 1.06 (0.96-1.17) | 0.264 | 0.91 (0.74-1.13) | 0.391 | 1.12 (0.95-1.33) | 0.172 | 1.13 (0.82-1.57) | 0.447 | 0.97 (0.80-1.17) | 0.722 |
| **Safety** |  |  |  |  |  |  |  |  |  |  |  |  |
| MB/CRNMB | 0.84 (0.80-0.88) | <0.001 | 0.86 (0.83-0.89) | <0.001 | 0.95 (0.88-1.01) | 0.117 | 0.97 (0.92-1.03) | 0.392 | 0.89 (0.80-0.99) | 0.042 | 0.89 (0.84-0.95) | <0.001 |
| Major bleeding | 0.87 (0.82-0.92) | <0.001 | 0.78 (0.74-0.82) | <0.001 | 0.93 (0.85-1.03) | 0.180 | 0.84 (0.78-0.92) | <0.001 | 0.95 (0.82-1.11) | 0.525 | 0.81 (0.74-0.89) | <0.001 |
| CRNMB | 0.82 (0.77-0.88) | <0.001 | 0.94 (0.90-0.98) | 0.009 | 0.94 (0.86-1.03) | 0.211 | 1.08 (1.00-1.17) | 0.050 | 0.84 (0.73-0.97) | 0.019 | 0.94 (0.87-1.02) | 0.153 |
| Intracranial hemorrhage | 0.86 (0.76-0.97) | 0.012 | 0.89 (0.81-0.98) | 0.022 | 1.07 (0.84-1.36) | 0.589 | 0.97 (0.82-1.14) | 0.703 | 0.96 (0.67-1.37) | 0.807 | 1.10 (0.90-1.35) | 0.355 |
| Gastrointestinal bleeding | 0.88 (0.81-0.96) | 0.004 | 0.68 (0.63-0.72) | <0.001 | 0.88 (0.77-0.99) | 0.045 | 0.72 (0.64-0.80) | <0.001 | 0.94 (0.77-1.15) | 0.558 | 0.71 (0.62-0.80) | <0.001 |
| Upper gastrointestinal bleeding | 0.85 (0.75-0.97) | 0.012 | 0.75 (0.68-0.82) | <0.001 | 0.83 (0.70-0.99) | 0.041 | 0.74 (0.64-0.87) | <0.001 | 1.03 (0.78-1.36) | 0.849 | 0.80 (0.67-0.95) | 0.011 |
| Lower gastrointestinal bleeding | 0.88 (0.79-0.98) | 0.019 | 0.61 (0.56-0.66) | <0.001 | 0.85 (0.72-1.00) | 0.056 | 0.71 (0.61-0.82) | <0.001 | 0.89 (0.69-1.15) | 0.366 | 0.64 (0.54-0.76) | <0.001 |
| Urogenital bleeding | 0.80 (0.72-0.90) | <0.001 | 0.84 (0.78-0.91) | <0.001 | 0.84 (0.72-0.99) | 0.034 | 0.95 (0.83-1.08) | 0.435 | 0.81 (0.62-1.05) | 0.105 | 0.83 (0.71-0.96) | 0.015 |
| Bleeding from other sites | 0.82 (0.77-0.88) | <0.001 | 0.93 (0.89-0.97) | 0.002 | 0.95 (0.87-1.04) | 0.264 | 1.08 (0.99-1.17) | 0.076 | 0.87 (0.75-1.00) | 0.057 | 0.93 (0.86-1.01) | 0.103 |

**eTable 10:** Adjusted hazard ratios of outcomes compared between **A)** (individual) NOACs and VKAs, and **B)** compared between individual NOAC types after 1:1 PSM.

CI: confidence interval; CRNMB: clinically relevant non-major bleeding; HR: hazard ratio; MB: major bleeding; NOAC: non-vitamin K antagonist oral anticoagulant; PSM: propensity score matching; VKA: vitamin K antagonist; vs: versus.

eTable 11: Adjusted HRs of outcomes after IPTW (intention-to-treat analysis)

**A)**

|  | **NOAC vs VKA** | | **Dabigatran vs VKA** | | **Rivaroxaban vs VKA** | | **Apixaban vs VKA** | | **Edoxaban vs VKA** | |
| --- | --- | --- | --- | --- | --- | --- | --- | --- | --- | --- |
|  | **HR (95%CI)** | **p-value** | **HR (95%CI)** | **p-value** | **HR (95%CI)** | **p-value** | **HR (95%CI)** | **p-value** | **HR (95%CI)** | **p-value** |
| **Effectiveness** |  |  |  |  |  |  |  |  |  |  |
| Stroke or systemic embolism | 0.84 (0.80-0.87) | <0.001 | 0.90 (0.85-0.96) | 0.002 | 0.89 (0.85-0.93) | <0.001 | 0.84 (0.79-0.89) | <0.001 | 0.75 (0.65-0.88) | <0.001 |
| Stroke | 0.88 (0.84-0.92) | <0.001 | 0.95 (0.89-1.02) | 0.166 | 0.95 (0.90-0.99) | 0.031 | 0.91 (0.85-0.97) | 0.003 | 0.83 (0.70-1.00) | 0.051 |
| Ischemic stroke | 0.84 (0.79-0.88) | <0.001 | 0.98 (0.90-1.07) | 0.715 | 0.91 (0.86-0.97) | 0.004 | 0.84 (0.78-0.91) | <0.001 | 0.75 (0.60-0.94) | 0.012 |
| Systemic embolism | 0.72 (0.66-0.79) | <0.001 | 0.81 (0.70-0.93) | 0.004 | 0.74 (0.67-0.82) | <0.001 | 0.64 (0.57-0.73) | <0.001 | 0.58 (0.43-0.77) | <0.001 |
| All-cause mortality | 0.88 (0.86-0.90) | <0.001 | 0.96 (0.93-0.99) | 0.022 | 0.95 (0.93-0.98) | <0.001 | 0.93 (0.90-0.95) | <0.001 | 0.80 (0.73-0.87) | <0.001 |
| Myocardial infarction | 1.01 (0.95-1.08) | 0.702 | 1.09 (0.98-1.21) | 0.121 | 1.03 (0.95-1.11) | 0.511 | 1.13 (1.03-1.23) | 0.010 | 0.97 (0.77-1.21) | 0.761 |
| **Safety** |  |  |  |  |  |  |  |  |  |  |
| MB/CRNMB | 1.04 (1.02-1.07) | <0.001 | 1.04 (1.01-1.08) | 0.044 | 1.09 (1.06-1.12) | <0.001 | 1.02 (0.99-1.06) | 0.166 | 1.05 (0.97-1.13) | 0.249 |
| Major bleeding | 0.98 (0.95-1.01) | 0.112 | 1.06 (1.01-1.11) | 0.021 | 1.06 (1.02-1.09) | <0.001 | 0.93 (0.89-0.97) | <0.001 | 1.07 (0.96-1.19) | 0.232 |
| CRNMB | 1.09 (1.06-1.13) | <0.001 | 1.00 (0.95-1.06) | 0.884 | 1.11 (1.07-1.15) | <0.001 | 1.10 (1.05-1.14) | <0.001 | 1.03 (0.93-1.14) | 0.584 |
| Intracranial hemorrhage | 0.84 (0.80-0.90) | <0.001 | 1.00 (0.91-1.09) | 0.935 | 0.94 (0.88-1.01) | 0.075 | 0.87 (0.79-0.94) | <0.001 | 1.00 (0.78-1.29) | 0.988 |
| Gastrointestinal bleeding | 1.08 (1.04-1.13) | <0.001 | 1.21 (1.13-1.29) | <0.001 | 1.19 (1.13-1.24) | <0.001 | 0.93 (0.88-0.99) | 0.027 | 1.30 (1.11-1.52) | <0.001 |
| Upper gastrointestinal bleeding | 1.07 (1.01-1.13) | 0.026 | 1.10 (1.01-1.21) | 0.035 | 1.12 (1.05-1.20) | <0.001 | 0.97 (0.90-1.05) | 0.453 | 1.27 (1.03-1.56) | 0.027 |
| Lower gastrointestinal bleeding | 1.11 (1.05-1.17) | <0.001 | 1.24 (1.14-1.35) | <0.001 | 1.28 (1.21-1.36) | <0.001 | 0.92 (0.85-0.99) | 0.047 | 1.38 (1.12-1.70) | 0.003 |
| Urogenital bleeding | 1.19 (1.12-1.26) | <0.001 | 1.18 (1.08-1.29) | <0.001 | 1.26 (1.19-1.35) | <0.001 | 1.16 (1.08-1.25) | <0.001 | 1.07 (0.87-1.30) | 0.539 |
| Bleeding from other sites | 1.02 (0.99-1.05) | 0.173 | 0.95 (0.90-0.99) | 0.039 | 1.04 (1.01-1.07) | 0.046 | 1.02 (0.98-1.06) | 0.370 | 0.96 (0.87-1.06) | 0.422 |

**B)**

|  | **Dabigatran vs Rivaroxaban** | | **Apixaban vs Rivaroxaban** | | **Edoxaban vs Rivaroxaban** | | **Apixaban vs Dabigatran** | | **Dabigatran vs Edoxaban** | | **Apixaban vs Edoxaban** | |
| --- | --- | --- | --- | --- | --- | --- | --- | --- | --- | --- | --- | --- |
|  | **HR (95%CI)** | **p-value** | **HR (95%CI)** | **p-value** | **HR (95%CI)** | **p-value** | **HR (95%CI)** | **p-value** | **HR (95%CI)** | **p-value** | **HR (95%CI)** | **p-value** |
| **Effectiveness** |  |  |  |  |  |  |  |  |  |  |  |  |
| Stroke or systemic embolism | 0.97 (0.91-1.02) | 0.225 | 0.96 (0.91-1.01) | 0.095 | 0.99 (0.87-1.12) | 0.846 | 0.98 (0.92-1.05) | 0.646 | 1.03 (0.88-1.22) | 0.691 | 0.94 (0.84-1.05) | 0.264 |
| Stroke | 0.95 (0.89-1.01) | 0.093 | 0.96 (0.91-1.02) | 0.184 | 1.01 (0.88-1.17) | 0.885 | 1.00 (0.93-1.08) | 0.956 | 1.03 (0.86-1.23) | 0.758 | 0.94 (0.83-1.07) | 0.372 |
| Ischemic stroke | 1.03 (0.95-1.11) | 0.495 | 0.95 (0.89-1.02) | 0.167 | 1.07 (0.89-1.29) | 0.456 | 0.92 (0.83-1.01) | 0.065 | 1.16 (0.93-1.45) | 0.182 | 0.94 (0.80-1.11) | 0.482 |
| Systemic embolism | 1.10 (0.97-1.26) | 0.152 | 0.90 (0.79-1.02) | 0.087 | 0.90 (0.69-1.18) | 0.445 | 0.82 (0.70-0.97) | 0.019 | 1.13 (0.81-1.59) | 0.468 | 0.86 (0.67-1.10) | 0.231 |
| All-cause mortality | 0.94 (0.91-0.96) | <0.001 | 0.96 (0.94-0.98) | <0.001 | 0.92 (0.86-0.98) | 0.011 | 1.06 (1.03-1.10) | <0.001 | 0.97 (0.89-1.06) | 0.508 | 1.08 (1.02-1.15) | 0.005 |
| Myocardial infarction | 0.99 (0.90-1.09) | 0.843 | 0.99 (0.92-1.07) | 0.794 | 0.91 (0.76-1.09) | 0.292 | 1.05 (0.94-1.18) | 0.362 | 1.15 (0.92-1.45) | 0.221 | 1.02 (0.87-1.20) | 0.807 |
| **Safety** |  |  |  |  |  |  |  |  |  |  |  |  |
| MB/CRNMB | 0.92 (0.89-0.95) | <0.001 | 0.90 (0.87-0.92) | <0.001 | 0.96 (0.90-1.01) | 0.131 | 0.98 (0.95-1.02) | 0.425 | 0.92 (0.85-0.99) | 0.047 | 0.87 (0.82-0.92) | <0.001 |
| Major bleeding | 0.96 (0.92-0.99) | 0.038 | 0.83 (0.80-0.86) | <0.001 | 0.95 (0.88-1.03) | 0.236 | 0.88 (0.83-0.92) | <0.001 | 0.99 (0.88-1.11) | 0.844 | 0.80 (0.74-0.86) | <0.001 |
| CRNMB | 0.87 (0.84-0.91) | <0.001 | 0.96 (0.92-0.99) | 0.016 | 0.94 (0.88-1.02) | 0.141 | 1.09 (1.03-1.14) | 0.002 | 0.87 (0.78-0.97) | 0.013 | 0.93 (0.87-0.99) | 0.041 |
| Intracranial hemorrhage | 0.99 (0.91-1.07) | 0.768 | 0.92 (0.85-0.99) | 0.023 | 1.14 (0.93-1.39) | 0.199 | 0.96 (0.87-1.07) | 0.504 | 0.90 (0.69-1.18) | 0.441 | 1.09 (0.92-1.29) | 0.328 |
| Gastrointestinal bleeding | 0.97 (0.92-1.03) | 0.305 | 0.74 (0.70-0.77) | <0.001 | 0.91 (0.82-1.01) | 0.068 | 0.77 (0.72-0.83) | <0.001 | 0.99 (0.86-1.15) | 0.920 | 0.68 (0.62-0.76) | <0.001 |
| Upper gastrointestinal bleeding | 0.93 (0.86-1.01) | 0.084 | 0.81 (0.75-0.86) | <0.001 | 0.86 (0.75-0.99) | 0.046 | 0.85 (0.78-0.94) | <0.001 | 1.05 (0.86-1.29) | 0.602 | 0.75 (0.66-0.87) | <0.001 |
| Lower gastrointestinal bleeding | 0.94 (0.88-1.00) | 0.066 | 0.67 (0.63-0.71) | <0.001 | 0.89 (0.77-1.02) | 0.084 | 0.76 (0.69-0.83) | <0.001 | 0.95 (0.79-1.15) | 0.588 | 0.62 (0.54-0.71) | <0.001 |
| Urogenital bleeding | 0.89 (0.83-0.96) | <0.001 | 0.87 (0.82-0.93) | <0.001 | 0.84 (0.74-0.96) | 0.012 | 0.94 (0.87-1.03) | 0.179 | 0.91 (0.75-1.10) | 0.317 | 0.87 (0.76-0.98) | 0.026 |
| Bleeding from other sites | 0.88 (0.85-0.93) | <0.001 | 0.94 (0.91-0.98) | 0.002 | 0.96 (0.89-1.03) | 0.252 | 1.06 (1.01-1.12) | 0.029 | 0.90 (0.81-0.99) | 0.046 | 0.90 (0.84-0.97) | 0.003 |

**eTable 11:** Adjusted hazard ratios of outcomes compared between **A)** (individual) NOACs and VKAs, and **B)** compared between individual NOAC types after IPTW using an intention-to-treat approach.

CI: confidence interval; CRNMB: clinically relevant non-major bleeding; HR: hazard ratio; IPTW: inverse probability of treatment weighting; MB: major bleeding; NOAC: non-vitamin K antagonist oral anticoagulant; VKA: vitamin K antagonist; vs: versus.

eTable 12: Adjusted HRs of outcomes after IPTW (patients with ICD-coded hospital discharge diagnosis of AF)

**A)**

|  | **NOAC vs VKA** | | **Dabigatran vs VKA** | | **Rivaroxaban vs VKA** | | **Apixaban vs VKA** | | **Edoxaban vs VKA** | |
| --- | --- | --- | --- | --- | --- | --- | --- | --- | --- | --- |
|  | **HR (95%CI)** | **p-value** | **HR (95%CI)** | **p-value** | **HR (95%CI)** | **p-value** | **HR (95%CI)** | **p-value** | **HR (95%CI)** | **p-value** |
| **Effectiveness** |  |  |  |  |  |  |  |  |  |  |
| Stroke or systemic embolism | 0.72 (0.66-0.78) | <0.001 | 0.81 (0.72-0.92) | <0.001 | 0.79 (0.72-0.87) | <0.001 | 0.69 (0.61-0.77) | <0.001 | 0.53 (0.39-0.73) | <0.001 |
| Stroke | 0.72 (0.65-0.79) | <0.001 | 0.80 (0.70-0.92) | <0.001 | 0.79 (0.71-0.88) | <0.001 | 0.71 (0.63-0.80) | <0.001 | 0.51 (0.36-0.72) | <0.001 |
| Ischemic stroke | 0.61 (0.55-0.69) | <0.001 | 0.76 (0.65-0.89) | <0.001 | 0.69 (0.61-0.78) | <0.001 | 0.60 (0.52-0.70) | <0.001 | 0.49 (0.32-0.75) | <0.001 |
| Systemic embolism | 0.70 (0.57-0.86) | <0.001 | 0.86 (0.66-1.13) | 0.291 | 0.77 (0.62-0.96) | 0.019 | 0.56 (0.43-0.72) | <0.001 | 0.63 (0.34-1.16) | 0.139 |
| All-cause mortality | 0.80 (0.76-0.84) | <0.001 | 0.79 (0.73-0.85) | <0.001 | 0.87 (0.82-0.91) | <0.001 | 0.83 (0.78-0.88) | <0.001 | 0.58 (0.50-0.68) | <0.001 |
| Myocardial infarction | 0.95 (0.82-1.09) | 0.469 | 1.14 (0.93-1.39) | 0.198 | 0.98 (0.84-1.14) | 0.803 | 0.99 (0.83-1.18) | 0.889 | 0.77 (0.53-1.14) | 0.191 |
| **Safety** |  |  |  |  |  |  |  |  |  |  |
| MB/CRNMB | 0.94 (0.90-0.99) | 0.020 | 0.92 (0.86-0.98) | 0.017 | 1.03 (0.98-1.08) | 0.268 | 0.84 (0.79-0.90) | <0.001 | 0.83 (0.72-0.96) | 0.010 |
| Major bleeding | 0.85 (0.80-0.91) | <0.001 | 0.91 (0.83-0.99) | 0.035 | 1.00 (0.94-1.07) | 0.968 | 0.74 (0.68-0.80) | <0.001 | 0.87 (0.71-1.06) | 0.164 |
| CRNMB | 1.07 (0.99-1.14) | 0.072 | 0.95 (0.86-1.05) | 0.303 | 1.07 (1.00-1.16) | 0.054 | 0.96 (0.89-1.05) | 0.375 | 0.81 (0.67-0.96) | 0.018 |
| Intracranial hemorrhage | 0.67 (0.60-0.75) | <0.001 | 0.76 (0.65-0.89) | <0.001 | 0.84 (0.75-0.95) | 0.007 | 0.65 (0.56-0.76) | <0.001 | 0.64 (0.39-1.04) | 0.074 |
| Gastrointestinal bleeding | 1.08 (0.98-1.18) | 0.131 | 1.14 (1.00-1.30) | 0.052 | 1.26 (1.14-1.39) | <0.001 | 0.86 (0.77-0.97) | 0.014 | 1.26 (0.95-1.68) | 0.110 |
| Upper gastrointestinal bleeding | 1.09 (0.95-1.25) | 0.219 | 1.05 (0.87-1.28) | 0.594 | 1.16 (1.01-1.34) | 0.038 | 0.93 (0.79-1.09) | 0.359 | 1.61 (1.07-2.43) | 0.022 |
| Lower gastrointestinal bleeding | 1.02 (0.91-1.15) | 0.690 | 1.11 (0.95-1.31) | 0.178 | 1.31 (1.16-1.48) | <0.001 | 0.77 (0.66-0.90) | <0.001 | 1.13 (0.78-1.64) | 0.521 |
| Urogenital bleeding | 1.10 (0.97-1.24) | 0.155 | 1.05 (0.88-1.24) | 0.593 | 1.19 (1.05-1.36) | 0.008 | 0.90 (0.78-1.05) | 0.197 | 0.79 (0.55-1.15) | 0.225 |
| Bleeding from other sites | 0.93 (0.87-0.99) | 0.041 | 0.83 (0.76-0.92) | <0.001 | 0.96 (0.89-1.02) | 0.204 | 0.84 (0.78-0.91) | <0.001 | 0.76 (0.64-0.89) | <0.001 |

**B)**

|  | **Dabigatran vs Rivaroxaban** | | **Apixaban vs Rivaroxaban** | | **Edoxaban vs Rivaroxaban** | | **Apixaban vs Dabigatran** | | **Dabigatran vs Edoxaban** | | **Apixaban vs Edoxaban** | |
| --- | --- | --- | --- | --- | --- | --- | --- | --- | --- | --- | --- | --- |
|  | **HR (95%CI)** | **p-value** | **HR (95%CI)** | **p-value** | **HR (95%CI)** | **p-value** | **HR (95%CI)** | **p-value** | **HR (95%CI)** | **p-value** | **HR (95%CI)** | **p-value** |
| **Effectiveness** |  |  |  |  |  |  |  |  |  |  |  |  |
| Stroke or systemic embolism | 0.94 (0.86-1.04) | 0.250 | 0.90 (0.83-0.98) | 0.011 | 0.87 (0.73-1.04) | 0.135 | 0.99 (0.88-1.11) | 0.848 | 1.17 (0.93-1.47) | 0.185 | 0.97 (0.83-1.12) | 0.659 |
| Stroke | 0.91 (0.82-1.01) | 0.090 | 0.91 (0.84-0.99) | 0.045 | 0.86 (0.70-1.05) | 0.139 | 1.03 (0.91-1.16) | 0.693 | 1.21 (0.94-1.56) | 0.130 | 1.00 (0.85-1.19) | 0.993 |
| Ischemic stroke | 1.02 (0.90-1.15) | 0.801 | 0.91 (0.81-1.01) | 0.089 | 0.90 (0.70-1.17) | 0.428 | 0.93 (0.80-1.08) | 0.349 | 1.33 (0.98-1.80) | 0.063 | 1.01 (0.82-1.25) | 0.919 |
| Systemic embolism | 1.15 (0.92-1.45) | 0.223 | 0.80 (0.65-0.98) | 0.033 | 0.94 (0.65-1.38) | 0.768 | 0.75 (0.57-0.98) | 0.034 | 1.04 (0.63-1.72) | 0.872 | 0.81 (0.59-1.13) | 0.217 |
| All-cause mortality | 0.87 (0.82-0.92) | <0.001 | 0.97 (0.93-1.01) | 0.156 | 0.88 (0.80-0.97) | 0.008 | 1.17 (1.10-1.25) | <0.001 | 0.93 (0.81-1.06) | 0.277 | 1.12 (1.04-1.21) | 0.003 |
| Myocardial infarction | 1.10 (0.94-1.28) | 0.225 | 0.93 (0.82-1.05) | 0.239 | 0.78 (0.62-0.98) | 0.034 | 0.91 (0.77-1.08) | 0.297 | 1.38 (1.02-1.86) | 0.038 | 0.99 (0.80-1.21) | 0.891 |
| **Safety** |  |  |  |  |  |  |  |  |  |  |  |  |
| MB/CRNMB | 0.87 (0.82-0.92) | <0.001 | 0.83 (0.80-0.87) | <0.001 | 0.90 (0.84-0.98) | 0.013 | 0.94 (0.88-0.99) | 0.040 | 1.00 (0.90-1.12) | 0.965 | 0.83 (0.78-0.89) | <0.001 |
| Major bleeding | 0.88 (0.82-0.95) | <0.001 | 0.74 (0.70-0.78) | <0.001 | 0.89 (0.80-0.99) | 0.042 | 0.82 (0.76-0.89) | <0.001 | 1.06 (0.91-1.23) | 0.478 | 0.78 (0.70-0.85) | <0.001 |
| CRNMB | 0.86 (0.80-0.93) | <0.001 | 0.92 (0.87-0.97) | 0.004 | 0.91 (0.82-1.01) | 0.076 | 1.05 (0.97-1.15) | 0.215 | 0.94 (0.81-1.08) | 0.393 | 0.87 (0.80-0.96) | 0.003 |
| Intracranial hemorrhage | 0.93 (0.81-1.05) | 0.244 | 0.80 (0.71-0.89) | <0.001 | 0.93 (0.70-1.23) | 0.601 | 0.91 (0.77-1.07) | 0.244 | 1.12 (0.78-1.61) | 0.527 | 1.13 (0.90-1.42) | 0.306 |
| Gastrointestinal bleeding | 0.88 (0.80-0.96) | 0.007 | 0.67 (0.62-0.72) | <0.001 | 0.85 (0.74-0.98) | 0.026 | 0.74 (0.66-0.83) | <0.001 | 1.03 (0.84-1.27) | 0.755 | 0.65 (0.57-0.75) | <0.001 |
| Upper gastrointestinal bleeding | 0.86 (0.74-0.99) | 0.040 | 0.77 (0.69-0.86) | <0.001 | 0.82 (0.67-0.99) | 0.049 | 0.80 (0.68-0.94) | 0.006 | 1.13 (0.85-1.51) | 0.393 | 0.75 (0.63-0.90) | 0.002 |
| Lower gastrointestinal bleeding | 0.84 (0.75-0.94) | 0.004 | 0.56 (0.51-0.62) | <0.001 | 0.78 (0.65-0.93) | 0.007 | 0.70 (0.61-0.81) | <0.001 | 0.97 (0.74-1.27) | 0.816 | 0.58 (0.48-0.69) | <0.001 |
| Urogenital bleeding | 0.88 (0.77-0.99) | 0.039 | 0.80 (0.72-0.88) | <0.001 | 0.81 (0.68-0.96) | 0.018 | 0.88 (0.76-1.01) | 0.073 | 0.94 (0.72-1.22) | 0.641 | 0.76 (0.64-0.89) | <0.001 |
| Bleeding from other sites | 0.84 (0.78-0.91) | <0.001 | 0.90 (0.85-0.95) | <0.001 | 0.92 (0.83-1.02) | 0.111 | 1.03 (0.95-1.12) | 0.440 | 0.97 (0.84-1.12) | 0.723 | 0.87 (0.79-0.95) | 0.002 |

**eTable 12:** Adjusted hazard ratios of outcomes compared between **A)** (individual) NOACs and VKAs, and **B)** compared between individual NOAC types after IPTW in the subgroup of OAC-naïve subjects with an ICD-coded diagnosis of AF ≤1 year before or ≤90 days after the index date

AF: Atrial fibrillation; CI: confidence interval; CRNMB: clinically relevant non-major bleeding; HR: hazard ratio; ICD: International Classification of Diseases; IPTW: inverse probability of treatment weighting; MB: major bleeding; NOAC: non-vitamin K antagonist oral anticoagulant; VKA: vitamin K antagonist; vs: versus.

eTable 13: Adjusted HRs of outcomes after IPTW (October 1^st^, 2016 – January 1^st^, 2019)

**A)**

|  | **NOAC vs VKA** | | **Dabigatran vs VKA** | | **Rivaroxaban vs VKA** | | **Apixaban vs VKA** | | **Edoxaban vs VKA** | |
| --- | --- | --- | --- | --- | --- | --- | --- | --- | --- | --- |
|  | **HR (95%CI)** | **p-value** | **HR (95%CI)** | **p-value** | **HR (95%CI)** | **p-value** | **HR (95%CI)** | **p-value** | **HR (95%CI)** | **p-value** |
| **Effectiveness** |  |  |  |  |  |  |  |  |  |  |
| Stroke or systemic embolism | 0.60 (0.50-0.72) | <0.001 | 0.58 (0.45-0.73) | <0.001 | 0.64 (0.53-0.78) | <0.001 | 0.60 (0.50-0.72) | <0.001 | 0.61 (0.50-0.74) | <0.001 |
| Stroke | 0.67 (0.54-0.83) | <0.001 | 0.71 (0.54-0.94) | 0.016 | 0.72 (0.57-0.91) | 0.005 | 0.69 (0.55-0.87) | 0.002 | 0.69 (0.54-0.87) | 0.002 |
| Ischemic stroke | 0.63 (0.48-0.83) | <0.001 | 0.75 (0.54-1.05) | 0.096 | 0.69 (0.51-0.92) | 0.013 | 0.67 (0.50-0.89) | 0.005 | 0.65 (0.48-0.87) | 0.004 |
| Systemic embolism | 0.46 (0.34-0.62) | <0.001 | 0.38 (0.24-0.60) | <0.001 | 0.51 (0.36-0.73) | <0.001 | 0.39 (0.28-0.54) | <0.001 | 0.48 (0.33-0.68) | <0.001 |
| All-cause mortality | 0.60 (0.54-0.66) | <0.001 | 0.66 (0.57-0.77) | <0.001 | 0.69 (0.62-0.77) | <0.001 | 0.68 (0.62-0.76) | <0.001 | 0.60 (0.54-0.68) | <0.001 |
| Myocardial infarction | 0.84 (0.64-1.12) | 0.239 | 1.05 (0.73-1.52) | 0.784 | 1.07 (0.79-1.45) | 0.652 | 0.82 (0.61-1.09) | 0.172 | 0.86 (0.64-1.17) | 0.348 |
| **Safety** |  |  |  |  |  |  |  |  |  |  |
| MB/CRNMB | 0.78 (0.71-0.86) | <0.001 | 0.86 (0.76-0.98) | 0.019 | 0.90 (0.82-0.99) | 0.044 | 0.73 (0.66-0.80) | <0.001 | 0.86 (0.78-0.95) | 0.004 |
| Major bleeding | 0.80 (0.70-0.92) | 0.002 | 0.97 (0.81-1.15) | 0.715 | 0.95 (0.82-1.09) | 0.454 | 0.71 (0.62-0.82) | <0.001 | 0.93 (0.80-1.07) | 0.304 |
| CRNMB | 0.77 (0.68-0.86) | <0.001 | 0.79 (0.67-0.93) | 0.004 | 0.89 (0.79-1.01) | 0.064 | 0.74 (0.66-0.84) | <0.001 | 0.82 (0.72-0.93) | 0.002 |
| Intracranial hemorrhage | 0.85 (0.61-1.20) | 0.369 | 0.88 (0.58-1.34) | 0.558 | 0.90 (0.62-1.29) | 0.560 | 0.91 (0.64-1.29) | 0.589 | 0.93 (0.65-1.33) | 0.699 |
| Gastrointestinal bleeding | 0.95 (0.78-1.17) | 0.660 | 1.23 (0.96-1.58) | 0.106 | 1.27 (1.03-1.55) | 0.022 | 0.75 (0.61-0.93) | 0.010 | 1.15 (0.93-1.42) | 0.212 |
| Upper gastrointestinal bleeding | 0.98 (0.73-1.31) | 0.890 | 1.31 (0.92-1.86) | 0.129 | 1.34 (1.01-1.77) | 0.043 | 0.82 (0.61-1.11) | 0.208 | 1.15 (0.86-1.54) | 0.350 |
| Lower gastrointestinal bleeding | 0.96 (0.73-1.25) | 0.754 | 1.15 (0.84-1.59) | 0.385 | 1.33 (1.02-1.74) | 0.034 | 0.69 (0.52-0.92) | 0.011 | 1.13 (0.86-1.50) | 0.377 |
| Urogenital bleeding | 0.78 (0.62-0.99) | 0.044 | 0.87 (0.63-1.20) | 0.383 | 1.07 (0.84-1.36) | 0.581 | 0.70 (0.54-0.89) | 0.005 | 0.84 (0.65-1.09) | 0.183 |
| Bleeding from other sites | 0.70 (0.62-0.78) | <0.001 | 0.72 (0.61-0.84) | <0.001 | 0.79 (0.70-0.89) | <0.001 | 0.67 (0.60-0.75) | <0.001 | 0.77 (0.68-0.86) | <0.001 |

**B)**

|  | **Dabigatran vs Rivaroxaban** | | **Apixaban vs Rivaroxaban** | | **Edoxaban vs Rivaroxaban** | | **Apixaban vs Dabigatran** | | **Dabigatran vs Edoxaban** | | **Apixaban vs Edoxaban** | |
| --- | --- | --- | --- | --- | --- | --- | --- | --- | --- | --- | --- | --- |
|  | **HR (95%CI)** | **p-value** | **HR (95%CI)** | **p-value** | **HR (95%CI)** | **p-value** | **HR (95%CI)** | **p-value** | **HR (95%CI)** | **p-value** | **HR (95%CI)** | **p-value** |
| **Effectiveness** |  |  |  |  |  |  |  |  |  |  |  |  |
| Stroke or systemic embolism | 0.97 (0.80-1.18) | 0.781 | 0.91 (0.79-1.04) | 0.179 | 0.94 (0.81-1.09) | 0.402 | 0.97 (0.81-1.16) | 0.743 | 1.02 (0.84-1.24) | 0.828 | 0.97 (0.85-1.10) | 0.649 |
| Stroke | 1.01 (0.81-1.26) | 0.911 | 0.95 (0.82-1.10) | 0.498 | 0.95 (0.80-1.12) | 0.509 | 0.95 (0.78-1.16) | 0.638 | 1.06 (0.86-1.32) | 0.565 | 1.00 (0.87-1.16) | 0.952 |
| Ischemic stroke | 1.22 (0.94-1.60) | 0.138 | 1.02 (0.83-1.24) | 0.883 | 0.97 (0.78-1.20) | 0.786 | 0.84 (0.66-1.07) | 0.163 | 1.24 (0.96-1.61) | 0.099 | 1.05 (0.88-1.27) | 0.577 |
| Systemic embolism | 0.94 (0.62-1.43) | 0.760 | 0.75 (0.56-1.00) | 0.051 | 0.93 (0.69-1.26) | 0.650 | 0.86 (0.58-1.28) | 0.451 | 0.98 (0.65-1.48) | 0.919 | 0.80 (0.61-1.06) | 0.128 |
| All-cause mortality | 0.85 (0.76-0.95) | 0.005 | 1.03 (0.96-1.10) | 0.402 | 0.89 (0.82-0.96) | 0.003 | 1.30 (1.17-1.44) | <0.001 | 0.93 (0.83-1.04) | 0.206 | 1.17 (1.09-1.25) | <0.001 |
| Myocardial infarction | 1.04 (0.80-1.36) | 0.752 | 0.86 (0.72-1.03) | 0.106 | 0.89 (0.73-1.09) | 0.255 | 0.88 (0.69-1.14) | 0.332 | 1.15 (0.88-1.50) | 0.293 | 0.99 (0.83-1.18) | 0.895 |
| **Safety** |  |  |  |  |  |  |  |  |  |  |  |  |
| MB/CRNMB | 0.88 (0.80-0.97) | 0.011 | 0.81 (0.76-0.87) | <0.001 | 0.95 (0.89-1.01) | 0.123 | 0.93 (0.85-1.02) | 0.148 | 0.91 (0.83-0.99) | 0.046 | 0.86 (0.81-0.91) | <0.001 |
| Major bleeding | 0.94 (0.83-1.08) | 0.395 | 0.76 (0.69-0.83) | <0.001 | 0.95 (0.87-1.04) | 0.295 | 0.81 (0.71-0.92) | <0.001 | 0.97 (0.85-1.11) | 0.678 | 0.79 (0.72-0.86) | <0.001 |
| CRNMB | 0.82 (0.73-0.93) | 0.002 | 0.84 (0.78-0.91) | <0.001 | 0.93 (0.86-1.02) | 0.113 | 1.06 (0.94-1.20) | 0.314 | 0.85 (0.75-0.96) | 0.007 | 0.91 (0.85-0.99) | 0.021 |
| Intracranial hemorrhage | 1.01 (0.74-1.39) | 0.936 | 1.11 (0.90-1.37) | 0.331 | 1.10 (0.88-1.38) | 0.389 | 1.11 (0.83-1.49) | 0.491 | 0.91 (0.67-1.24) | 0.546 | 1.06 (0.88-1.28) | 0.540 |
| Gastrointestinal bleeding | 0.88 (0.74-1.05) | 0.157 | 0.61 (0.54-0.69) | <0.001 | 0.88 (0.78-0.99) | 0.037 | 0.68 (0.58-0.81) | <0.001 | 0.99 (0.83-1.17) | 0.907 | 0.68 (0.61-0.77) | <0.001 |
| Upper gastrointestinal bleeding | 0.86 (0.68-1.09) | 0.211 | 0.65 (0.55-0.76) | <0.001 | 0.81 (0.69-0.96) | 0.014 | 0.72 (0.57-0.91) | 0.005 | 1.08 (0.85-1.37) | 0.527 | 0.78 (0.67-0.91) | 0.002 |
| Lower gastrointestinal bleeding | 0.85 (0.68-1.06) | 0.150 | 0.53 (0.45-0.62) | <0.001 | 0.86 (0.74-1.00) | 0.051 | 0.63 (0.51-0.79) | <0.001 | 0.96 (0.77-1.20) | 0.709 | 0.61 (0.52-0.71) | <0.001 |
| Urogenital bleeding | 0.69 (0.55-0.86) | <0.001 | 0.66 (0.57-0.76) | <0.001 | 0.82 (0.71-0.95) | 0.007 | 0.97 (0.77-1.21) | 0.759 | 0.83 (0.66-1.04) | 0.107 | 0.81 (0.71-0.93) | 0.003 |
| Bleeding from other sites | 0.87 (0.77-0.99) | 0.028 | 0.85 (0.79-0.92) | <0.001 | 0.95 (0.87-1.04) | 0.252 | 1.01 (0.89-1.14) | 0.907 | 0.88 (0.78-0.99) | 0.045 | 0.90 (0.83-0.97) | 0.006 |

**eTable 13:** Adjusted hazard ratios of outcomes compared between **A)** (individual) NOACs and VKAs, and **B)** compared between individual NOAC types after IPTW in subgroup of subjects having initiated treatment between October 1^st^, 2016 and January 1^st^, 2019.

CI: confidence interval; CRNMB: clinically relevant non-major bleeding; HR: hazard ratio; IPTW: inverse probability of treatment weighting; MB: major bleeding; NOAC: non-vitamin K antagonist oral anticoagulant; VKA: vitamin K antagonist; vs: versus.

eTable 14: The risk of AF-related mortality

**A)**

|  | **VKA** | **NOAC** | **Dabigatran** | **Rivaroxaban** | **Apixaban** | **Edoxaban** |
| --- | --- | --- | --- | --- | --- | --- |
| **Outcome** | **Events (per 100 PY)** | **Events (per 100 PY)** | **Events (per 100 PY)** | **Events (per 100 PY)** | **Events (per 100 PY)** | **Events (per 100 PY)** |
| **Effectiveness** |  |  |  |  |  |  |
| All-cause mortality | 4264 (8.19) | 20589 (7.44) | 2687 (5.89) | 8688 (7.12) | 7850 (8.52) | 1364 (8.06) |
| AF-related mortality | 751 (1.44) | 3569 (1.29) | 481 (1.05) | 1496 (1.23) | 1298 (1.41) | 294 (1.74) |

**B)**

|  | **NOAC vs VKA** | | **Dabigatran vs VKA** | | **Rivaroxaban vs VKA** | | **Apixaban vs VKA** | | **Edoxaban vs VKA** | |
| --- | --- | --- | --- | --- | --- | --- | --- | --- | --- | --- |
|  | **HR (95%CI)** | **p-value** | **HR (95%CI)** | **p-value** | **HR (95%CI)** | **p-value** | **HR (95%CI)** | **p-value** | **HR (95%CI)** | **p-value** |
| **Effectiveness** |  |  |  |  |  |  |  |  |  |  |
| All-cause mortality | 0.76 (0.74-0.79) | <0.001 | 0.78 (0.74-0.83) | <0.001 | 0.83 (0.79-0.86) | <0.001 | 0.82 (0.78-0.85) | <0.001 | 0.60 (0.54-0.68) | <0.001 |
| AF-related mortality | 0.76 (0.70-0.83) | <0.001 | 0.78 (0.68-0.89) | <0.001 | 0.81 (0.74-0.89) | <0.001 | 0.79 (0.71-0.89) | <0.001 | 0.58 (0.45-0.75) | <0.001 |

**C)**

|  | **Dabigatran vs Rivaroxaban** | | **Apixaban vs Rivaroxaban** | | **Edoxaban vs Rivaroxaban** | | **Apixaban vs Dabigatran** | | **Dabigatran vs Edoxaban** | | **Apixaban vs Edoxaban** | |
| --- | --- | --- | --- | --- | --- | --- | --- | --- | --- | --- | --- | --- |
|  | **HR (95%CI)** | **p-value** | **HR (95%CI)** | **p-value** | **HR (95%CI)** | **p-value** | **HR (95%CI)** | **p-value** | **HR (95%CI)** | **p-value** | **HR (95%CI)** | **p-value** |
| **Effectiveness** |  |  |  |  |  |  |  |  |  |  |  |  |
| All-cause mortality | 0.88 (0.84-0.92) | <0.001 | 1.00 (0.96-1.03) | 0.818 | 0.89 (0.82-0.96) | 0.003 | 1.17 (1.12-1.23) | <0.001 | 0.93 (0.83-1.04) | 0.206 | 1.17 (1.09-1.25) | <0.001 |
| AF-related mortality | 0.91 (0.82-1.01) | 0.075 | 0.99 (0.91-1.07) | 0.780 | 1.04 (0.87-1.24) | 0.663 | 1.09 (0.97-1.23) | 0.157 | 0.87 (0.68-1.12) | 0.283 | 0.98 (0.84-1.13) | 0.747 |

**eTable 14:** The **A)** number of events and crude event rates per 100 person-years, and adjusted hazard ratios of the risks of all-cause mortality and AF-related mortality compared between **B)** (individual) NOACs and VKAs, and **C)** compared between individual NOAC types after IPTW. AF-related mortality was defined as a death occurring within 60 days after an event of thromboembolism, bleeding or myocardial infarction.

CI: confidence interval; IPTW: inverse probability of treatment weighting; NOAC: non-vitamin K antagonist oral anticoagulant; PY: person-year; VKA: vitamin K antagonist; vs: versus.

eTable 15: Significant interactions between treatment and covariates regarding the risk of mortality between individual NOACs using doubly robust estimation models

|  | **Significant interaction terms^‡^** | **aHR (95%CI)^*^** | **p-value** |
| --- | --- | --- | --- |
| **Apixaban vs Dabigatran** | | | |
|  | Apixaban & diabetes mellitus | 1.19 (1.05-1.34) | 0.005 |
| **Apixaban vs Edoxaban** | | | |
|  | Apixaban & digoxin | 1.34 (1.10-1.62) | 0.003 |
|  | Apixaban & corticosteroids | 0.85 (0.74-0.99) | 0.034 |
| **Apixaban vs Rivaroxaban** | | | |
|  | Apixaban & lower GI tract disorders | 1.19 (1.03-1.36) | 0.017 |
| **Dabigatran vs Rivaroxaban** | | | |
|  | Dabigatran & lower GI tract disorders | 1.28 (1.06-1.55) | 0.012 |
| **Edoxaban vs Rivaroxaban** | | | |
|  | Edoxaban & lower GI tract disorders | 1.39 (1.02-1.91) | 0.039 |
|  | Edoxaban & chronic liver disease | 0.69 (0.50-0.95) | 0.024 |
| **Dabigatran vs Edoxaban** | | | |
|  | N.S. | N.S. | N.S. |

**eTable 15:** Significant interactions between treatment and covariates regarding the risk of mortality between individual NOACs using doubly robust estimation models.

The treatment model was fitted with logistic regression models including the 37 covariates described in Table 1 (baseline demographics, comorbidities, medication history and clinical risk scores) stratified by calendar year, to calculate propensity scores and stabilized weights for stabilized IPTW. The response/outcome model was fitted with multivariable adjusted, weighted Cox proportional hazard regression models including 35 covariates (baseline demographics, comorbidities, medication history and NOAC dose). Clinical risk scores were not included to avoid multicollinearity issues. Only statistically significant factors using a two-sided p-value of <0.05 with backward elimination were retained in the final response/outcome models. Interaction terms between the treatment (e.g. apixaban vs dabigatran) and every statistically significant factor were added to the multivariable adjusted, weighted Cox proportional hazard regression models. Only statistically significant interaction terms were retained in the final response/outcome models and summarized in the table. HRs with 95% CIs and p-values for the risk of mortality are presented of statistically significant interaction terms.

^*^ After weighting, Cox proportional hazard regression models were adjusted for:

- *Apixaban vs Dabigatran*: Age, sex, hypertension, coronary artery disease, congestive heart failure, valvular heart disease, peripheral artery disease, dyslipidemia, chronic liver disease, chronic lung disease, cancer, lower GI tract disorders, diabetes mellitus, anemia, dementia, frailty, prior thromboembolism, number of concomitant drugs, interaction term with treatment and concomitant drugs, digoxin, class I AAD, acetylsalicylic acid, NSAID, oral corticosteroids, SSRI/SNRI, NOAC dose, and interaction term with treatment and NOAC dose.
- *Apixaban vs Edoxaban*: Age, sex, hypertension, coronary artery disease, congestive heart failure, peripheral artery disease, dyslipidemia, chronic liver disease, chronic lung disease, cancer, lower GI tract disorders, diabetes mellitus, anemia, dementia, history of falling, frailty, prior thromboembolism, prior major bleeding, number of concomitant drugs, digoxin, class I AAD, acetylsalicylic acid, NSAID, oral corticosteroids, SSRI/SNRI and NOAC dose.
- *Apixaban vs Rivaroxaban*: Age, sex, hypertension, coronary artery disease, congestive heart failure, valvular heart disease, peripheral artery disease, dyslipidemia, chronic liver disease, chronic lung disease, cancer, lower GI tract disorders, diabetes mellitus, anemia, dementia, history of falling, frailty, prior thromboembolism, number of concomitant drugs, interaction term with treatment and concomitant drugs, digoxin, class I AAD, acetylsalicylic acid, NSAID, oral corticosteroids, SSRI/SNRI, NOAC dose and interaction term with treatment and NOAC dose.
- *Dabigatran vs Rivaroxaban*: Age, sex, hypertension, coronary artery disease, congestive heart failure, valvular heart disease, peripheral artery disease, dyslipidemia, chronic liver disease, chronic lung disease, cancer, lower GI tract disorders, diabetes mellitus, anemia, dementia, history of falling, frailty, prior thromboembolism, number of concomitant drugs, beta blockers, digoxin, class I AAD, class III AAD, acetylsalicylic acid, NSAID, oral corticosteroids, SSRI/SNRI and NOAC dose.
- *Edoxaban vs Rivaroxaban*: Age, sex, hypertension, congestive heart failure, peripheral artery disease, dyslipidemia, chronic liver disease, chronic lung disease, cancer, lower GI tract disorders, diabetes mellitus, anemia, dementia, history of falling, frailty, prior thromboembolism, number of concomitant drugs, class I AAD, acetylsalicylic acid, NSAID, oral corticosteroids and NOAC dose.
- *Dabigatran vs Edoxaban*: Age, sex, hypertension, congestive heart failure, peripheral artery disease, dyslipidemia, chronic liver disease, chronic lung disease, cancer, diabetes mellitus, anemia, dementia, frailty, prior thromboembolism, number of concomitant drugs, class I AAD, acetylsalicylic acid, P2Y12 inhibitors, NSAID, oral corticosteroids, and NOAC dose.

AAD: antiarrhythmic drug; CI: confidence interval; DRE: doubly robust estimation; GI: gastrointestinal; (a)HR: (adjusted) hazard ratio; IPTW: inverse probability of treatment weighting; NOAC: non-vitamin K antagonist oral anticoagulant; N.S.: not significant; NSAID: non-steroidal anti-inflammatory drug; SNRI: serotonin and norepinephrine reuptake inhibitor; SSRI: selective serotonin reuptake inhibitor; vs: versus.

# Supplemental figures

## eFigure 1: Overview of study design

**
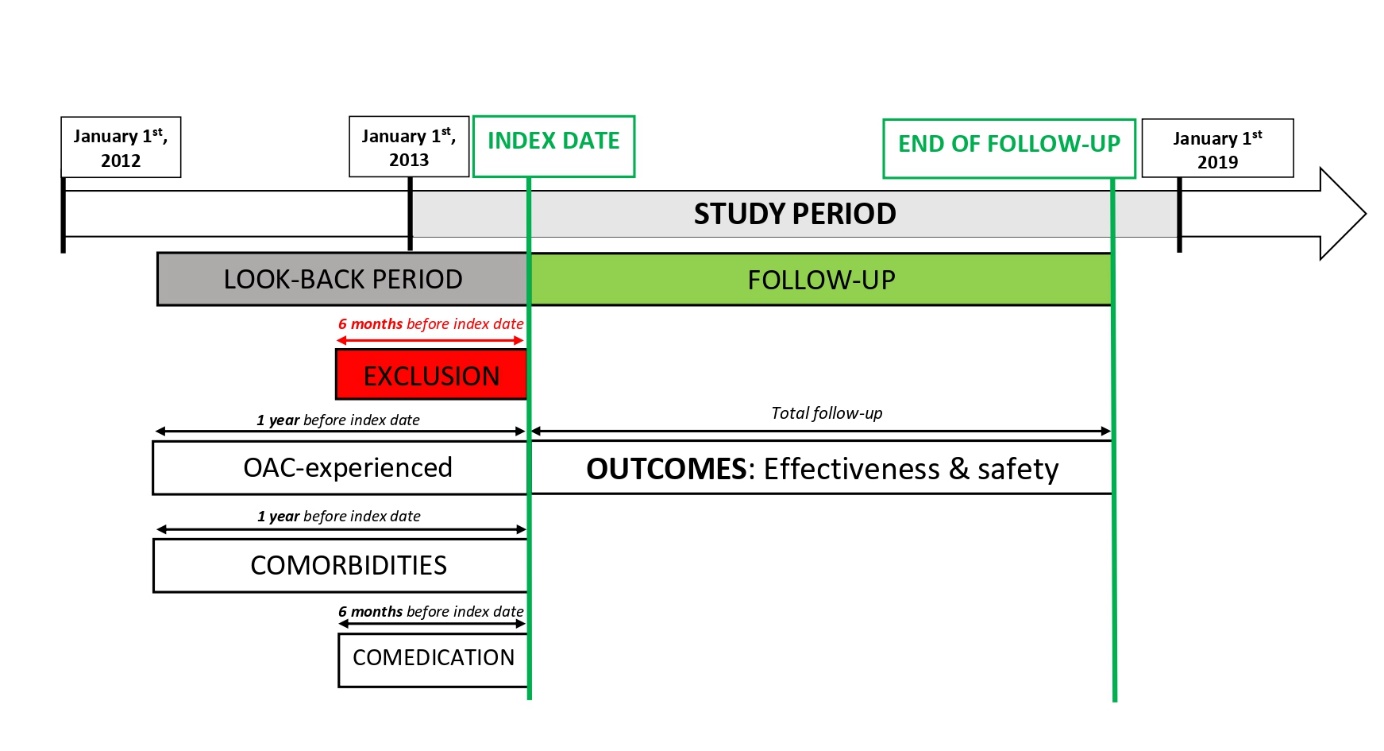
**

**eFigure 1:** Overview of study design.

**Study period:** January 1^st^, 2013 – January 1^st^, 2019. **Look-back period:** Up to 1 year before index date (maximum up until January 1^st^, 2012). **Index date:** First dispensing of oral anticoagulant (NOAC or VKA) during study period to subjects ≥45 years. **End of follow-up:** Follow-up ended in case of discontinuation, switching, death, emigration or end of the study period, whichever occurred first. **Exclusion:** Total hip or knee replacement surgery, or diagnosis of deep vein thrombosis or pulmonary embolism ≤6 months before the index date. Moreover, subjects with valvular AF (mechanical prosthetic heart valve or moderate/severe mitral stenosis), end-stage renal disease (chronic kidney disease stage V and/or dialysis), subjects with ≥2 prescription claims of different oral anticoagulant types or dosages on the index date, or subjects treated with NOAC dosages not approved for stroke prevention in AF (e.g. rivaroxaban 10 mg) were excluded. **OAC-experienced:** Subjects were considered as OAC-experienced if an OAC prescription was filled ≤1 year before the index date. Only OAC-naïve subjects were included. **Comorbidities:** Comorbidities were identified using specific ICD-coded diagnoses (e.g. cancer) from the MHD, medical procedure codes (e.g. cancer-related surgery) from the IMA database and/or ATC-coded prescription claims (e.g. antineoplastic drugs) from the IMA database ≤1 year before the index date. **Comedication use:** Comedication dispensed up to 6 months before the index date. **Outcome:** Effectiveness and safety of oral anticoagulants.

AF: Atrial fibrillation; ATC: Anatomical Therapeutic Chemical Classification; IMA: InterMutualistic Agency; MHD: Minimal Hospital Dataset; NOAC: non-vitamin K antagonist oral anticoagulant; OAC: oral anticoagulant; vitamin K antagonist.

## eFigure 2: Love plots (main analysis)

**A)**

**
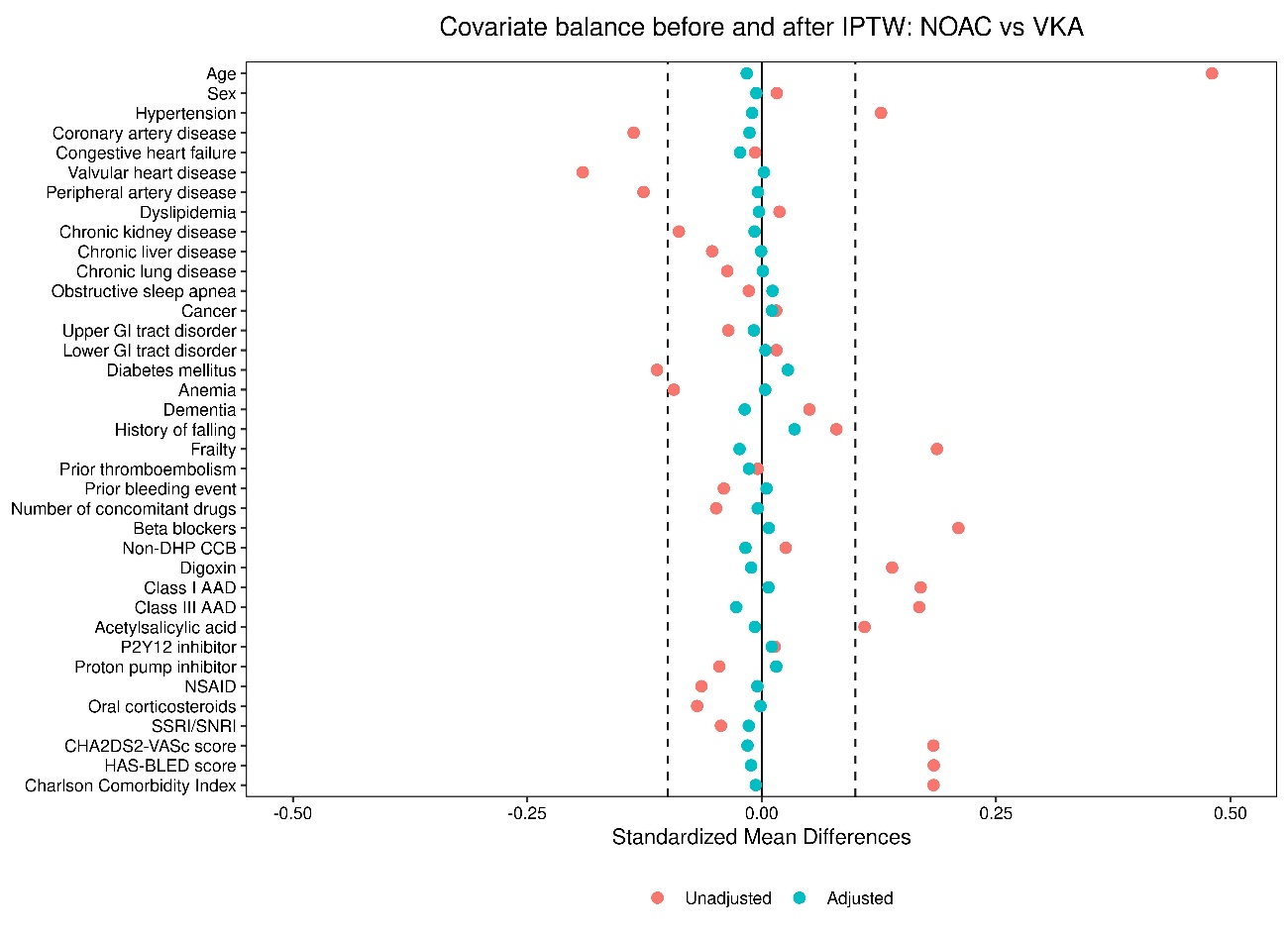
**

**B)**

**
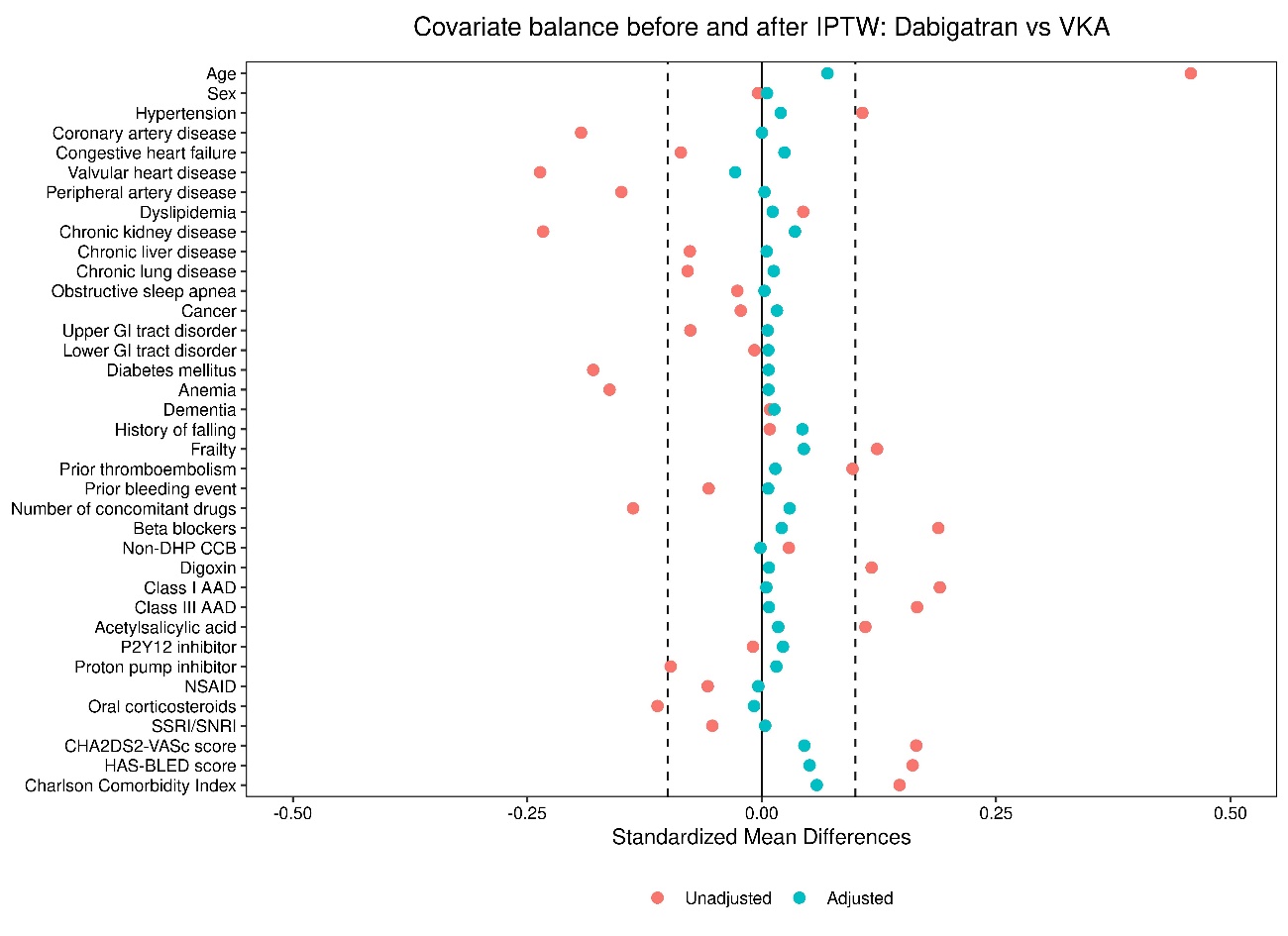
**

**C)**

**
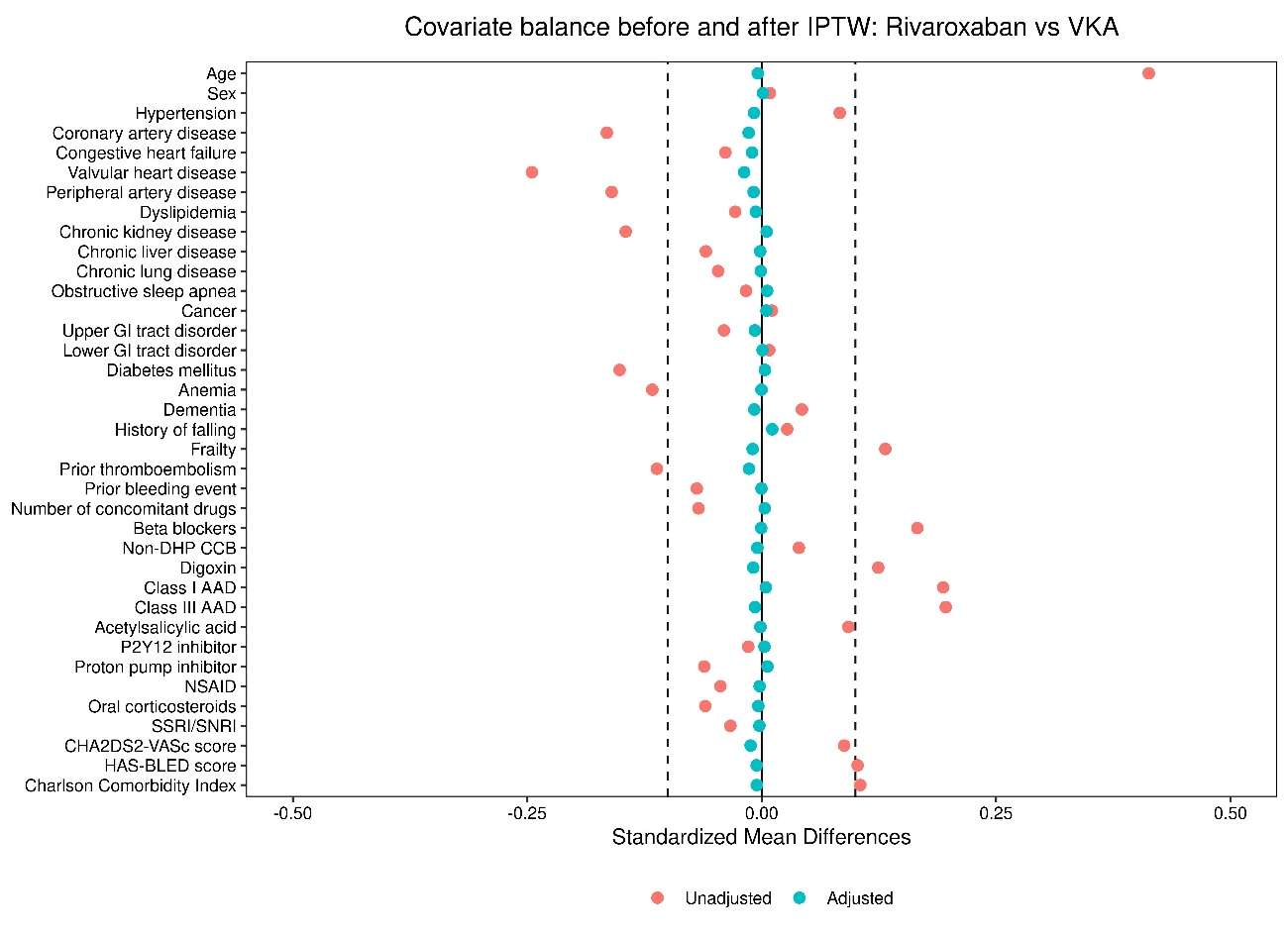
**

**D)**

**
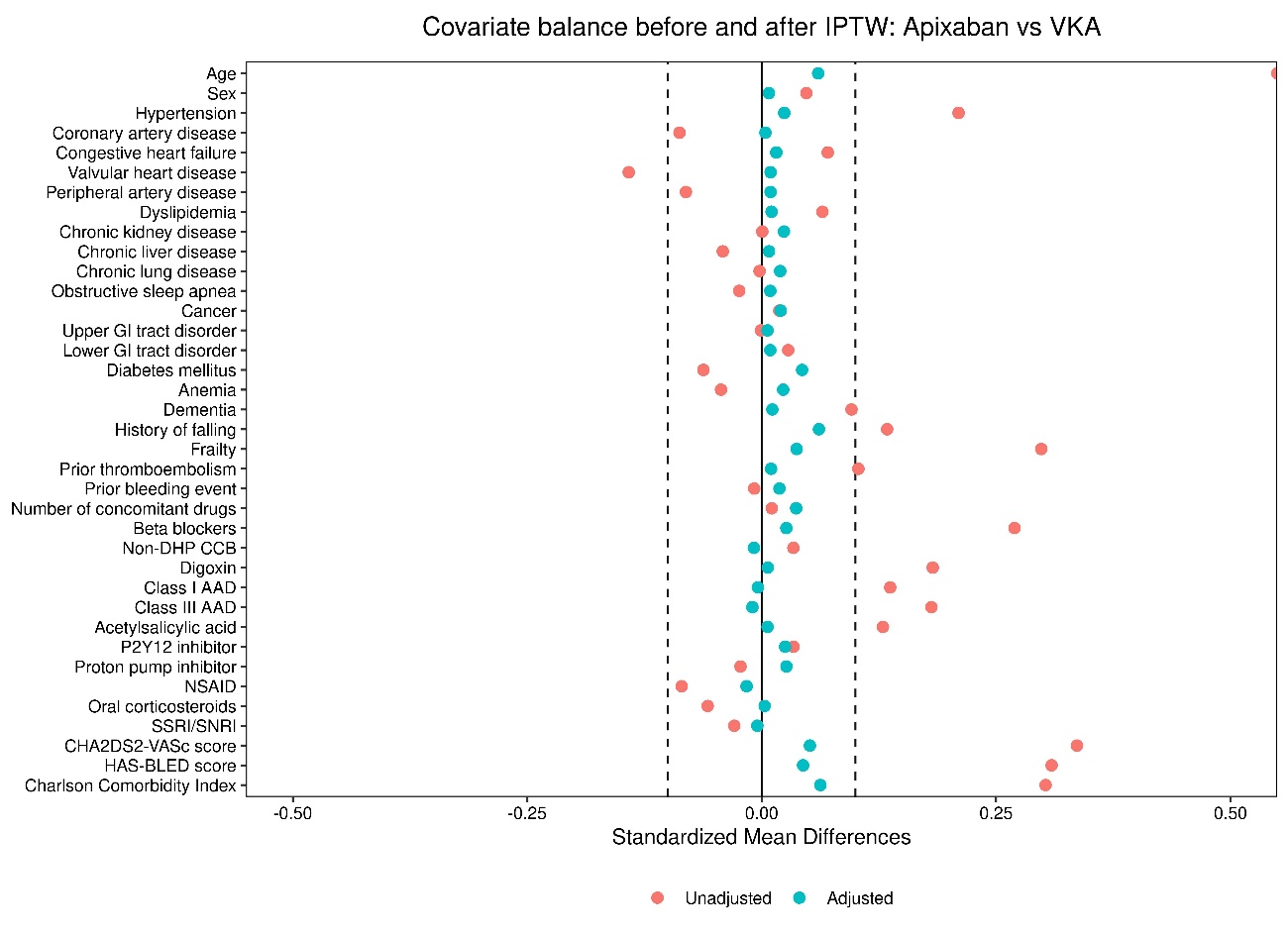
**

**E)**


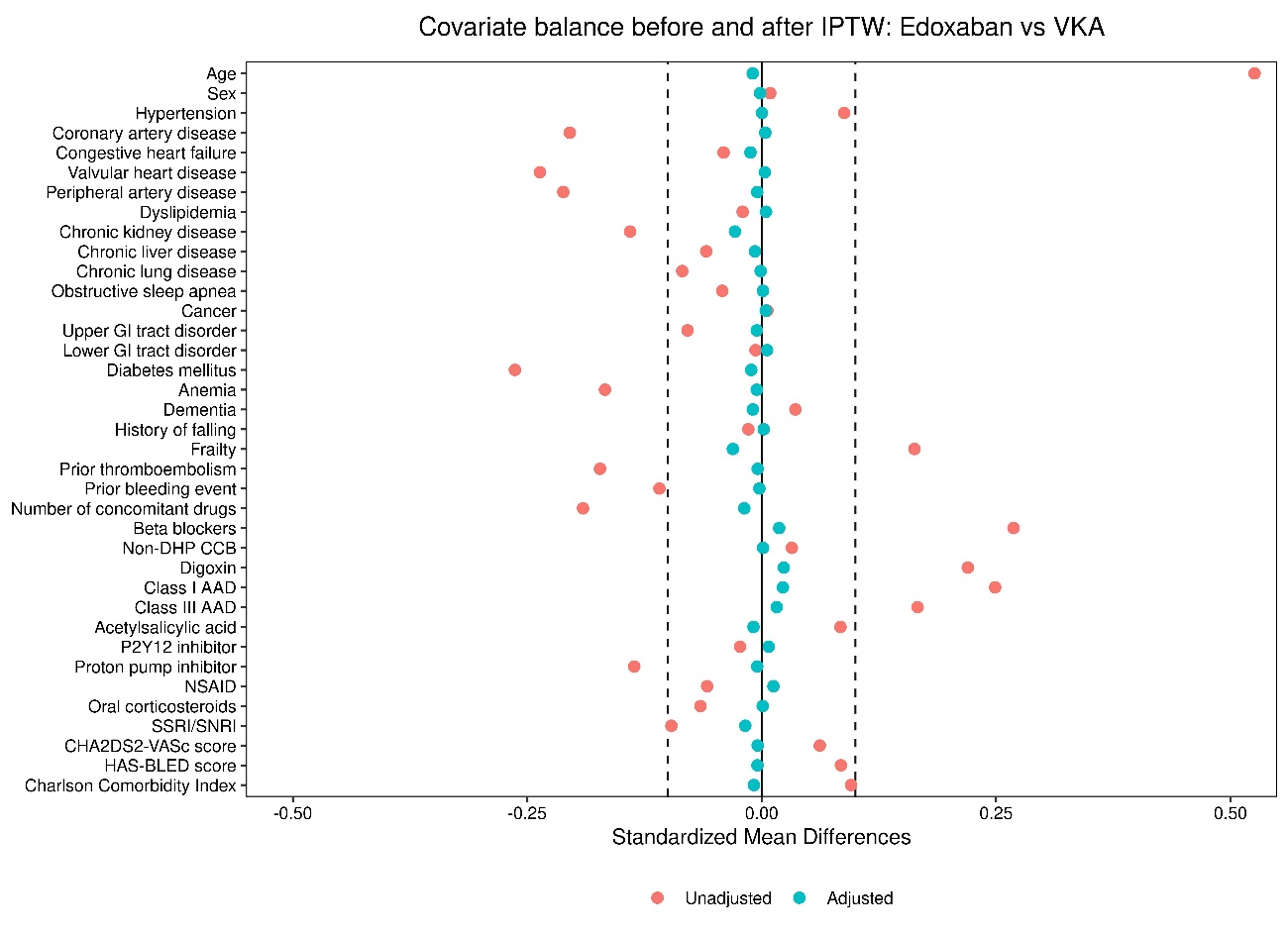


**F)**

**
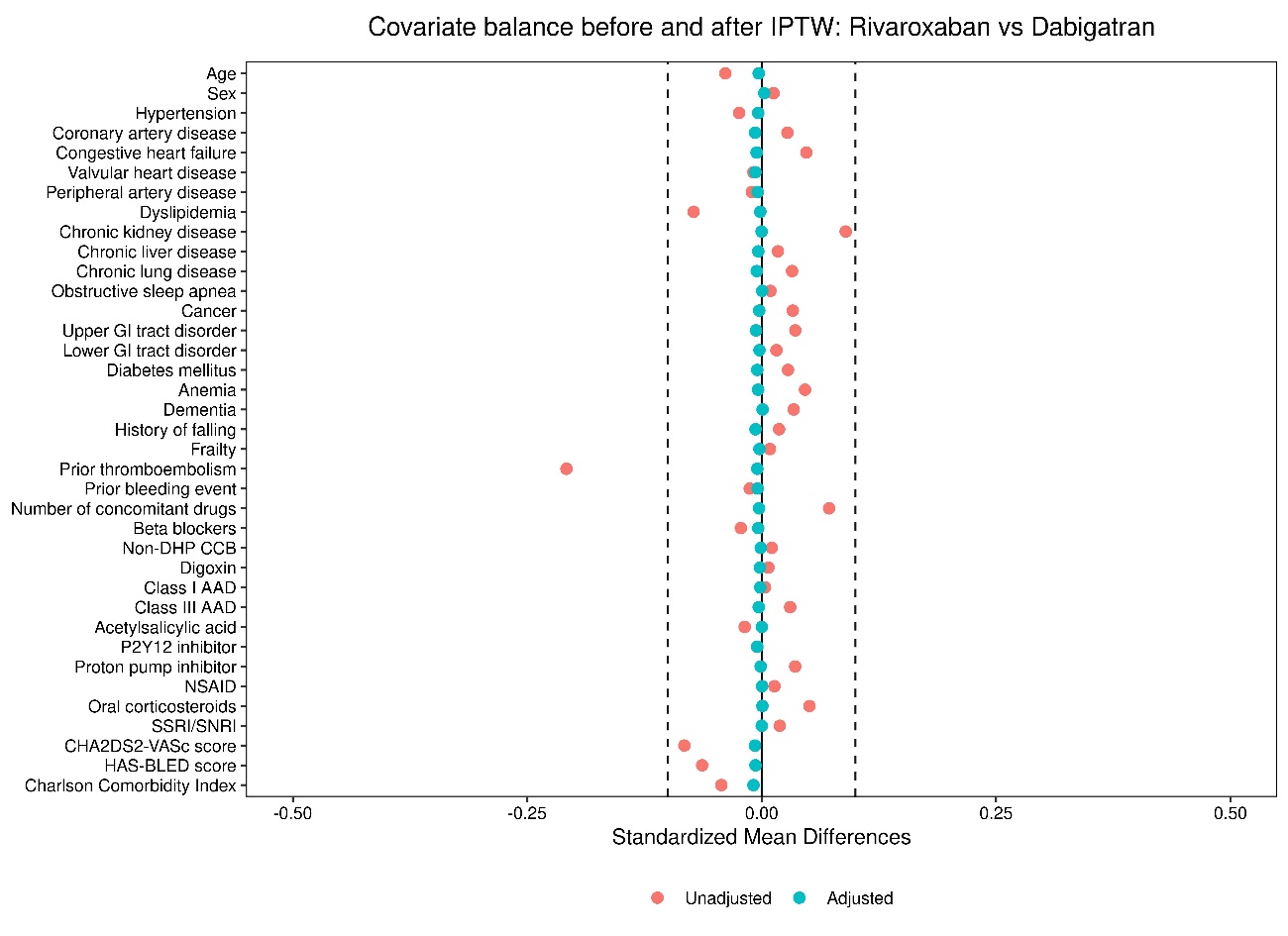
**

**G)**


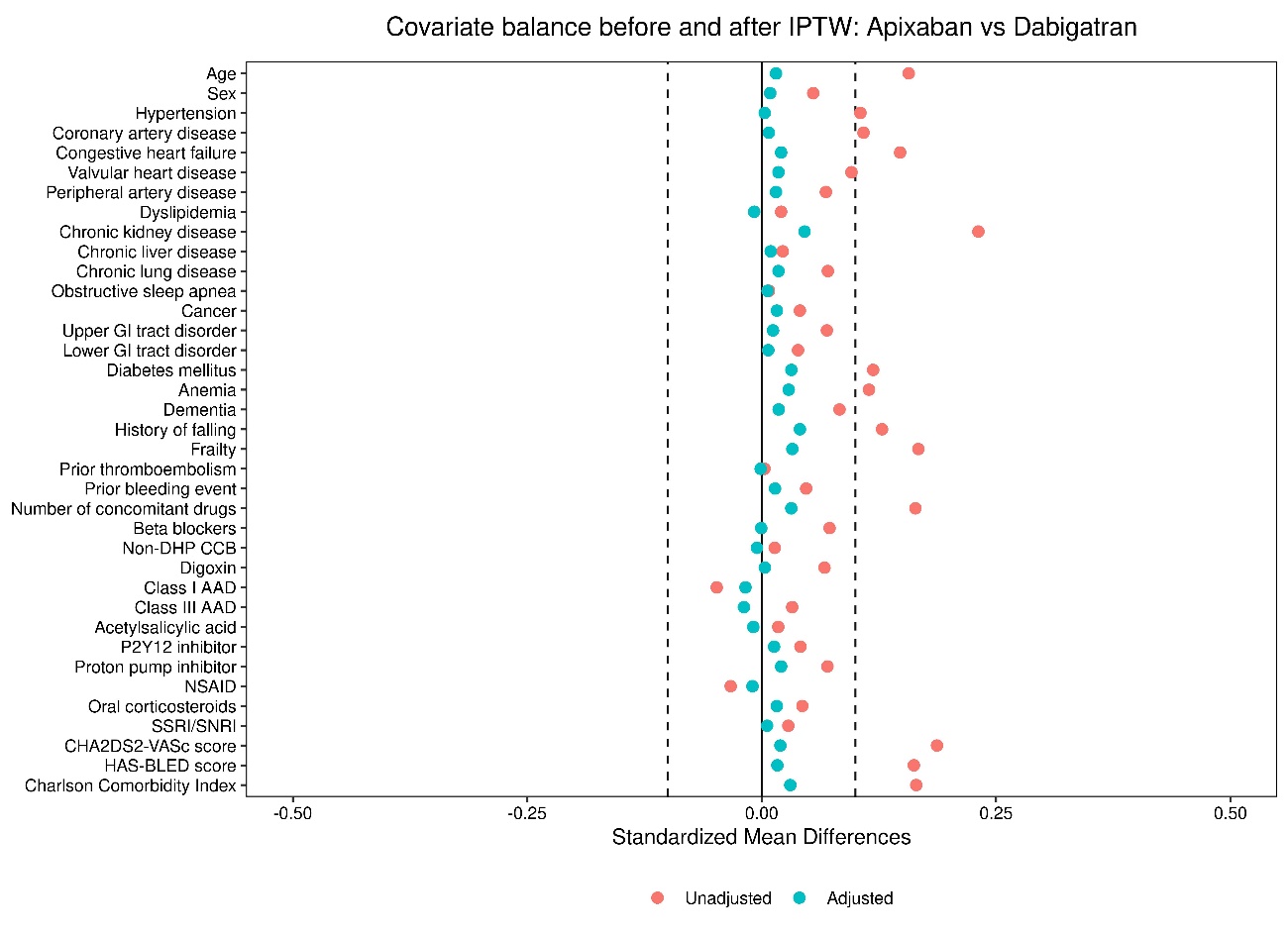


**H)**

**
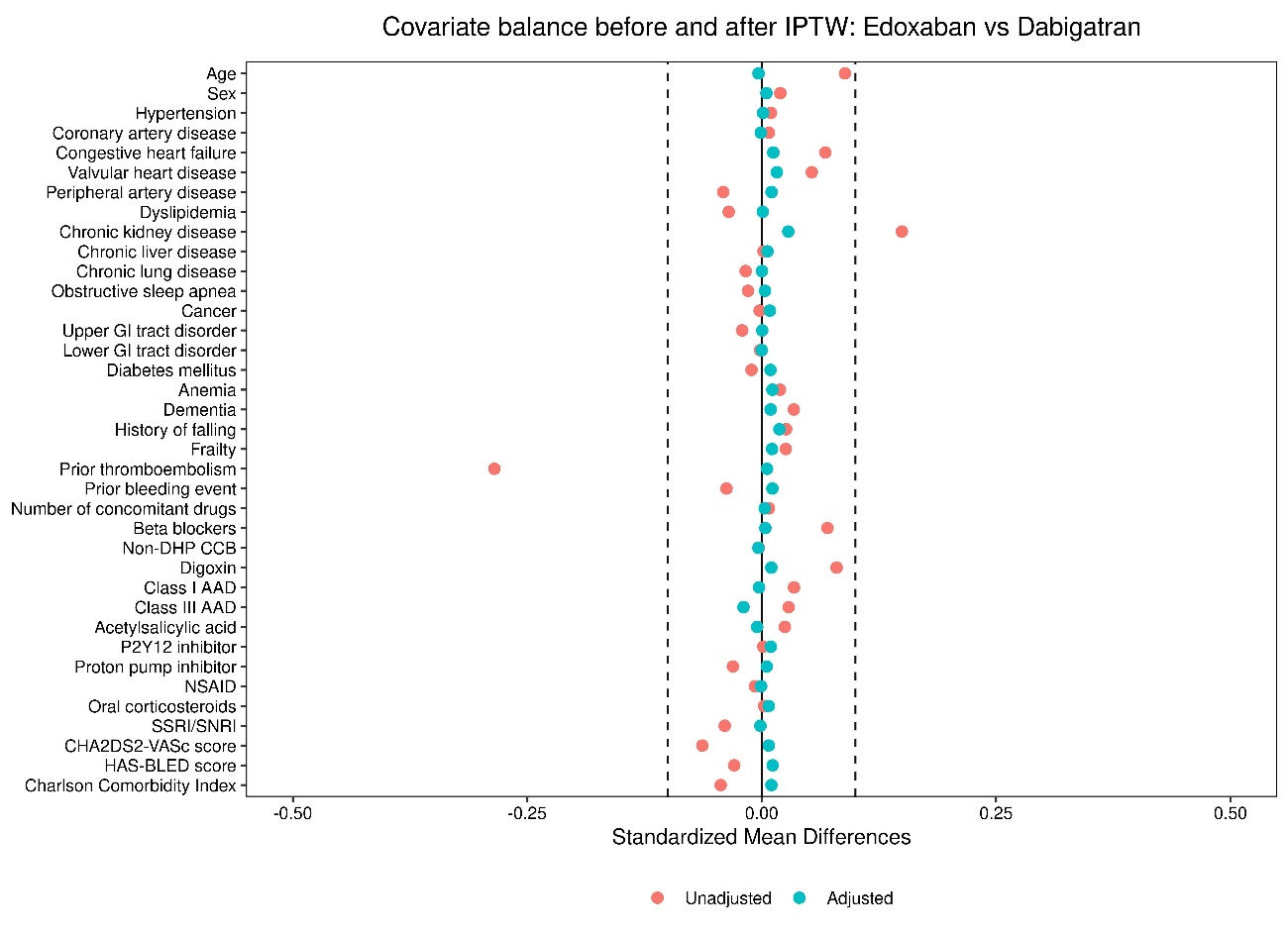
**

**I)**

**
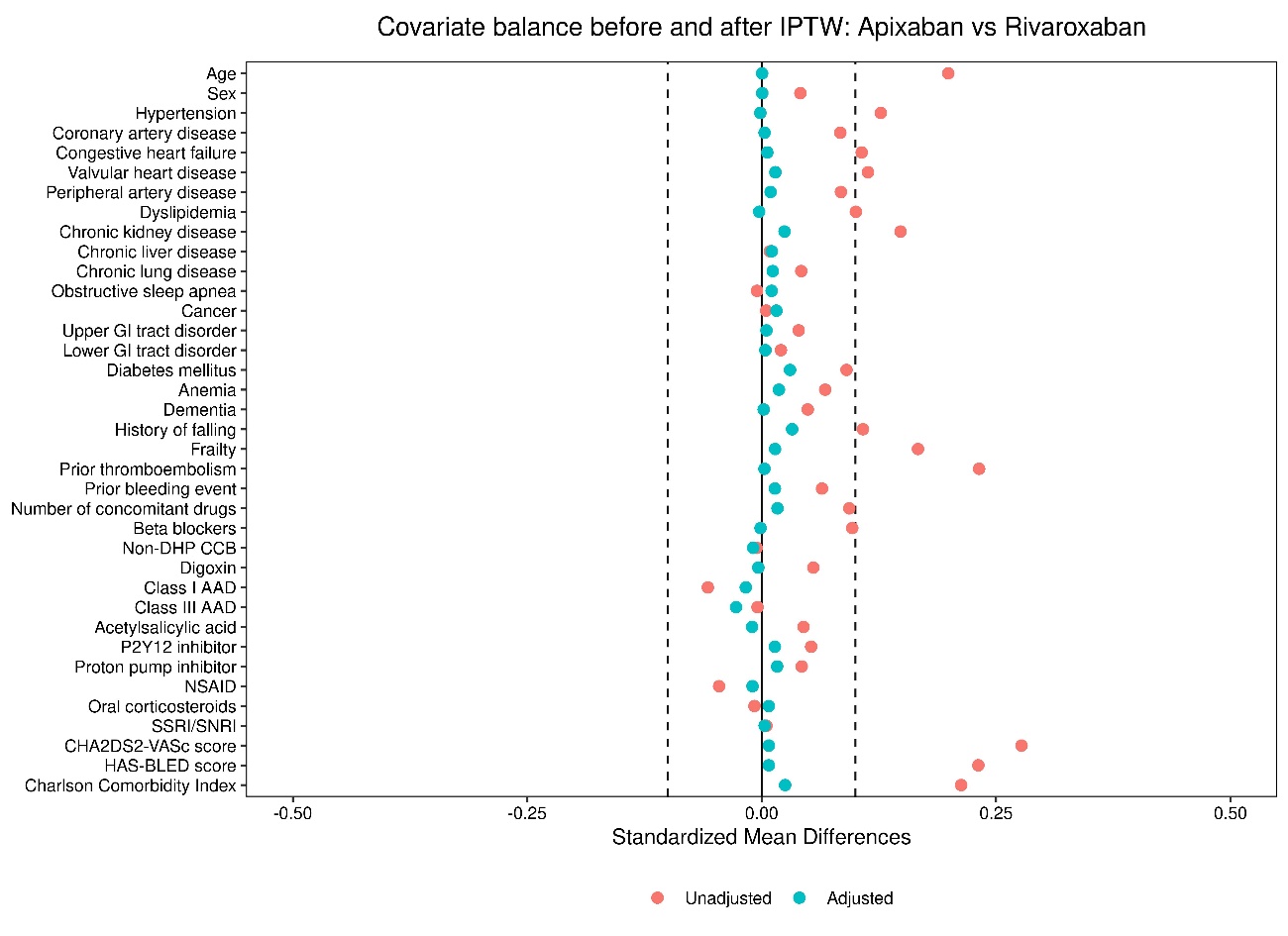
**

**J)**


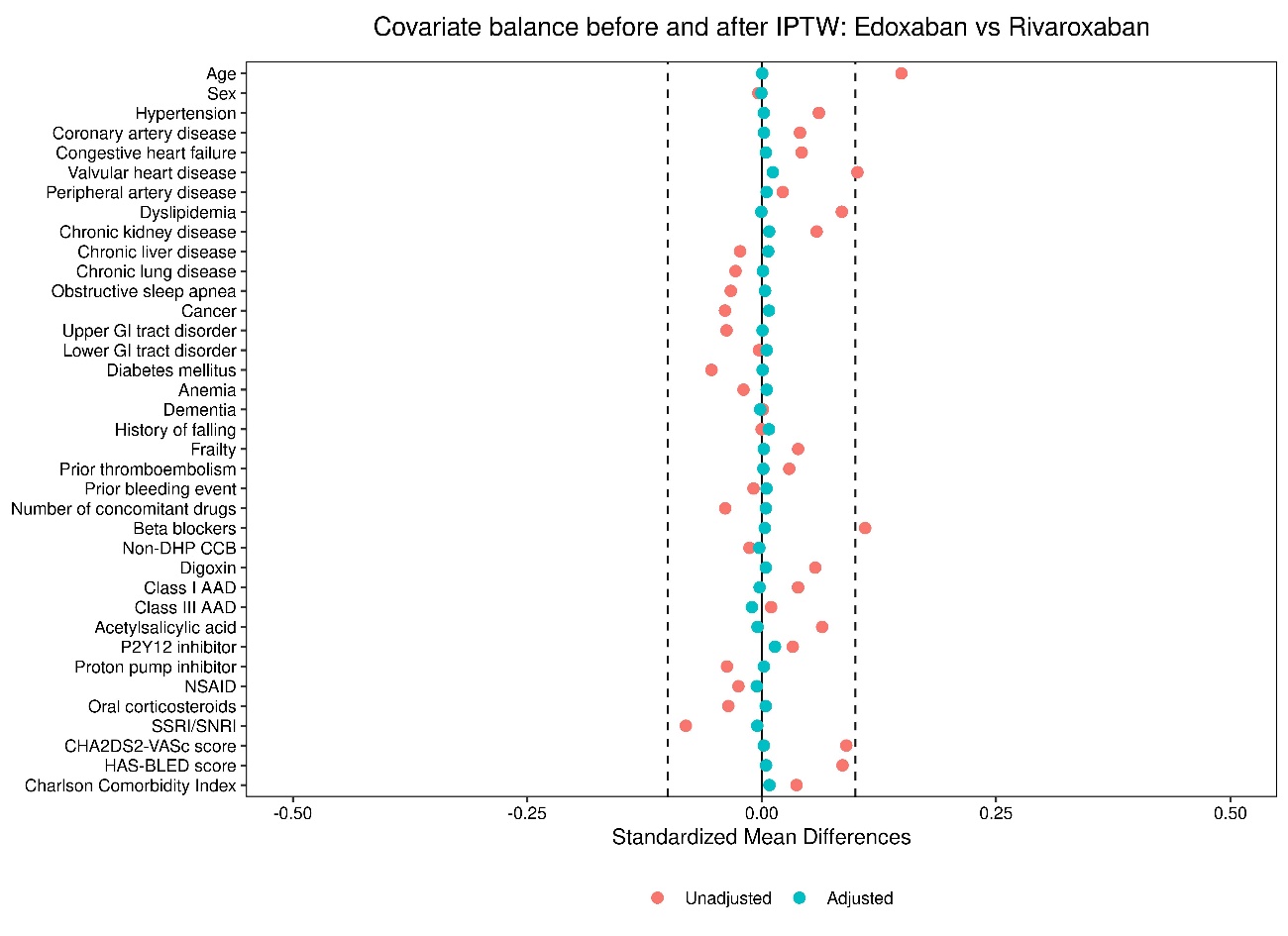


**K)**

**
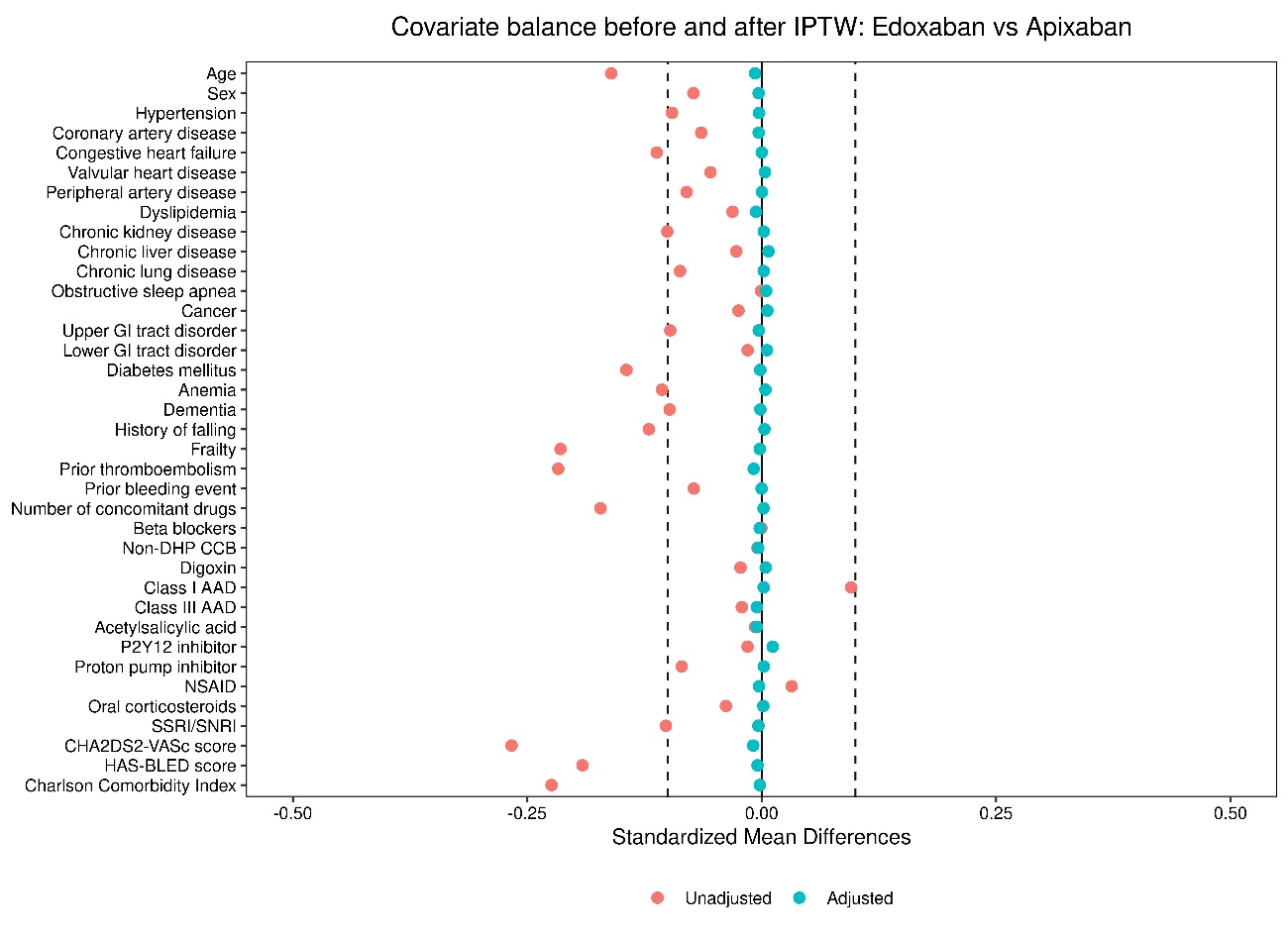
**

**eFigure 2:** Love plots illustrating covariate balance before and after IPTW when comparing **A)** NOACs to VKAs, **B)** dabigatran to VKAs, **C)** rivaroxaban to VKAs, **D)** apixaban to VKAs, **E)** edoxaban to VKAs, **F)** rivaroxaban to dabigatran, **G)** apixaban to dabigatran, **H)** edoxaban to dabigatran, **I)** apixaban to rivaroxaban, **J)** edoxaban to rivaroxaban, and **K)** edoxaban to apixaban.

AAD: antiarrhythmic drug; GI: gastrointestinal; IPTW: inverse probability of treatment weighting; NOAC: non-vitamin K antagonist oral anticoagulant; NSAID: non-steroidal anti-inflammatory drug; OAC: oral anticoagulant; SE: systemic embolism; SNRI: serotonin and norepinephrine reuptake inhibitor; SSRI: selective serotonin reuptake inhibitor; VKA: vitamin K antagonist; vs: versus.

## eFigure 3: Love plots (standard dose NOACs)

**A)**

**
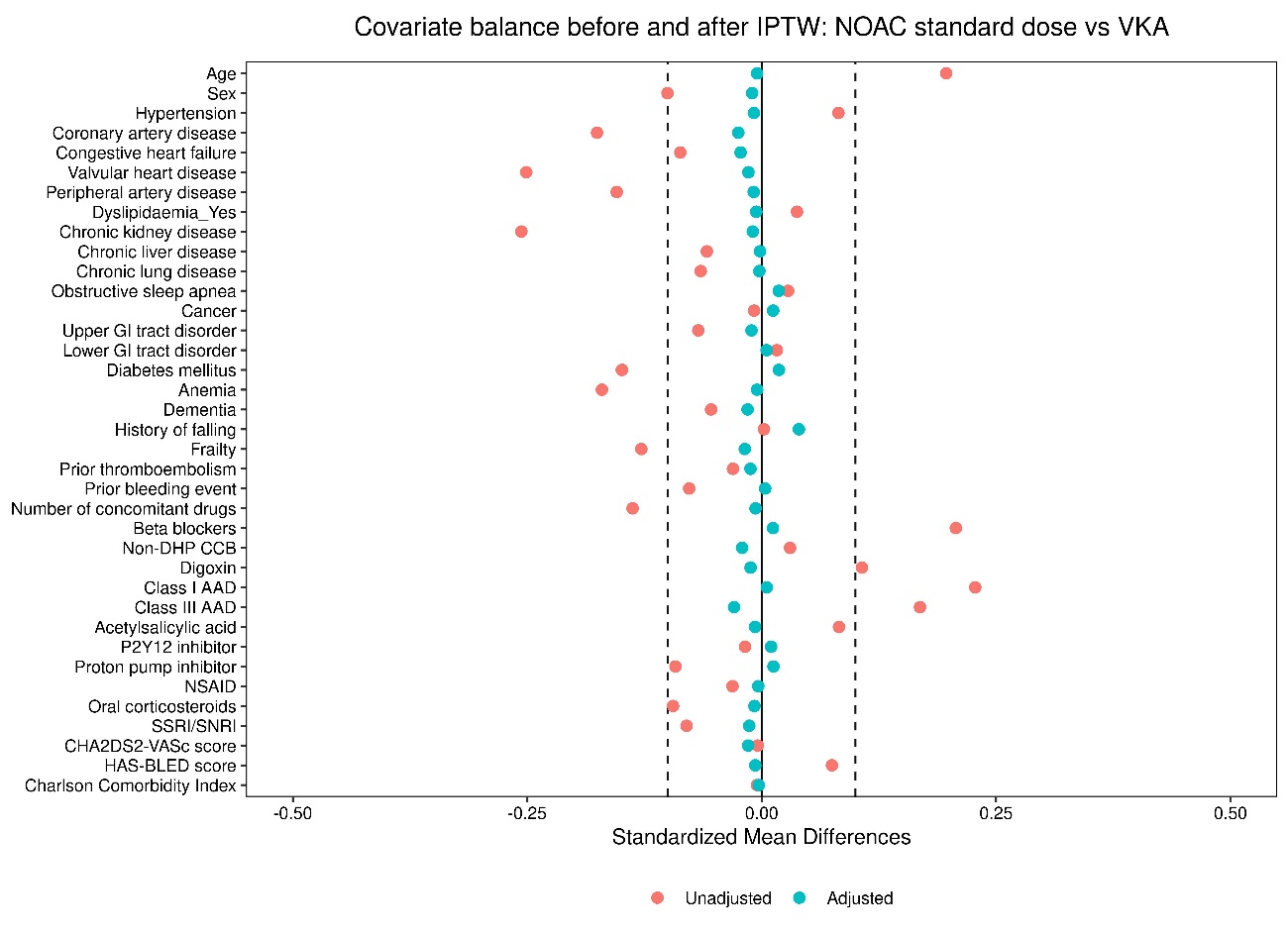
**

**B)**

**
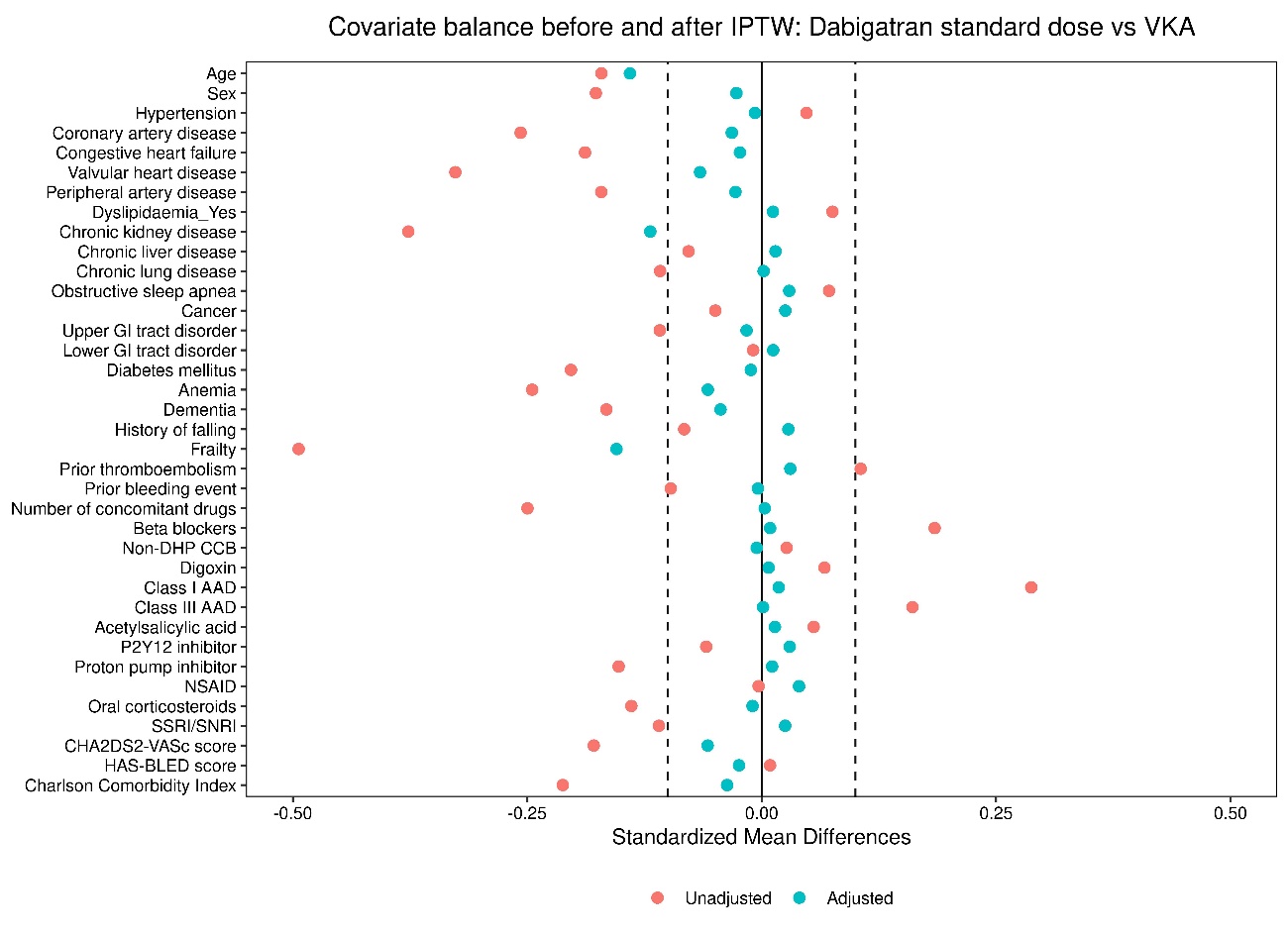
**

**C)**

**
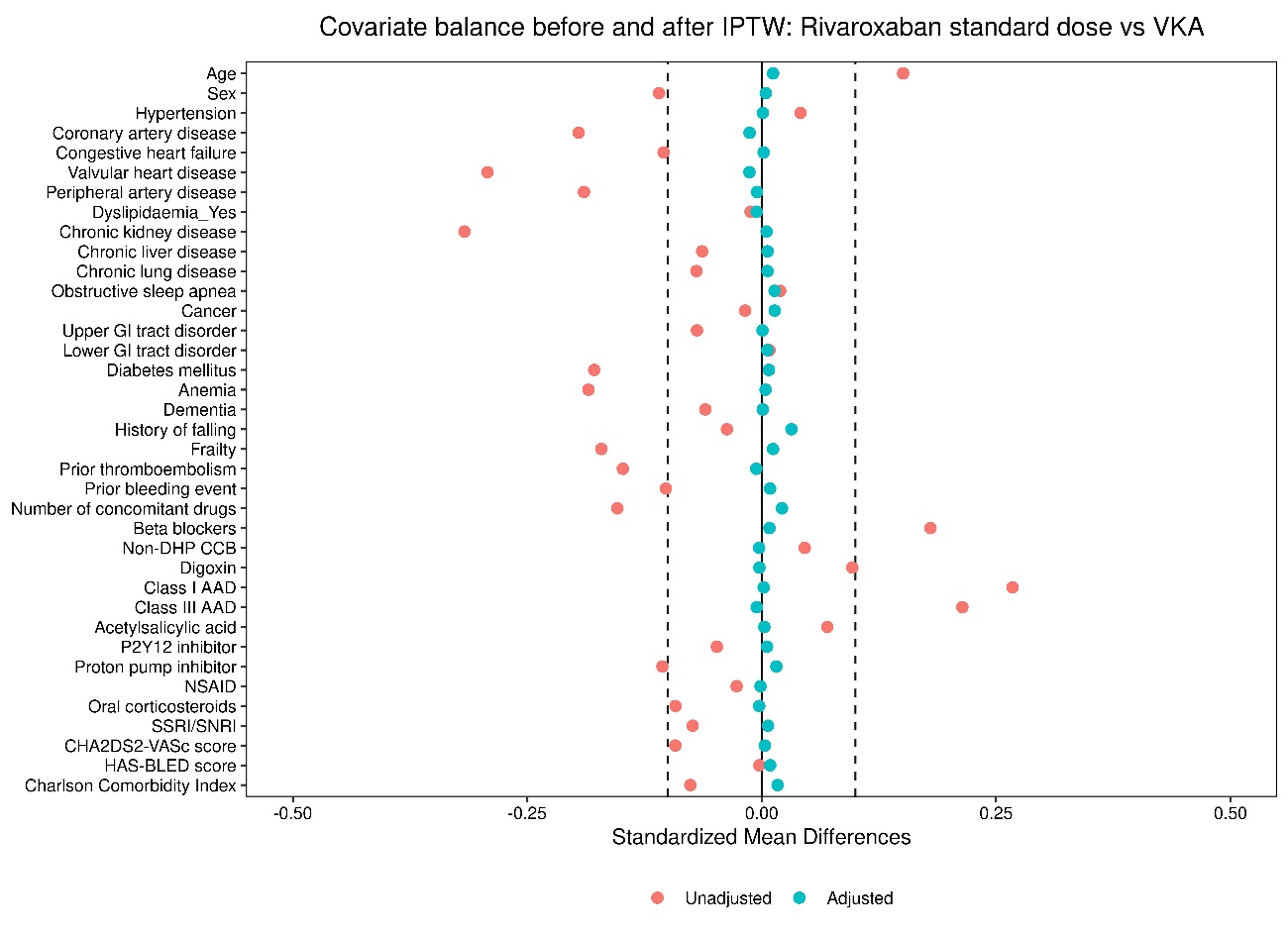
**

**D)**

**
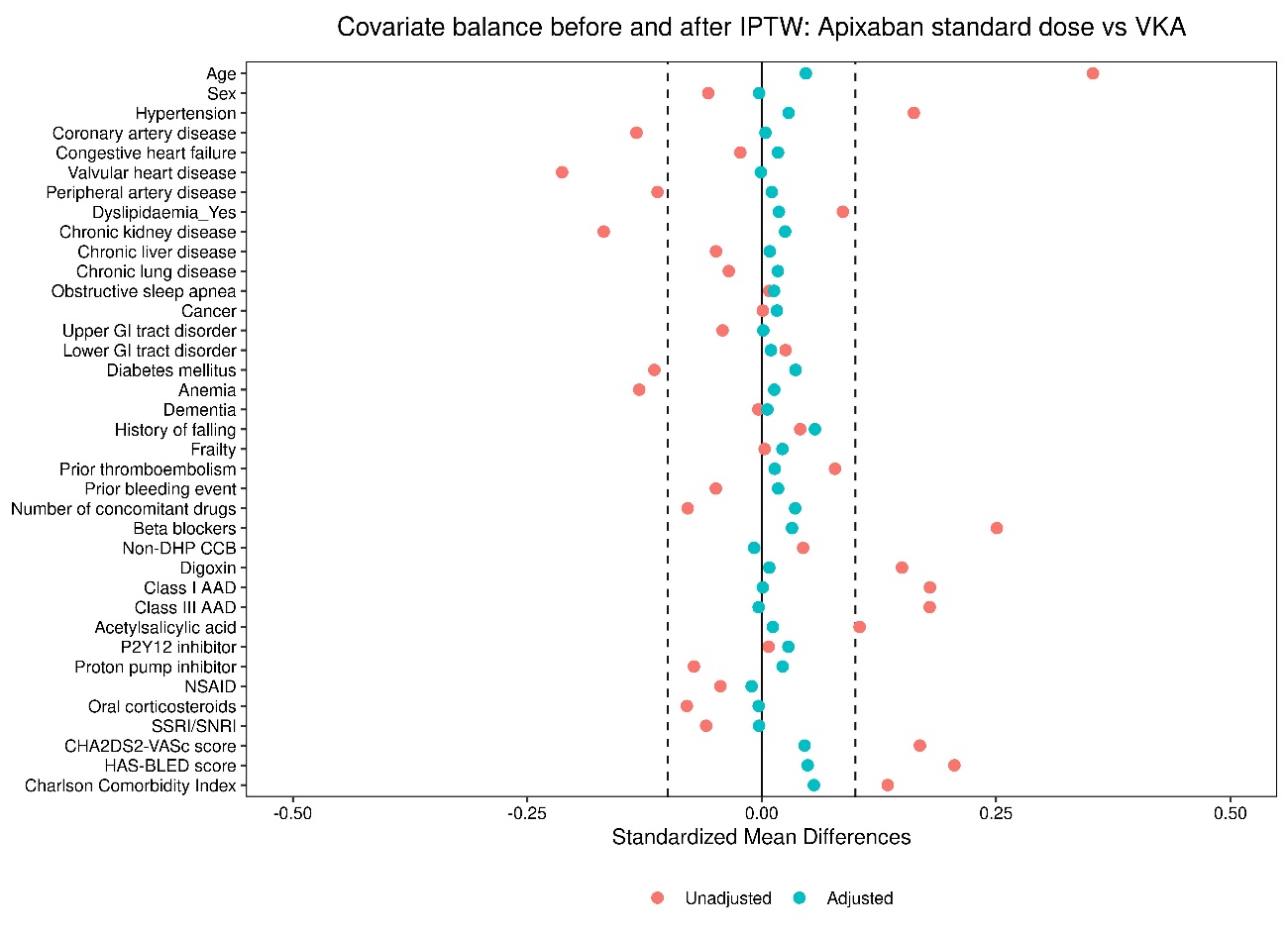
**

**E)**


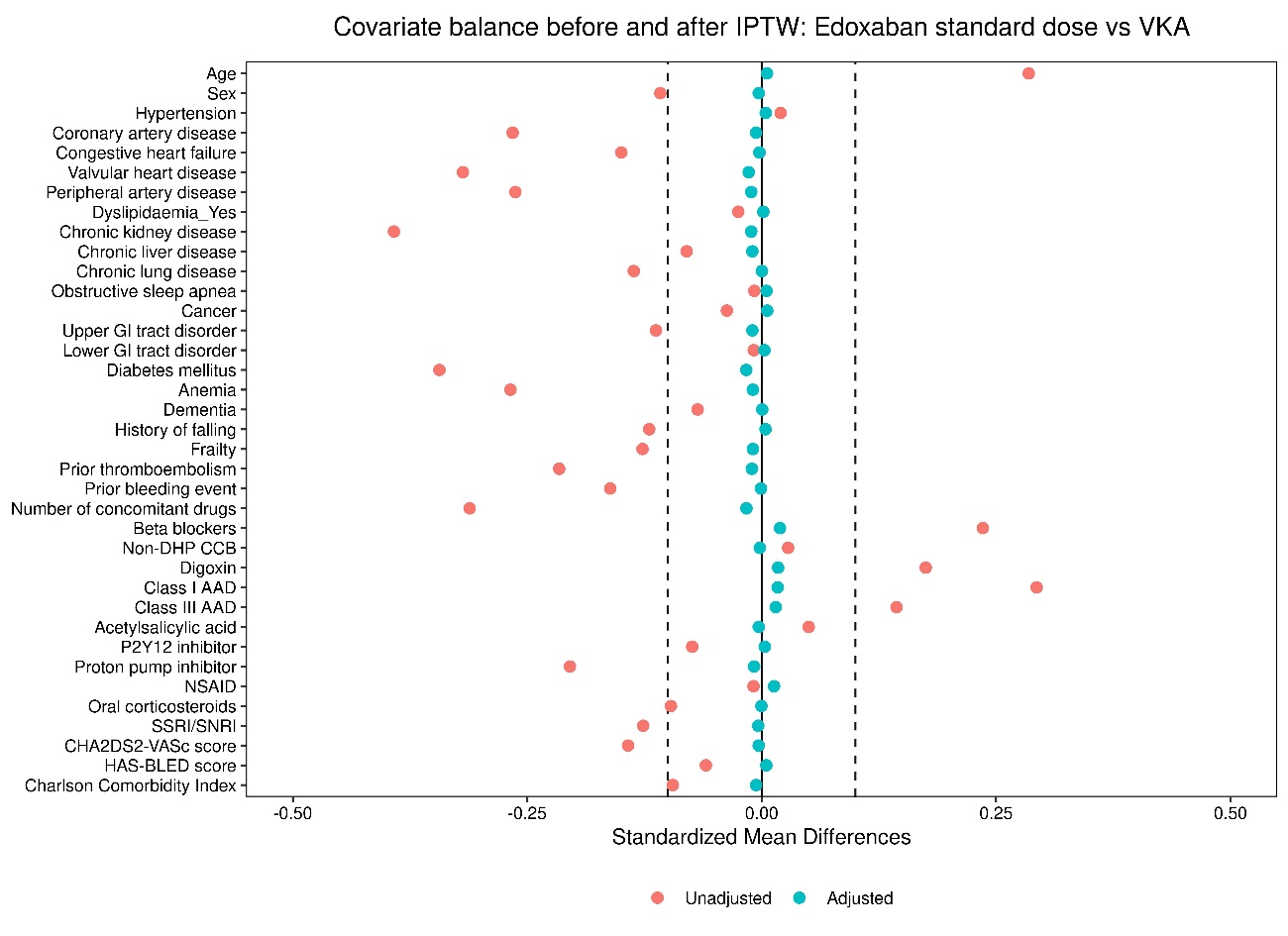


**F)**

**
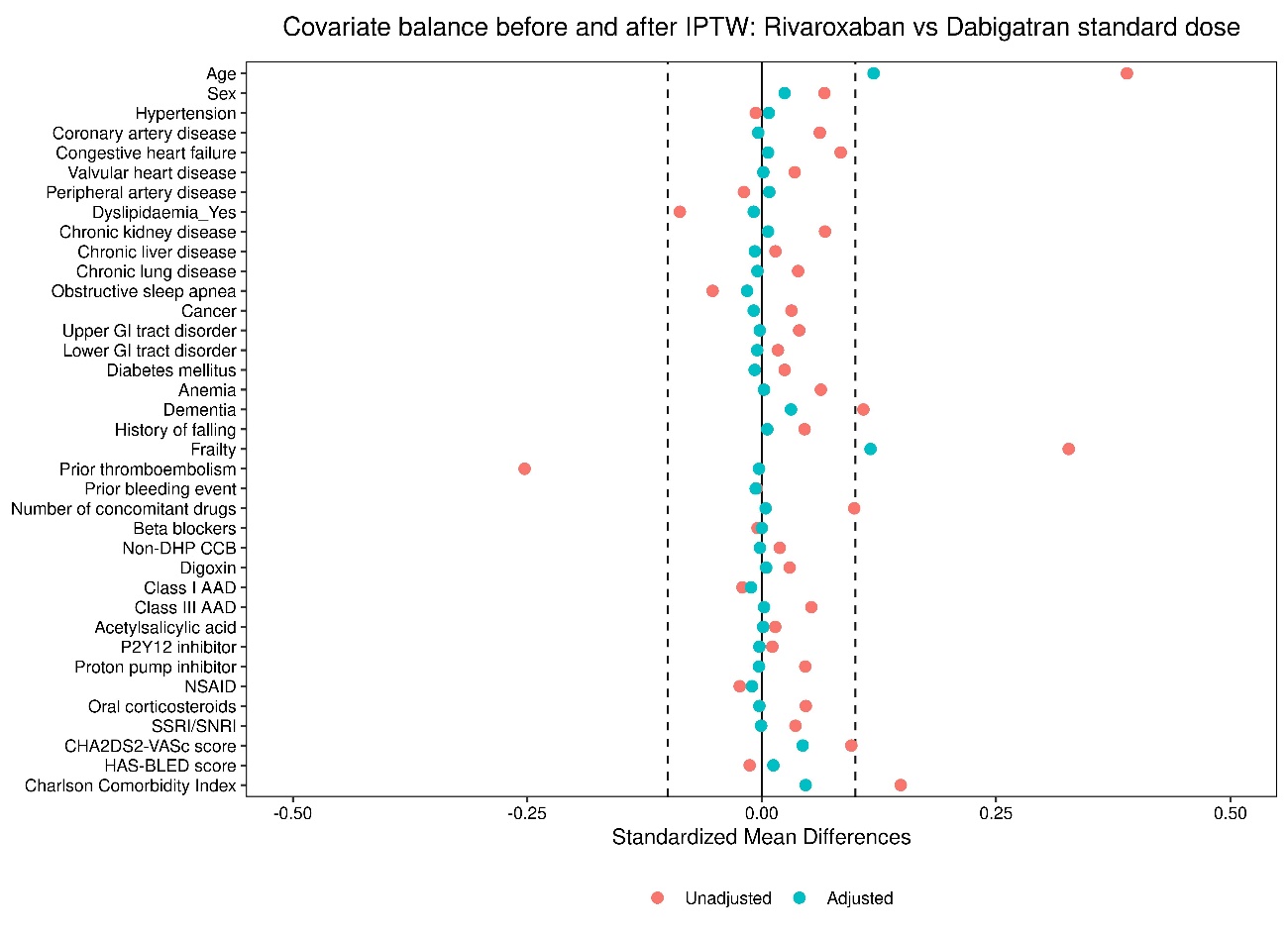
**

**G)**

**
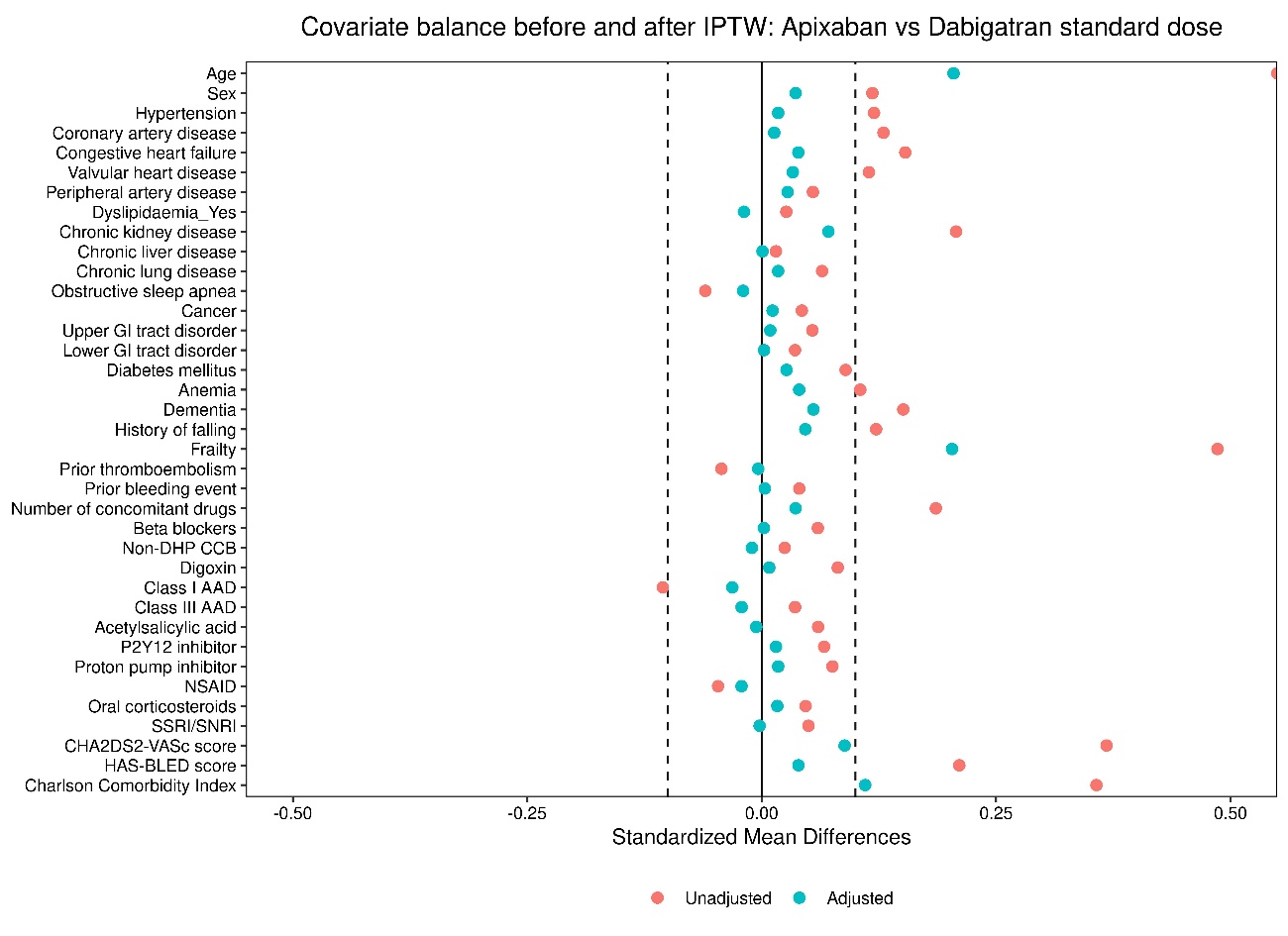
**

**H)**

**
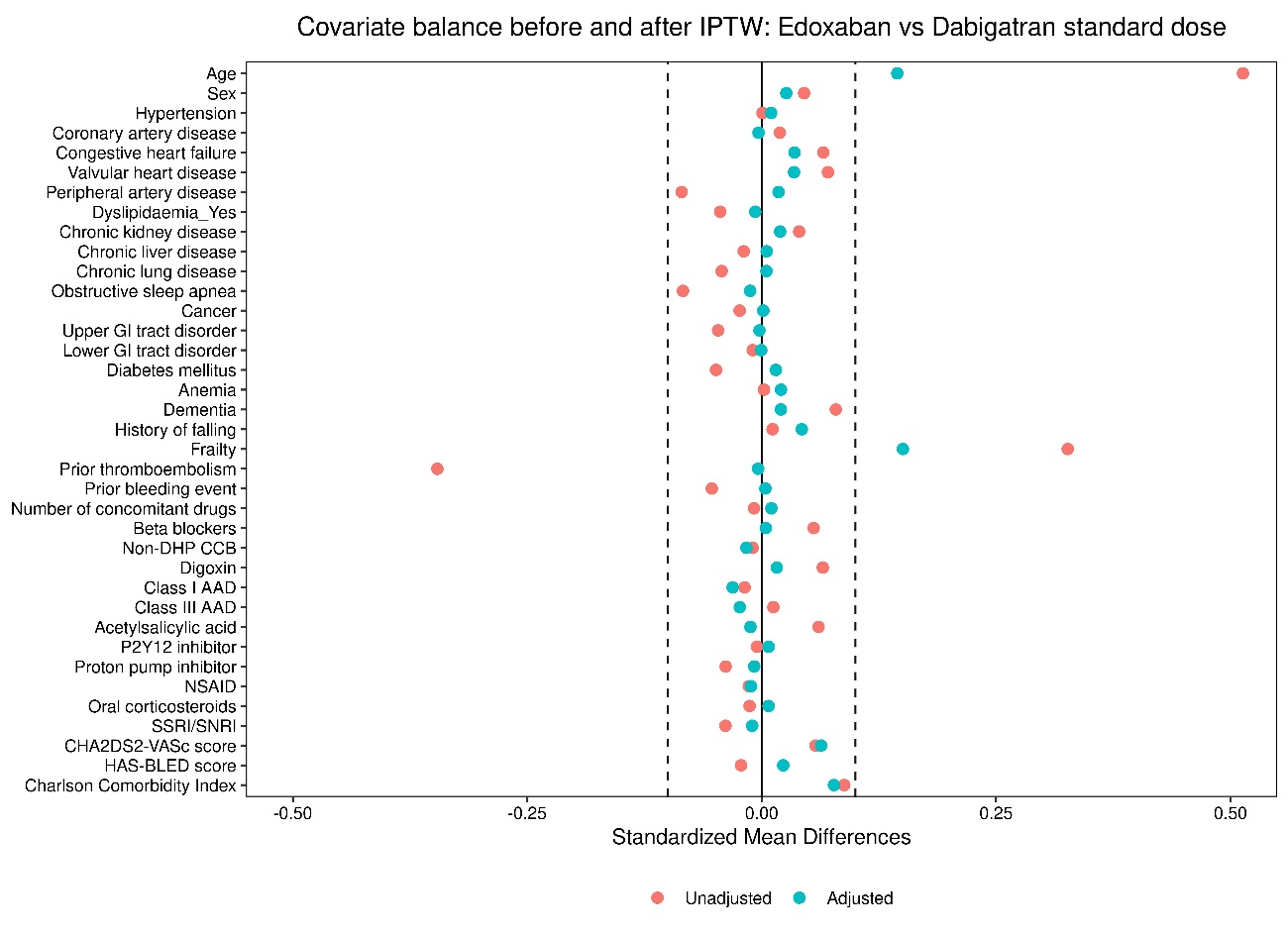
**

**I)**

**
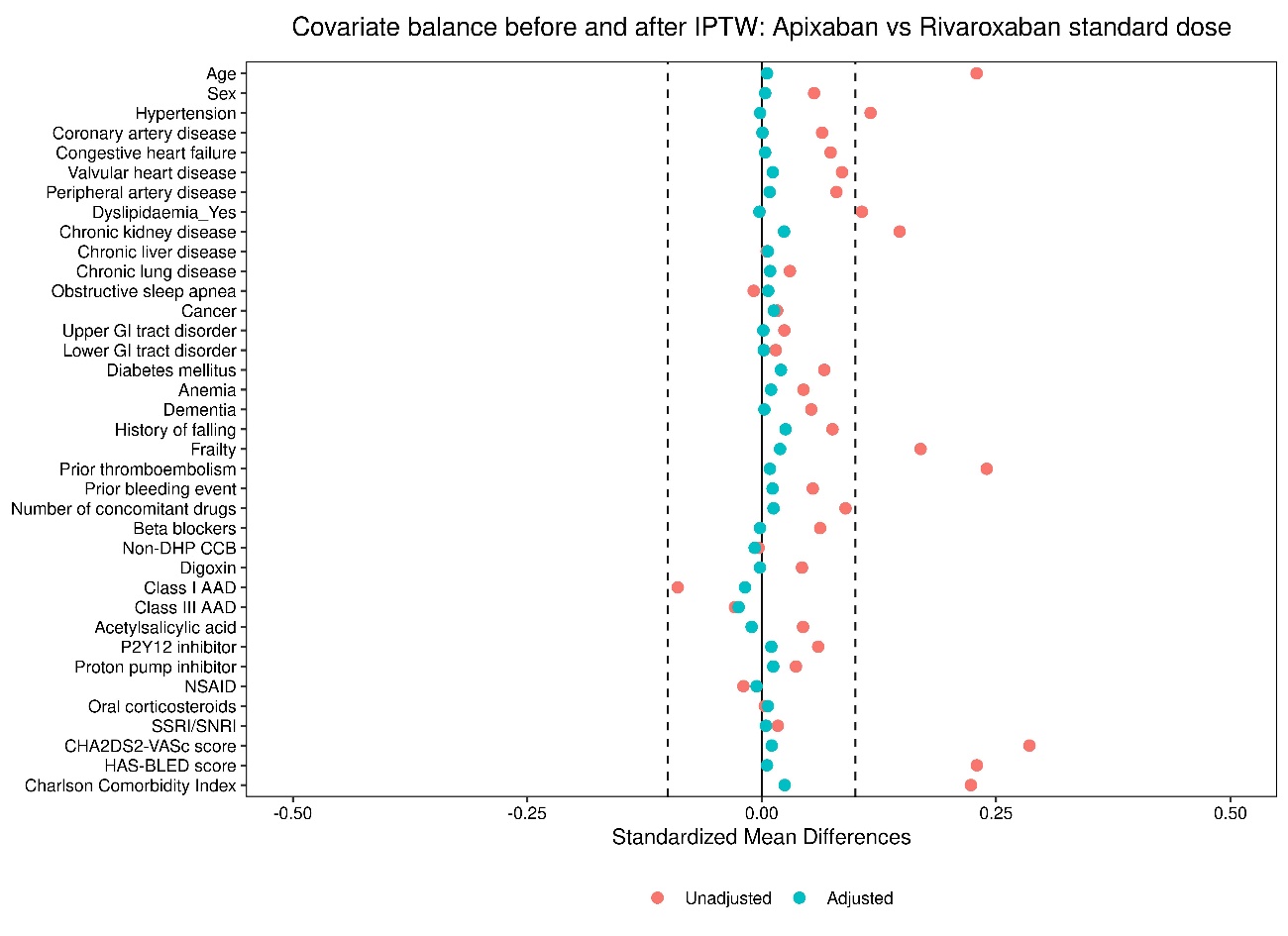
**

**J)**

**
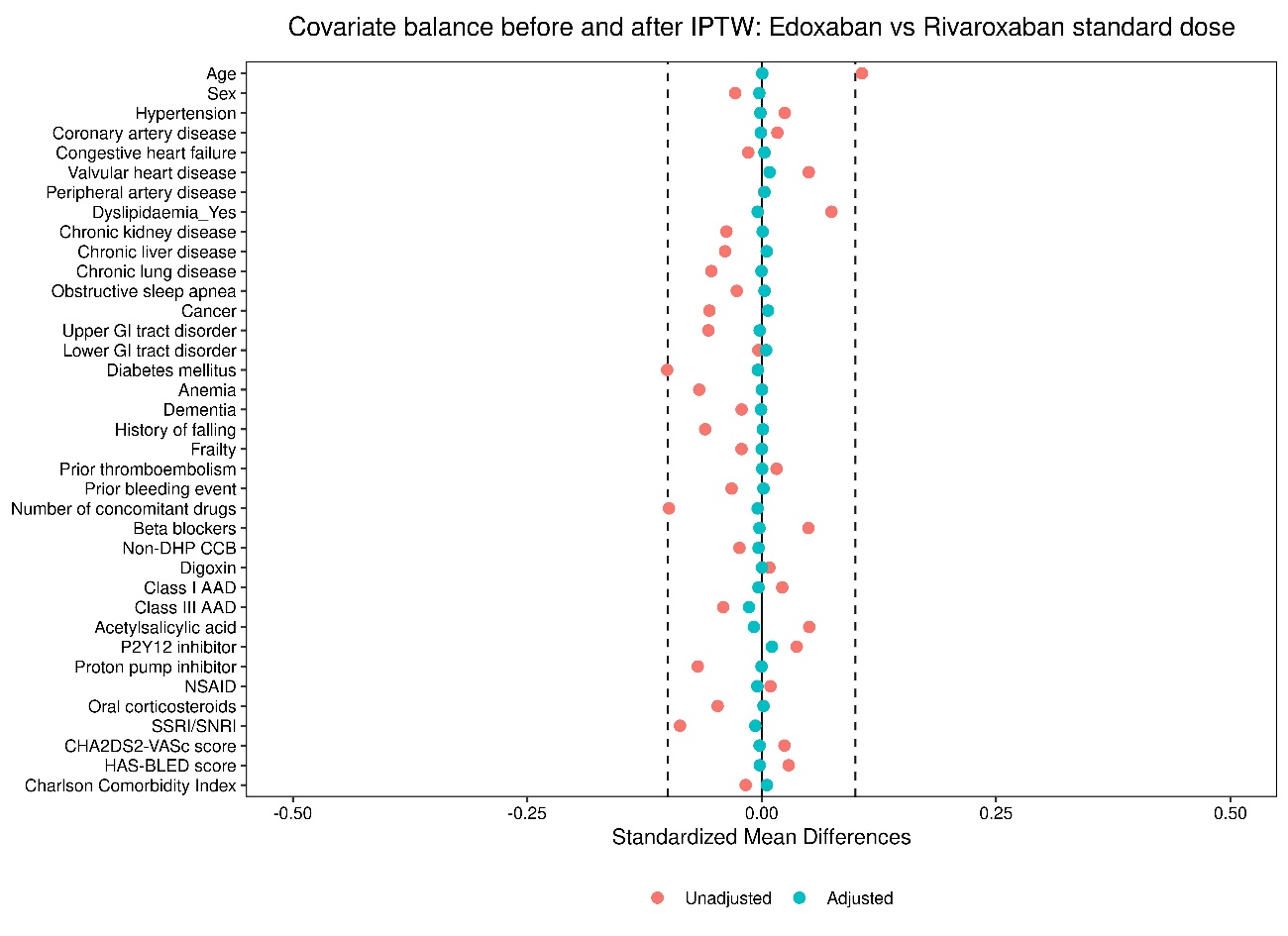
**

**K)**


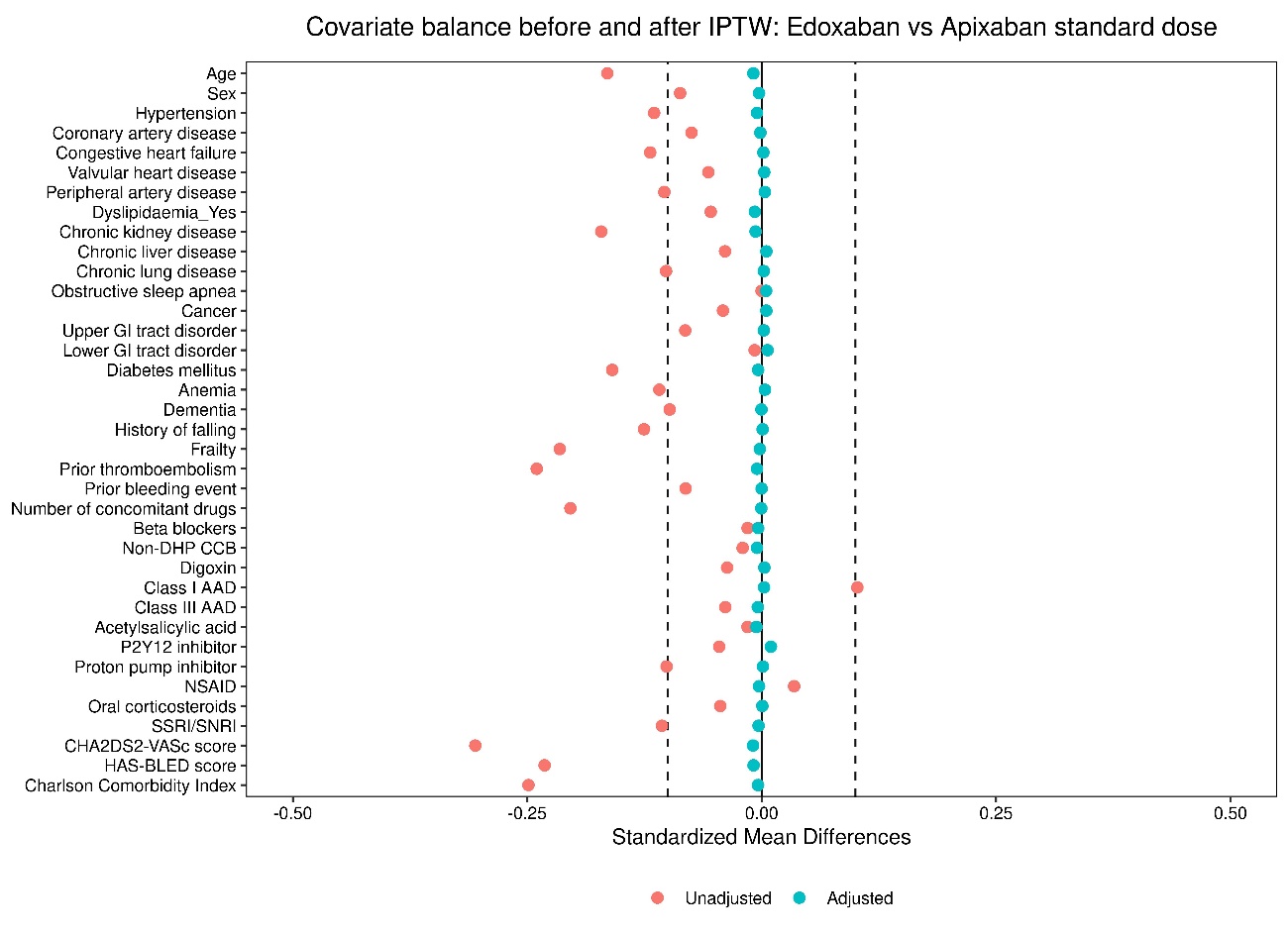


**eFigure 3:** Love plots illustrating covariate balance before and after IPTW when comparing **A)** standard dose NOACs to VKAs, **B)** standard dose dabigatran to VKAs, **C)** standard dose rivaroxaban to VKAs, **D)** standard dose apixaban to VKAs, **E)** standard dose edoxaban to VKAs, **F)** standard dose rivaroxaban to dabigatran, **G)** standard dose apixaban to dabigatran, **H)** standard dose edoxaban to dabigatran, **I)** standard dose apixaban to rivaroxaban, **J)** standard dose edoxaban to rivaroxaban, and **K)** standard dose edoxaban to apixaban.

AAD: antiarrhythmic drug; GI: gastrointestinal; IPTW: inverse probability of treatment weighting; NOAC: non-vitamin K antagonist oral anticoagulant; NSAID: non-steroidal anti-inflammatory drug; OAC: oral anticoagulant; SE: systemic embolism; SNRI: serotonin and norepinephrine reuptake inhibitor; SSRI: selective serotonin reuptake inhibitor; VKA: vitamin K antagonist; vs: versus.

## eFigure 4: Love plots (reduced dose NOACs)

**A)**

**
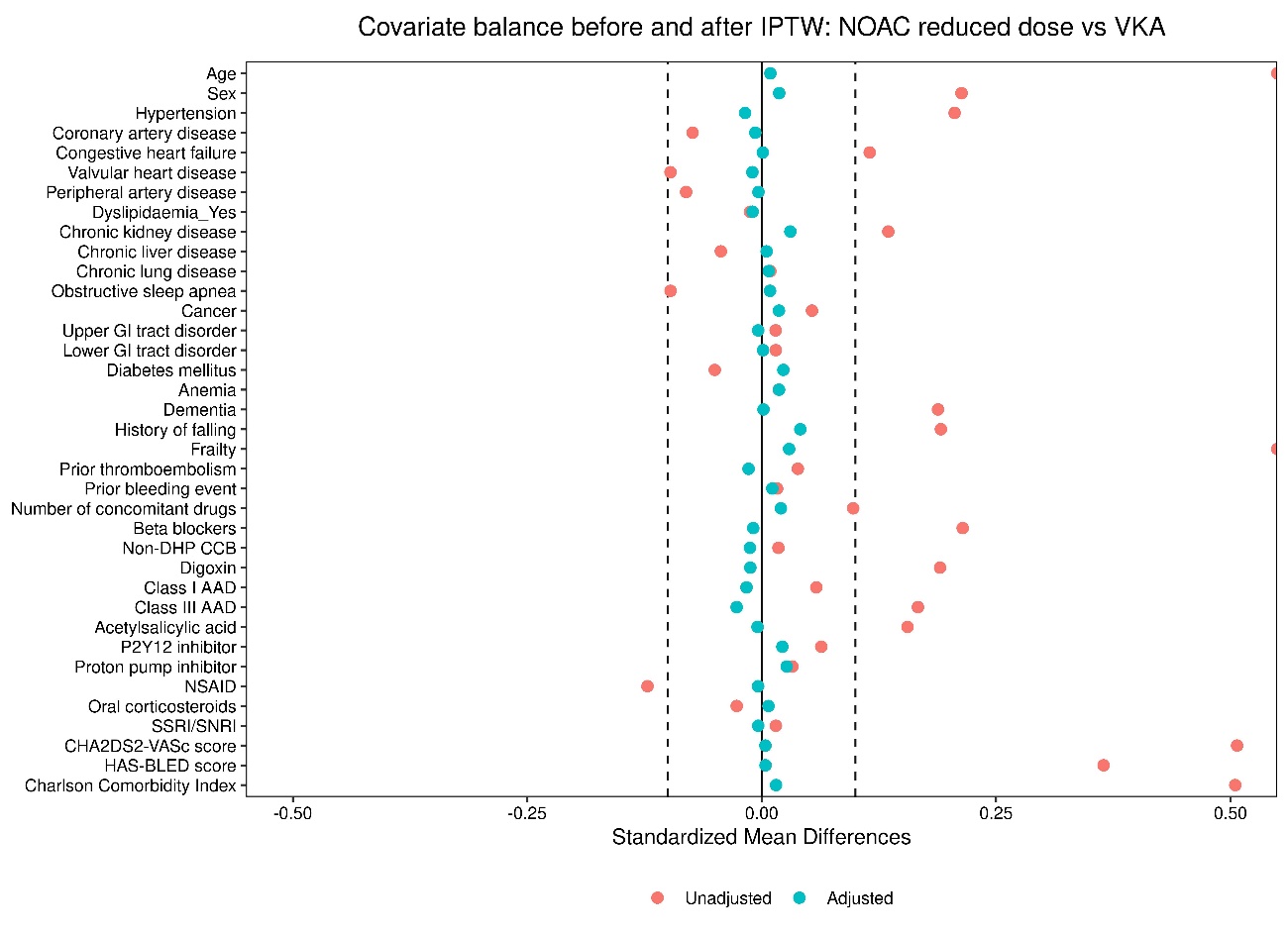
**

**B)**

**
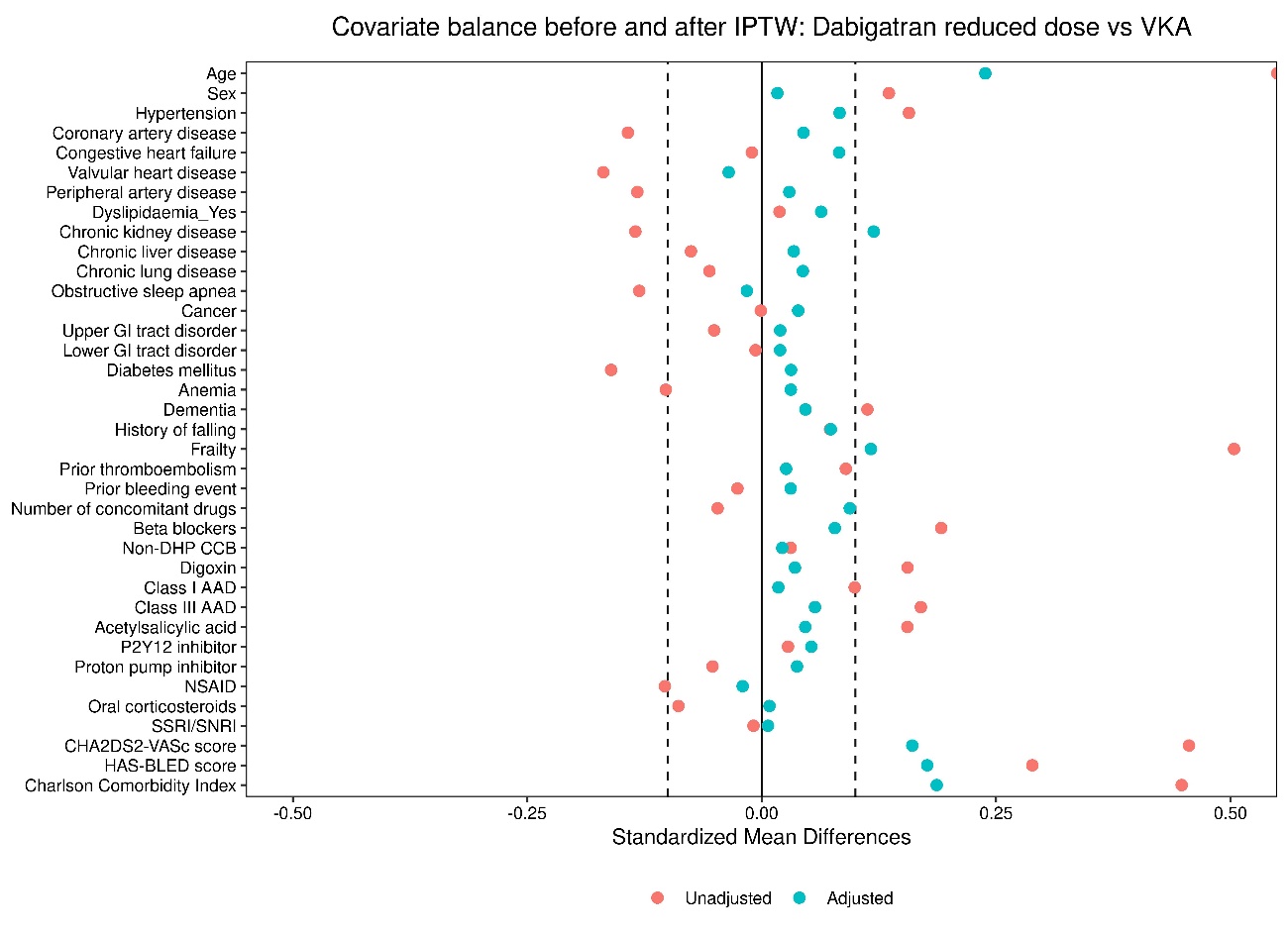
**

**C)**

**
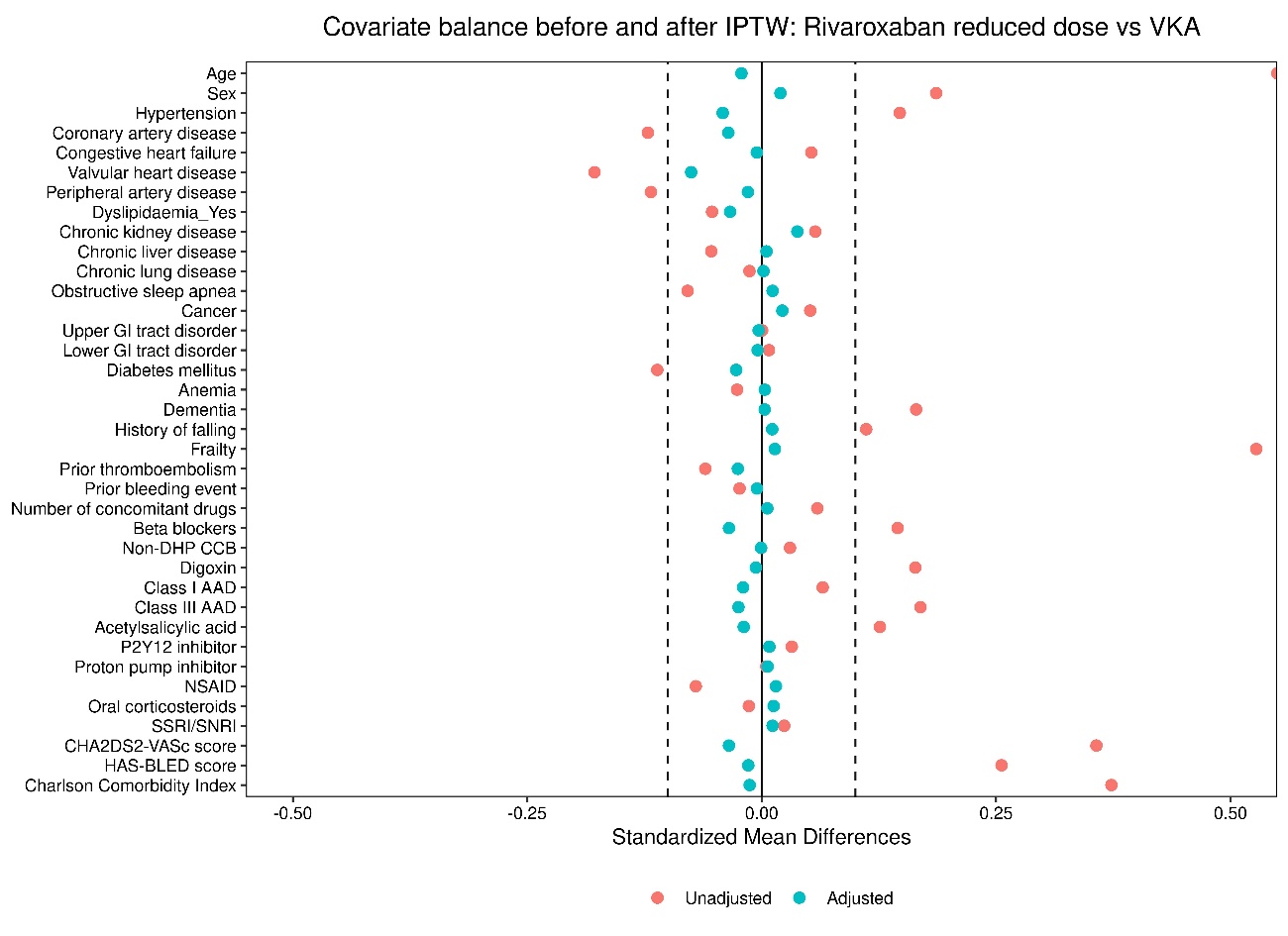
**

**D)**

**
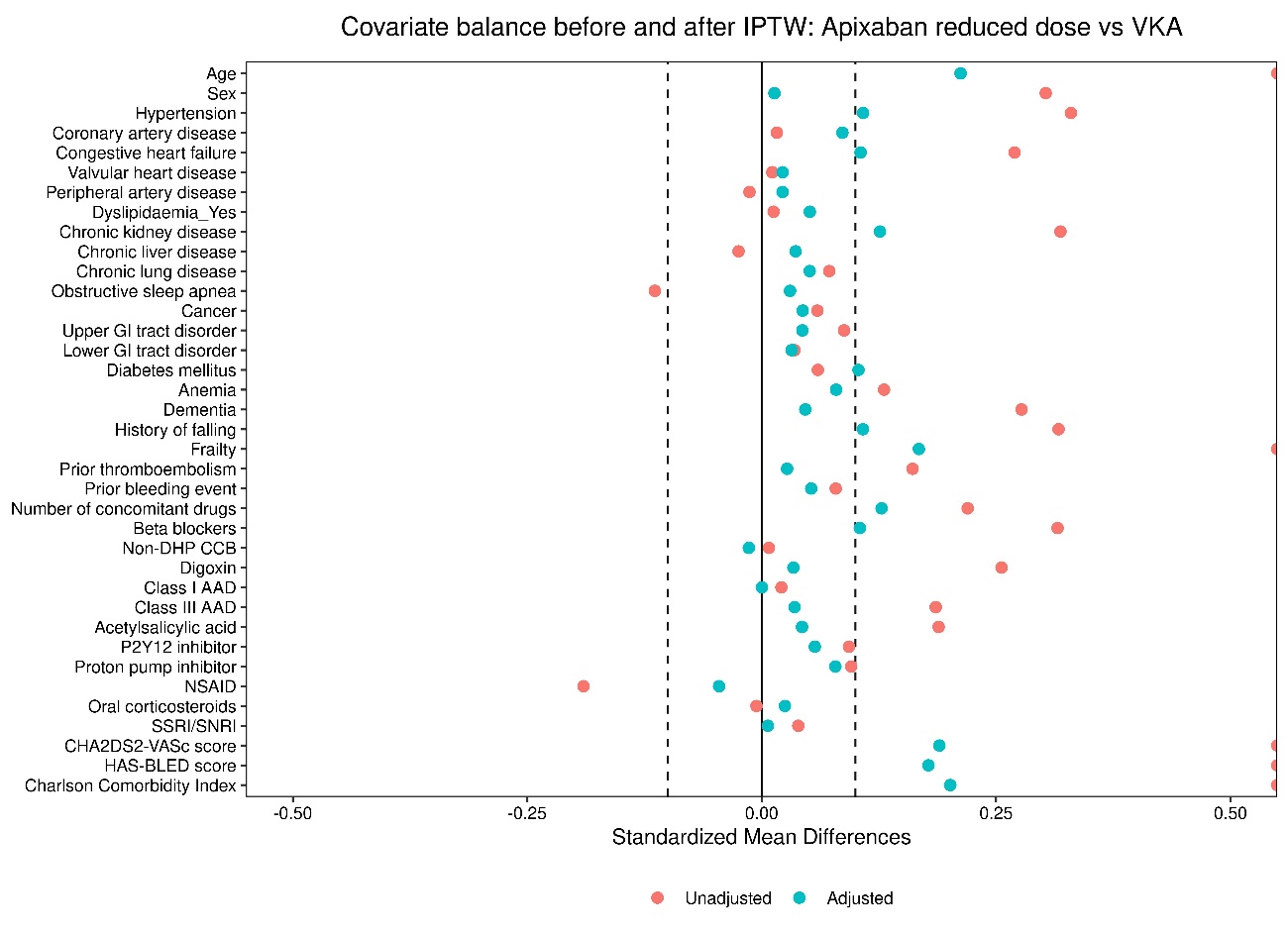
**

**E)**


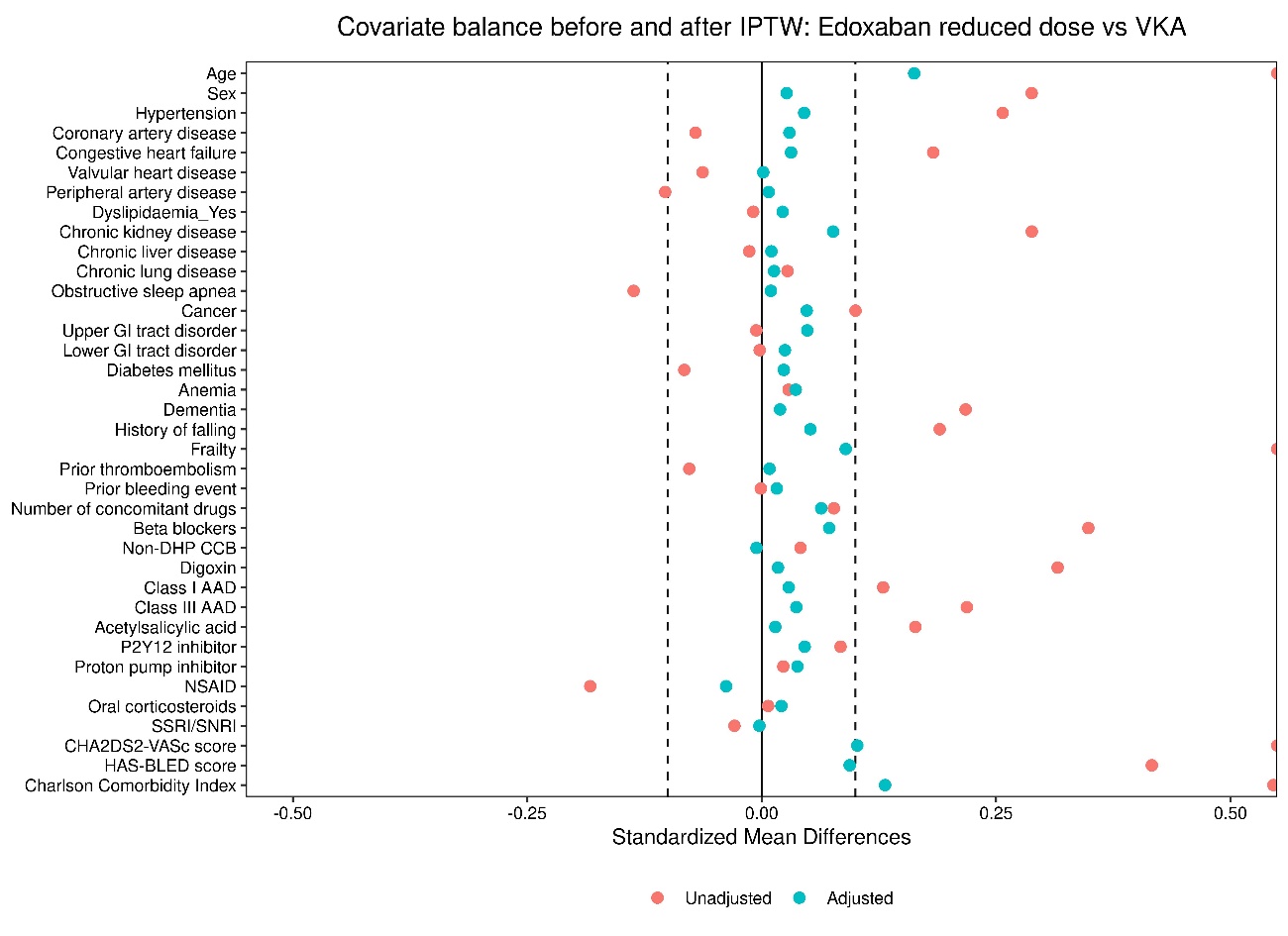


**F)**

**
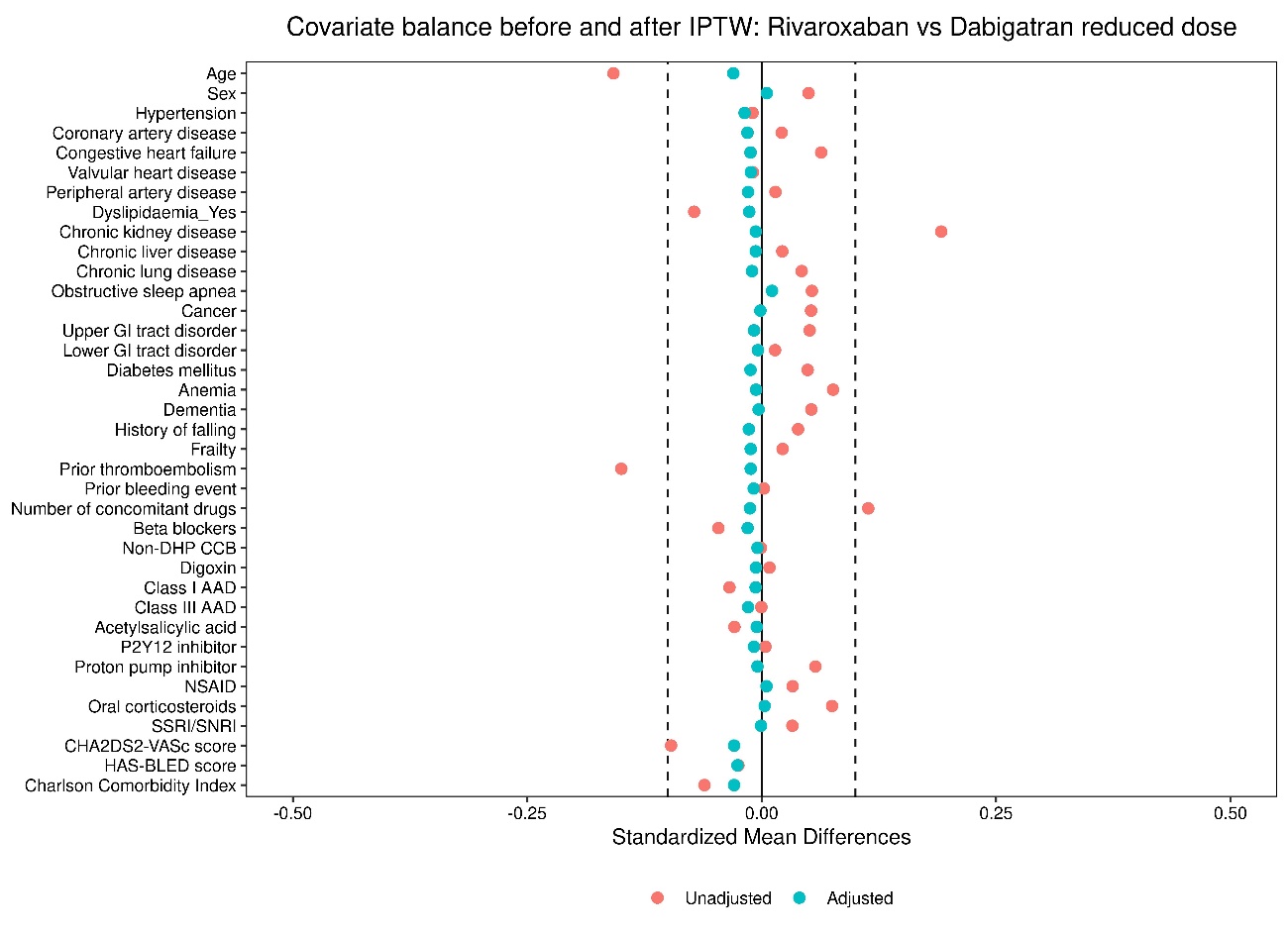
**

**G)**

**
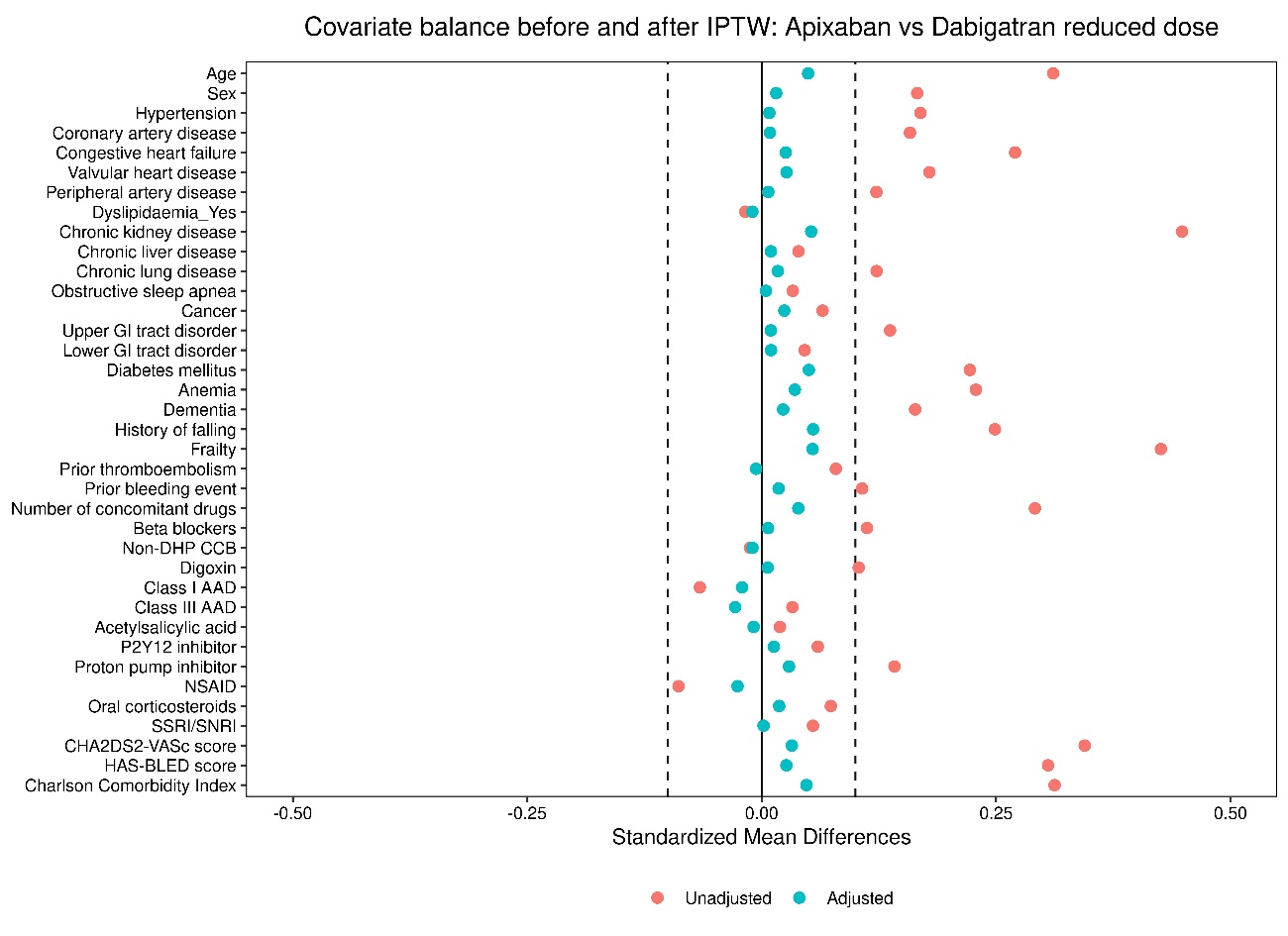
**

**H)**

**
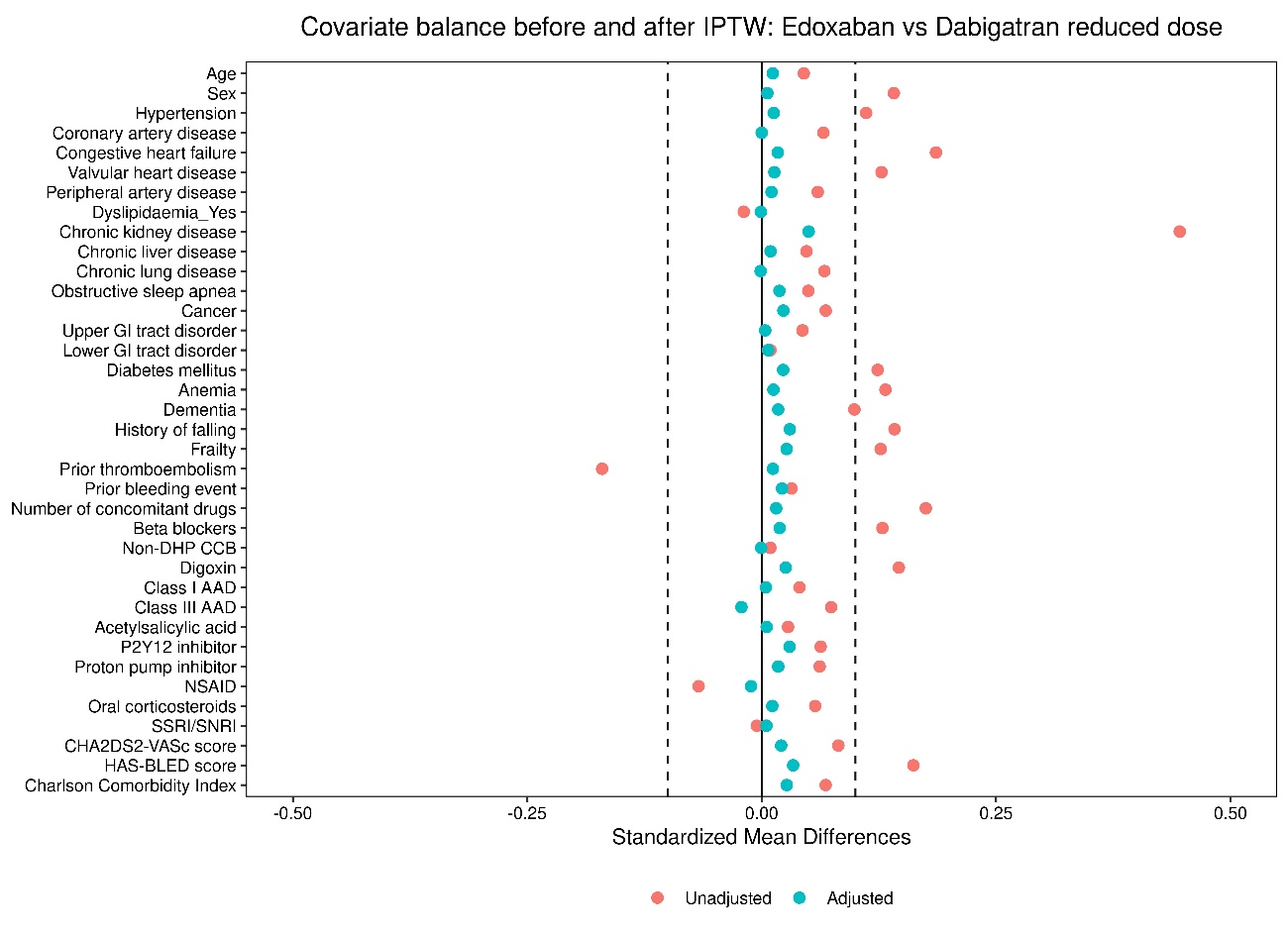
**

**I)**

**
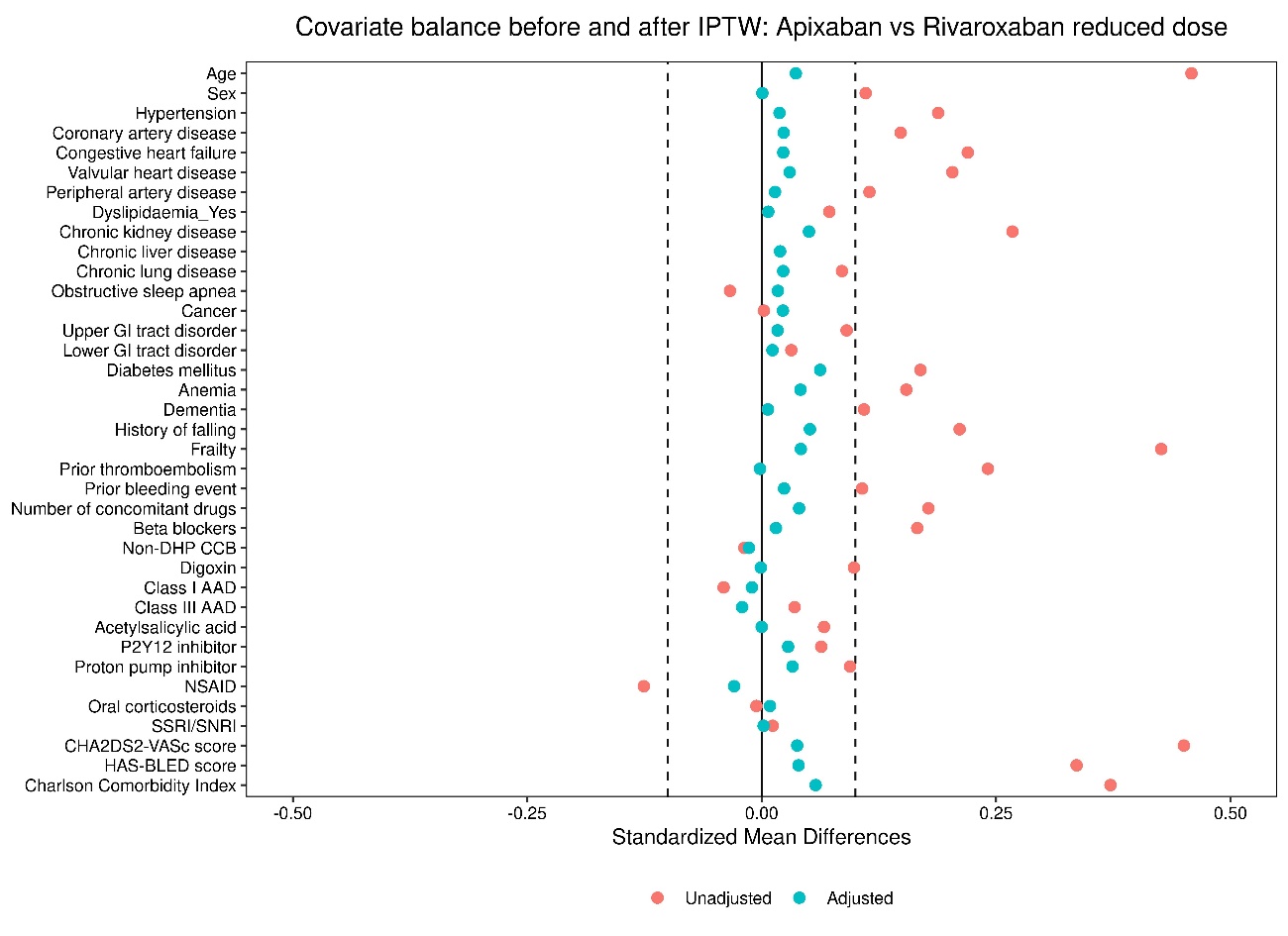
**

**J)**

**
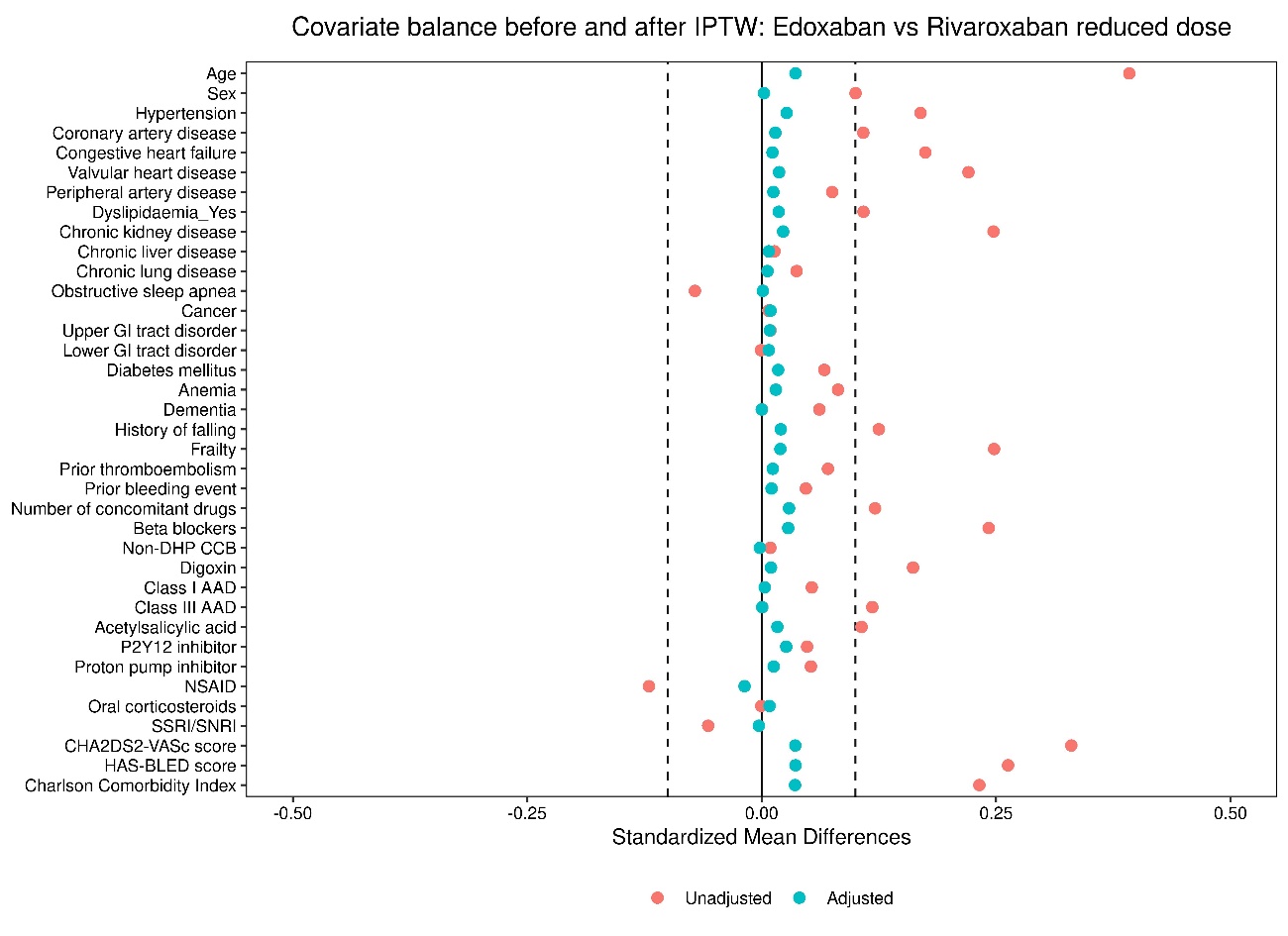
**

**K)**


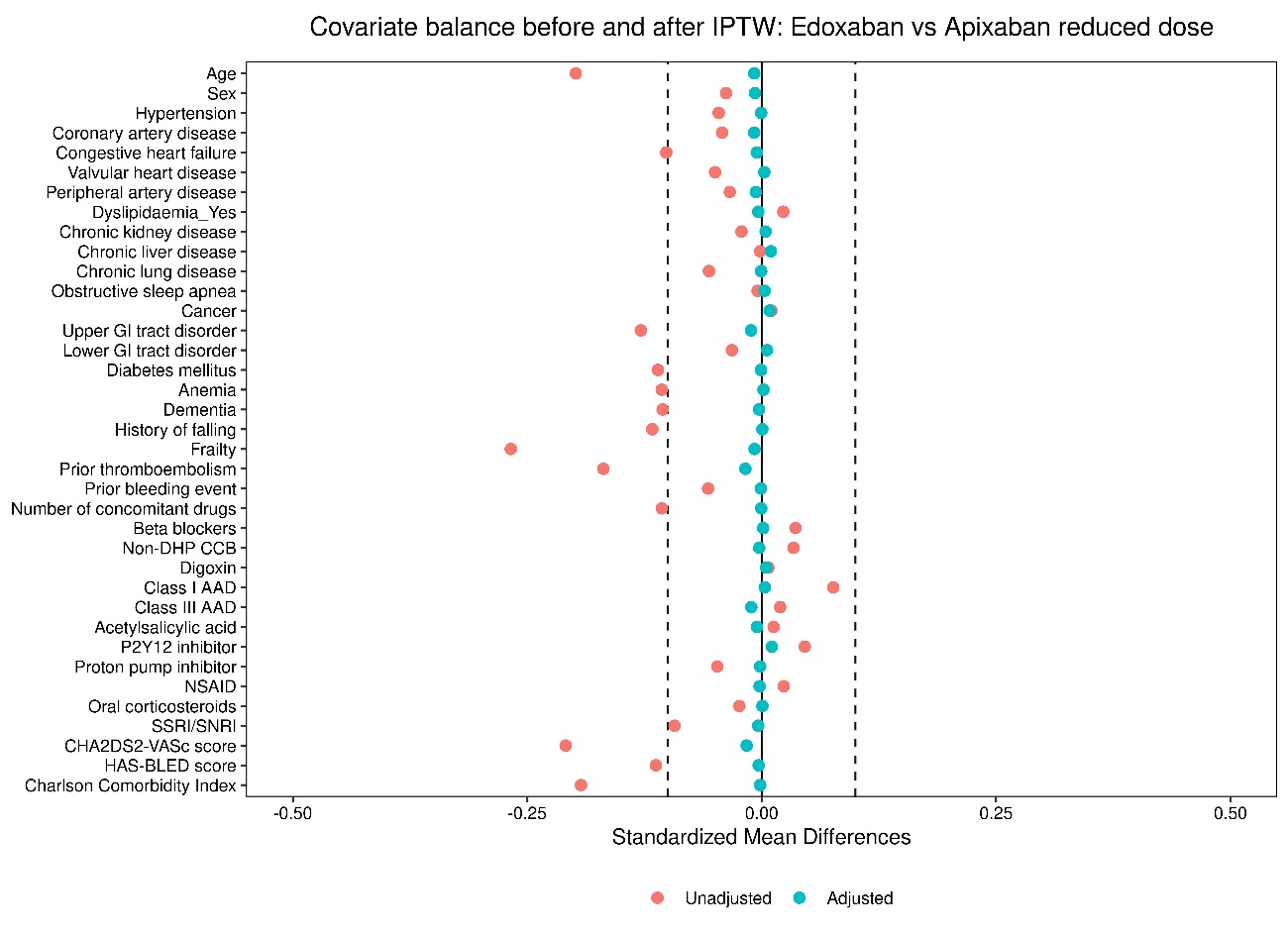


**eFigure 4:** Love plots illustrating covariate balance before and after IPTW when comparing **A)** reduced dose NOACs to VKAs, **B)** reduced dose dabigatran to VKAs, **C)** reduced dose rivaroxaban to VKAs, **D)** reduced dose apixaban to VKAs, **E)** reduced dose edoxaban to VKAs, **F)** reduced dose rivaroxaban to dabigatran, **G)** reduced dose apixaban to dabigatran, **H)** reduced dose edoxaban to dabigatran, **I)** reduced dose apixaban to rivaroxaban, **J)** reduced dose edoxaban to rivaroxaban, and **K)** reduced dose edoxaban to apixaban.

AAD: antiarrhythmic drug; GI: gastrointestinal; IPTW: inverse probability of treatment weighting; NOAC: non-vitamin K antagonist oral anticoagulant; NSAID: non-steroidal anti-inflammatory drug; OAC: oral anticoagulant; SE: systemic embolism; SNRI: serotonin and norepinephrine reuptake inhibitor; SSRI: selective serotonin reuptake inhibitor; VKA: vitamin K antagonist; vs: versus.

# References

1. Grymonprez M, Capiau A, Steurbaut S, Mehuys E, Boussery K, De Backer TL, et al. Adherence and persistence to oral anticoagulants in patients with atrial fibrillation: A Belgian nationwide cohort study. Front Cardiovasc Med. 2022;9.

2. Maetens A, De Schreye R, Faes K, Houttekier D, Deliens L, Gielen B, et al. Using linked administrative and disease-specific databases to study end-of-life care on a population level. BMC Palliat Care. 2016;15(1):86.

3. InterMutualistic Agency (IMA/AIM). Available from: <https://ima-aim.be/>. Accessed 25 November 2021.

4. The Minimal Hospital Dataset. Available from: <https://www.health.belgium.be/en/node/23607>. Accessed 25 November 2021.

5. The Sectoral Committee of Social Security and Health, Section Health ('Informatieveiligheidscomité'). Available from: <https://www.ehealth.fgov.be/ehealthplatform/nl/informatieveiligheidscomite>. Accessed 25 November 2021.

6. Go AS, Hylek EM, Chang Y, Phillips KA, Henault LE, Capra AM, et al. Anticoagulation therapy for stroke prevention in atrial fibrillation: how well do randomized trials translate into clinical practice? JAMA. 2003;290(20):2685-92.

7. Lin KJ, Schneeweiss S, Pawar A, Singer DE, Liu J, Gagne JJ. Using a Simple Prescription Gap to Determine Warfarin Discontinuation Can Lead to Substantial Misclassification. Thromb Haemost. 2022;122(3):386-93.

8. Zalesak M, Siu K, Francis K, Yu C, Alvrtsyan H, Rao Y, et al. Higher persistence in newly diagnosed nonvalvular atrial fibrillation patients treated with dabigatran versus warfarin. Circ Cardiovasc Qual Outcomes. 2013;6(5):567-74.

9. Ferroni E, Gennaro N, Costa G, Fedeli U, Denas G, Pengo V, et al. Real-world persistence with direct oral anticoagulants (DOACs) in naive patients with non-valvular atrial fibrillation. Int J Cardiol. 2019;288:72-5.

10. Martinez C, Katholing A, Wallenhorst C, Freedman SB. Therapy persistence in newly diagnosed non-valvular atrial fibrillation treated with warfarin or NOAC. A cohort study. Thromb Haemost. 2016;115(1):31-9.

11. Coleman CI, Tangirala M, Evers T. Treatment Persistence and Discontinuation with Rivaroxaban, Dabigatran, and Warfarin for Stroke Prevention in Patients with Non-Valvular Atrial Fibrillation in the United States. PLoS One. 2016;11(6):e0157769.

12. Hellfritzsch M, Grove EL, Husted SE, Rasmussen L, Poulsen BK, Johnsen SP, et al. Clinical events preceding switching and discontinuation of oral anticoagulant treatment in patients with atrial fibrillation. Europace. 2017;19(7):1091-5.

13. WHO Collaborating Centre for Drug Statistics Methodology. Available from: <https://www.whocc.no/>. Accessed 25 November 2021.

14. Pham PN, Brown JD. Real-world adherence for direct oral anticoagulants in a newly diagnosed atrial fibrillation cohort: does the dosing interval matter? BMC Cardiovasc Disord. 2019;19(1):64.

15. Sanfelix-Gimeno G, Rodriguez-Bernal CL, Hurtado I, Baixauli-Perez C, Librero J, Peiro S. Adherence to oral anticoagulants in patients with atrial fibrillation-a population-based retrospective cohort study linking health information systems in the Valencia region, Spain: a study protocol. BMJ Open. 2015;5(10):e007613.

16. Segal JB, Chang HY, Du Y, Walston JD, Carlson MC, Varadhan R. Development of a Claims-based Frailty Indicator Anchored to a Well-established Frailty Phenotype. Med Care. 2017;55(7):716-22.

17. Franchi C, Marcucci M, Mannucci PM, Tettamanti M, Pasina L, Fortino I, et al. Changes in clinical outcomes for community-dwelling older people exposed to incident chronic polypharmacy: a comparison between 2001 and 2009. Pharmacoepidemiol Drug Saf. 2016;25(2):204-11.

18. Quan H, Li B, Couris CM, Fushimi K, Graham P, Hider P, et al. Updating and validating the Charlson comorbidity index and score for risk adjustment in hospital discharge abstracts using data from 6 countries. Am J Epidemiol. 2011;173(6):676-82.

19. von Elm E, Altman DG, Egger M, Pocock SJ, Gøtzsche PC, Vandenbroucke JP. The Strengthening the Reporting of Observational Studies in Epidemiology (STROBE) statement: guidelines for reporting observational studies. Lancet. 2007;370(9596):1453-7.

20. The International Classification of Diseases (ICD), Clinical Modification. Available from: <https://www.cdc.gov/nchs/icd/index.htm>. Accessed 25 November 2021.

21. RIZIV/INAMI (Rijksinstituut voor ziekte- en invaliditeitsverzekering/Institut national d'assurance maladie-invalidité) medical procedure codes for claims of ambulatory and hospital care. Available from: <https://www.riziv.fgov.be/nl/nomenclatuur/Paginas/default.aspx> (in Dutch/French). Accessed 25 November 2021.

22. Hindricks G, Potpara T, Dagres N, Arbelo E, Bax JJ, Blomström-Lundqvist C, et al. 2020 ESC Guidelines for the diagnosis and management of atrial fibrillation developed in collaboration with the European Association of Cardio-Thoracic Surgery (EACTS). Eur Heart J. 2021;42(5):373-498.

23. Charlson ME, Pompei P, Ales KL, MacKenzie CR. A new method of classifying prognostic comorbidity in longitudinal studies: development and validation. J Chronic Dis. 1987;40(5):373-83.

24. Halvorsen S, Ghanima W, Fride Tvete I, Hoxmark C, Falck P, Solli O, et al. A nationwide registry study to compare bleeding rates in patients with atrial fibrillation being prescribed oral anticoagulants. Eur Heart J Cardiovasc Pharmacother. 2017;3(1):28-36.

25. Rutherford OW, Jonasson C, Ghanima W, Söderdahl F, Halvorsen S. Comparison of dabigatran, rivaroxaban, and apixaban for effectiveness and safety in atrial fibrillation: a nationwide cohort study. Eur Heart J Cardiovasc Pharmacother. 2020;6(2):75-85.
